# Supplementary material for: NAC couples protein synthesis with nascent polypeptide myristoylation on the ribosome
Source: EMBO J. 2025 Aug 26;44(22):6320–42. doi: 10.1038/s44318-025-00548-4 (PMC12623983; doi:10.1038/s44318-025-00548-4)
Supplement: Supplementary file 1 — Appendix [file 44318_2025_548_MOESM1_ESM.pdf]

Appendix for

NAC Couples Protein Synthesis with Nascent Polypeptide Myristoylation on the Ribosome

Table of contents

Appendix Figure S1…………………………………………………………………………….1

Appendix Figure S2…………………………………………………………………………….2

Appendix Figure S3…………………………………………………………………………….3

Appendix Figure S4…………………………………………………………………………….4

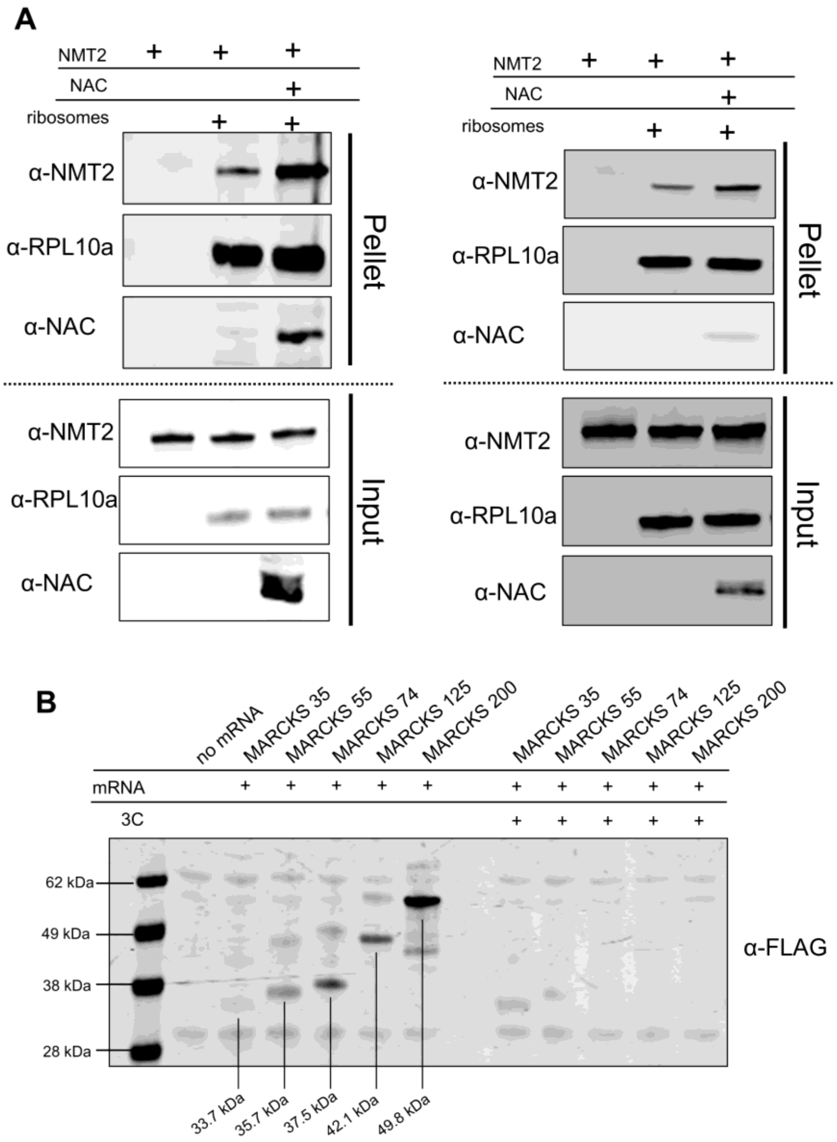

Appendix Figure S1. NMT2 dependency on NAC binding and generation of NMT2 substrate. A) Replicates of co-sedimentation performed between NMT2, NAC, and salt-washed ribosomes used for band intensity quantification in Figure 1. Input and pellet fractions are both shown. **B)** Immunoblot of in vitro translation reactions for MARCKS products. Bands can be seen for all length nascent chains and upon 3C treatment and cleavage of the FLAG tag, the bands disappear. The band for MARCKS 35 remains after protease treatment because the FLAG tag is still within the polypeptide exit tunnel and is protected from cleavage by 3C.

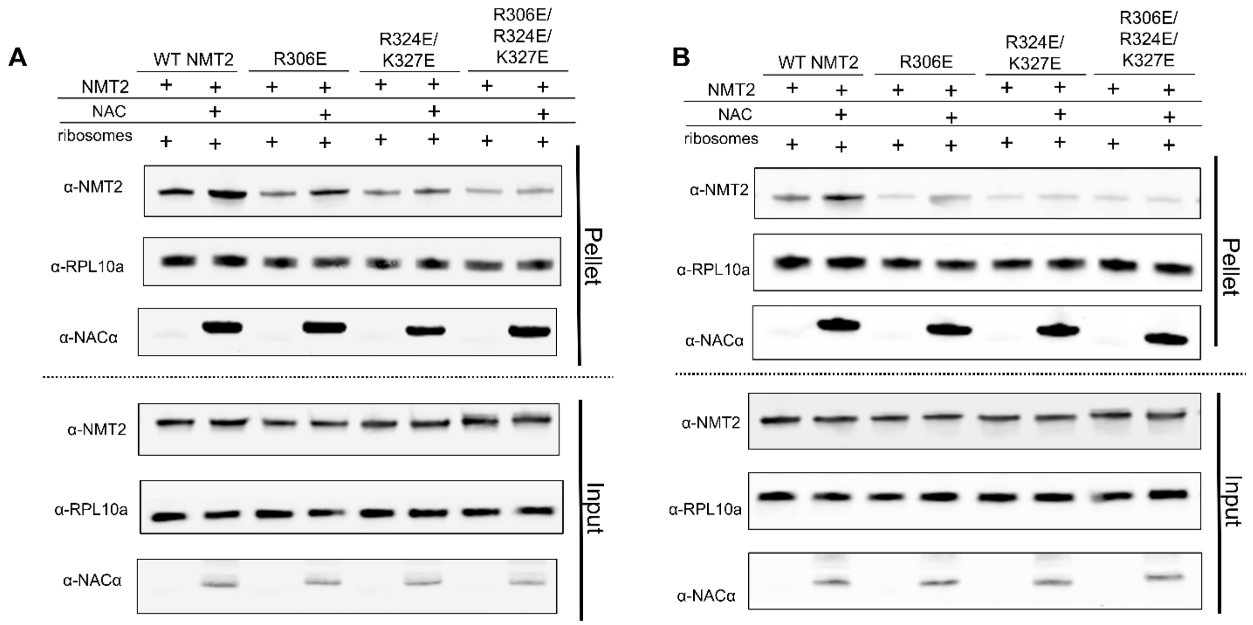

Appendix Figure S2. Replicates of co-sedimentation used for quantification in Figure 4. A) First replicate of the co-sedimentation assay with WT NMT2, R306E, R324E/K327E, and R306E/R324E/K327E used for quantification in Figures 4H and 4I. Both the input and pellet fractions are shown. **B)** Second replicate of the co-sedimentation assay with WT NMT2, R306E, R324E/K327E, and R306E/R324E/K327E used for quantification in Figures 4H and 4I. Both the input and pellet fractions are shown.

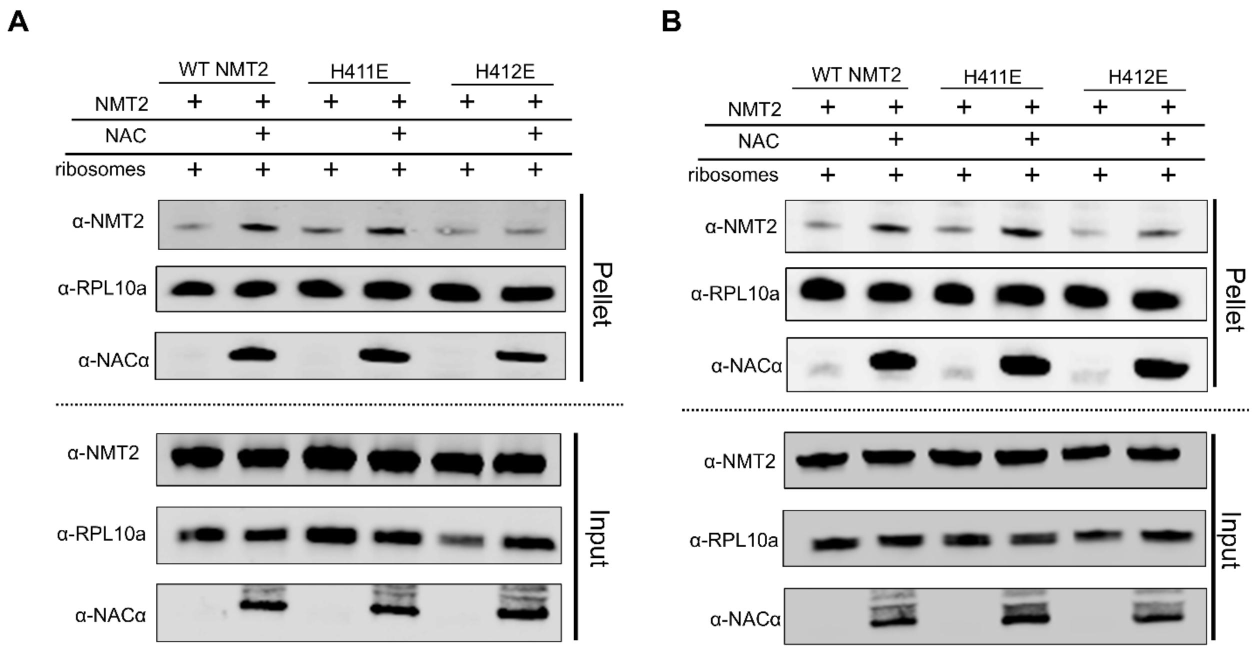

Appendix Figure S3. Replicates of co-sedimentation used for quantification in Figure 5. A) First replicate of the co-sedimentation assay with WT NMT2, H411E, and H412E used for quantification in Figures 5D and 5E. Both the input and pellet fractions are shown. **B)** Second replicate of the co-sedimentation assay with WT NMT2, H411E, and H412E used for quantification in Figures 5D and 5E. Both the input and pellet fractions are shown.

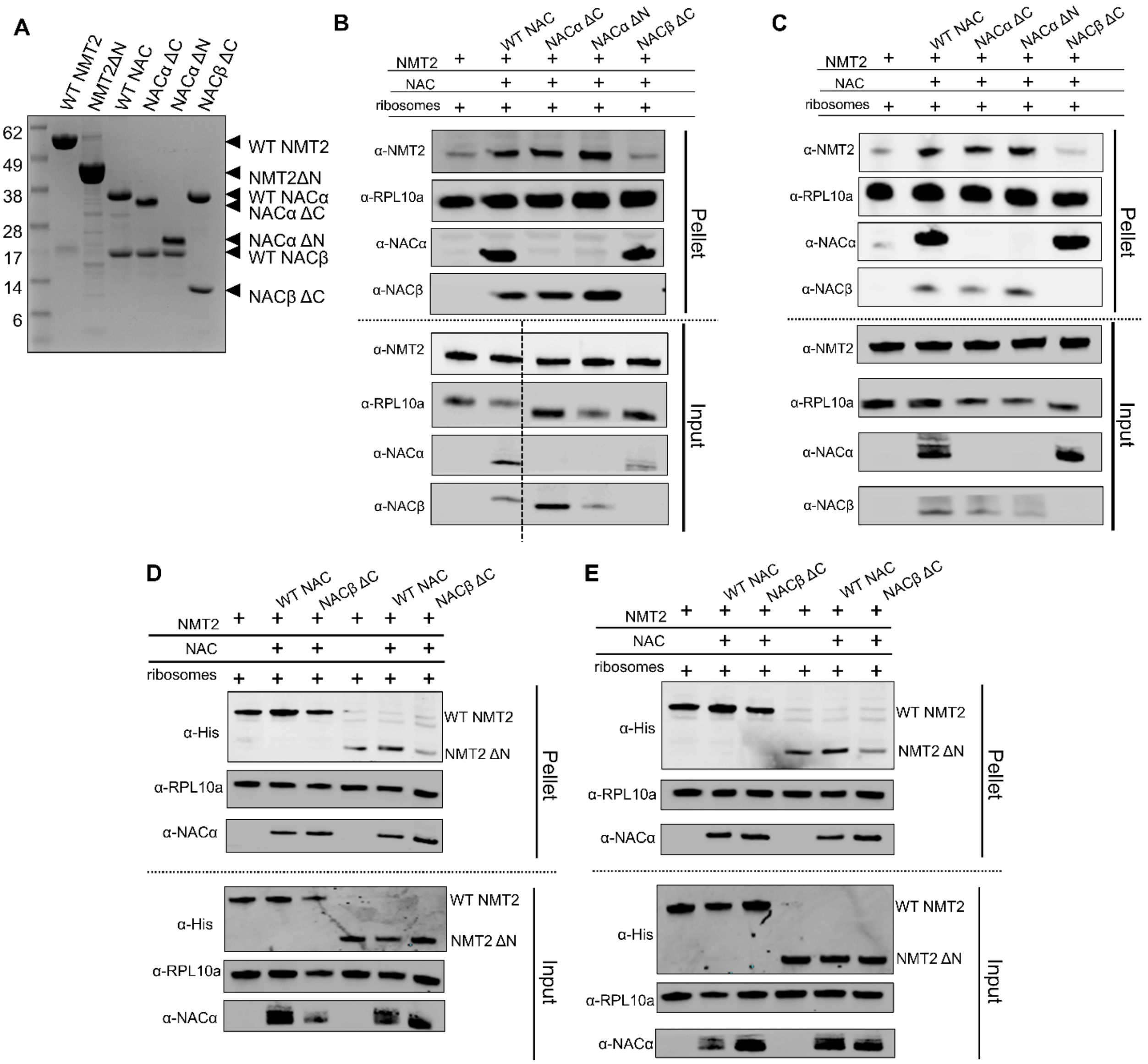

Appendix Figure S4. Validation of NAC truncation mutants and co-sedimentation replicates for quantification in Figure 6. A) Coomassie stained gel of purified WT NMT2, NMT2ΔN, and NAC tail truncation mutants. Both NAC α and β components are present in the soluble heterodimeric complex for all generated constructs. **B)** First replicate of the co-sedimentation assay with WT NMT2 and the NAC truncation mutants used for quantification in Figure 6E. Both the input and pellet fractions are shown. **C)** Second replicate of the co-sedimentation assay with WT NMT2 and the NAC truncation mutants used for quantification in

Figure 6E. Both the input and pellet fractions are shown. **D)** First replicate of the co-sedimentation assay between WT NMT2, NMT2ΔN and WT NAC, and NACβΔC used for quantification in Figure 6F. Both the input and pellet fractions are shown. **E)** Second replicate of the co-sedimentation assay between WT NMT2, NMT2ΔN and WT NAC, and NACβΔC used for quantification in Figure 6F. Both the input and pellet fractions are shown.
