# Supplementary figures and images for: NAC couples protein synthesis with nascent polypeptide myristoylation on the ribosome
Source: EMBO J. 2025 Aug 26;44(22):6320–42. doi: 10.1038/s44318-025-00548-4 (PMC12623983; doi:10.1038/s44318-025-00548-4)

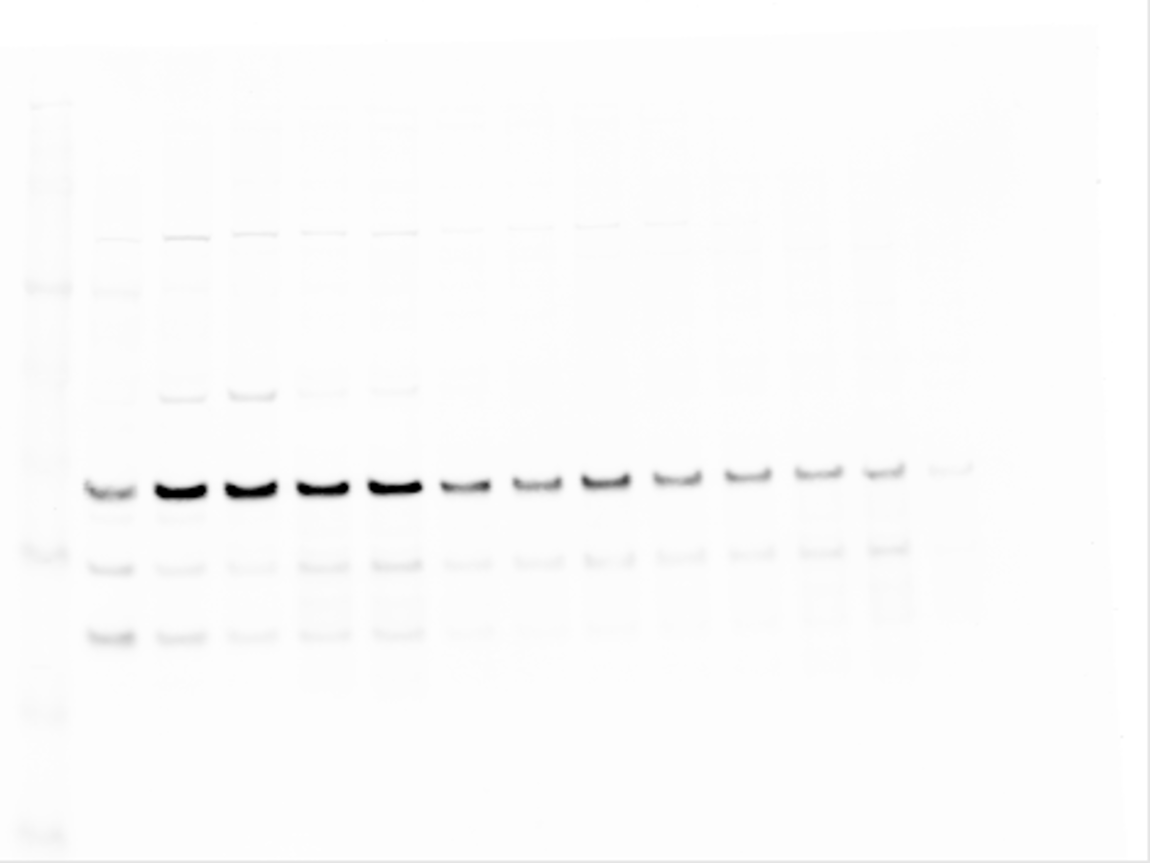

Supplement: Supplementary file 3 — Source data Fig. 1 [file 44318_2025_548_MOESM3_ESM.zip › EMBO-J-2025-120636_SourceDataFigure1/Panel B/polysome-NACa-blot.tif]

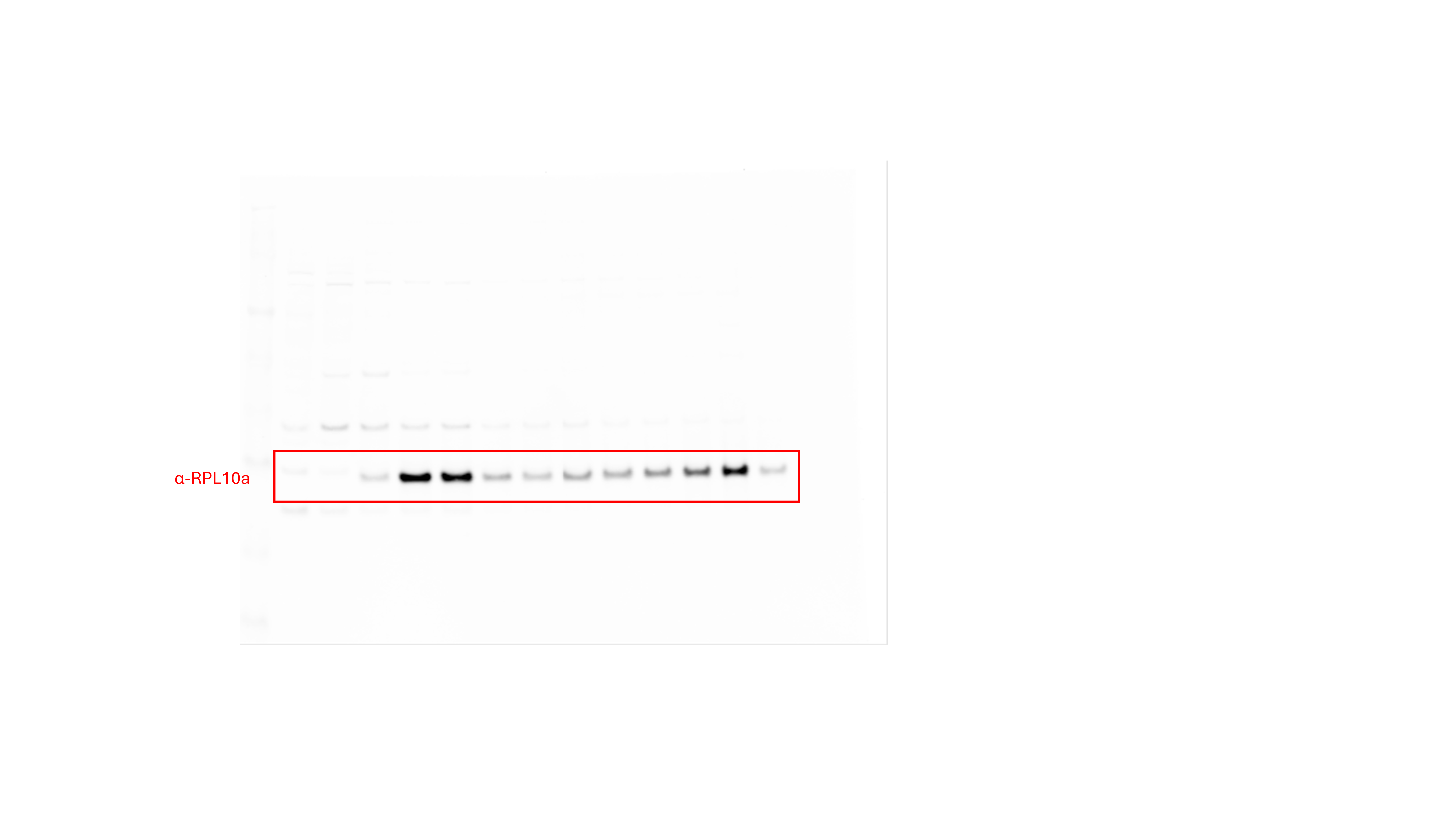

Supplement: Supplementary file 3 — Source data Fig. 1 [file 44318_2025_548_MOESM3_ESM.zip › EMBO-J-2025-120636_SourceDataFigure1/Panel B/Panel B RPL10a blot annotated.png]

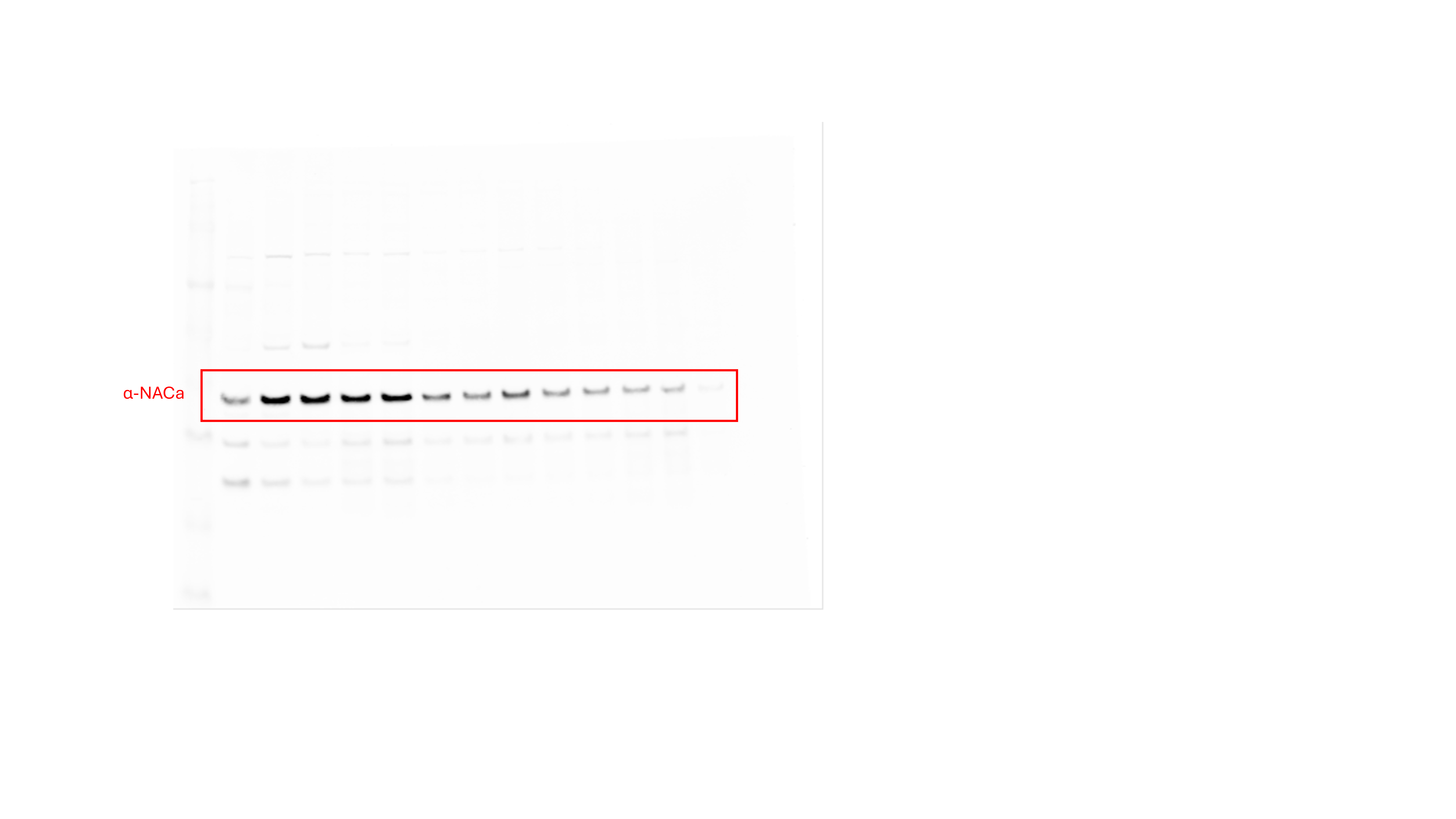

Supplement: Supplementary file 3 — Source data Fig. 1 [file 44318_2025_548_MOESM3_ESM.zip › EMBO-J-2025-120636_SourceDataFigure1/Panel B/Panel B NAC blot annotated.png]

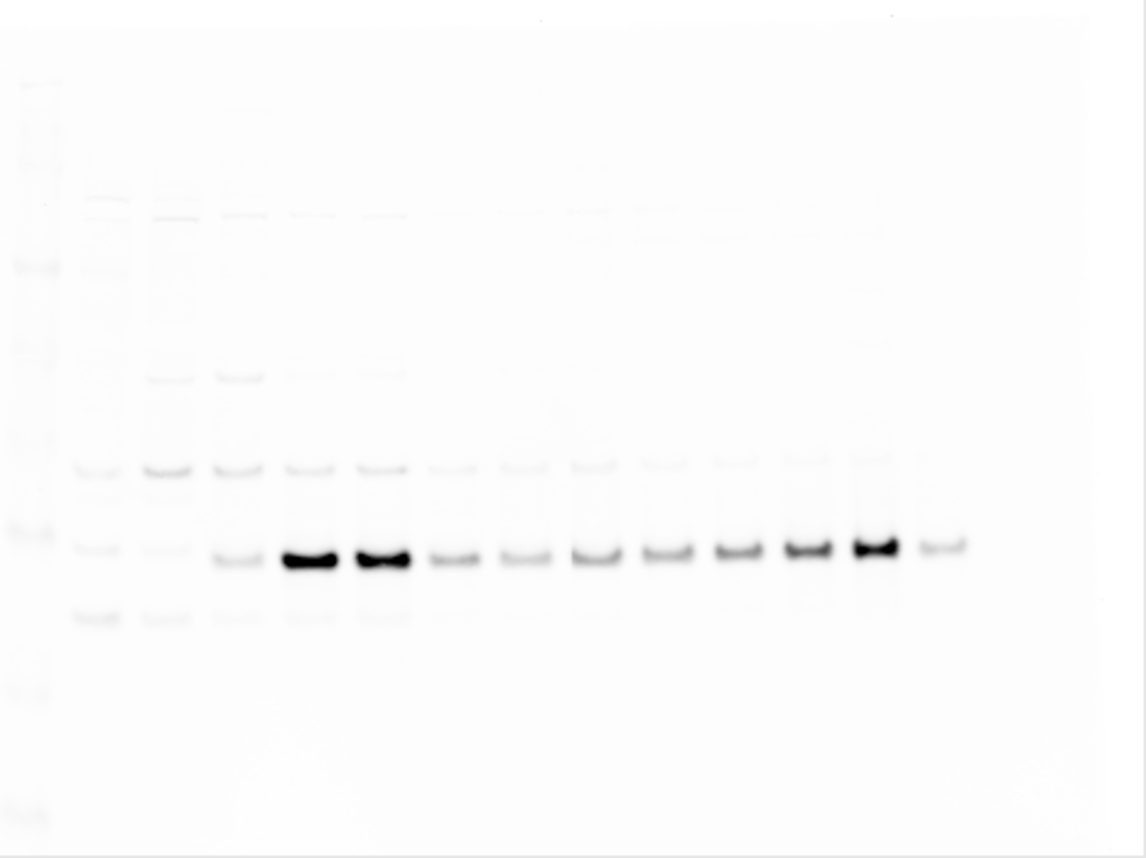

Supplement: Supplementary file 3 — Source data Fig. 1 [file 44318_2025_548_MOESM3_ESM.zip › EMBO-J-2025-120636_SourceDataFigure1/Panel B/polysome-RPL10a-blot.tif]

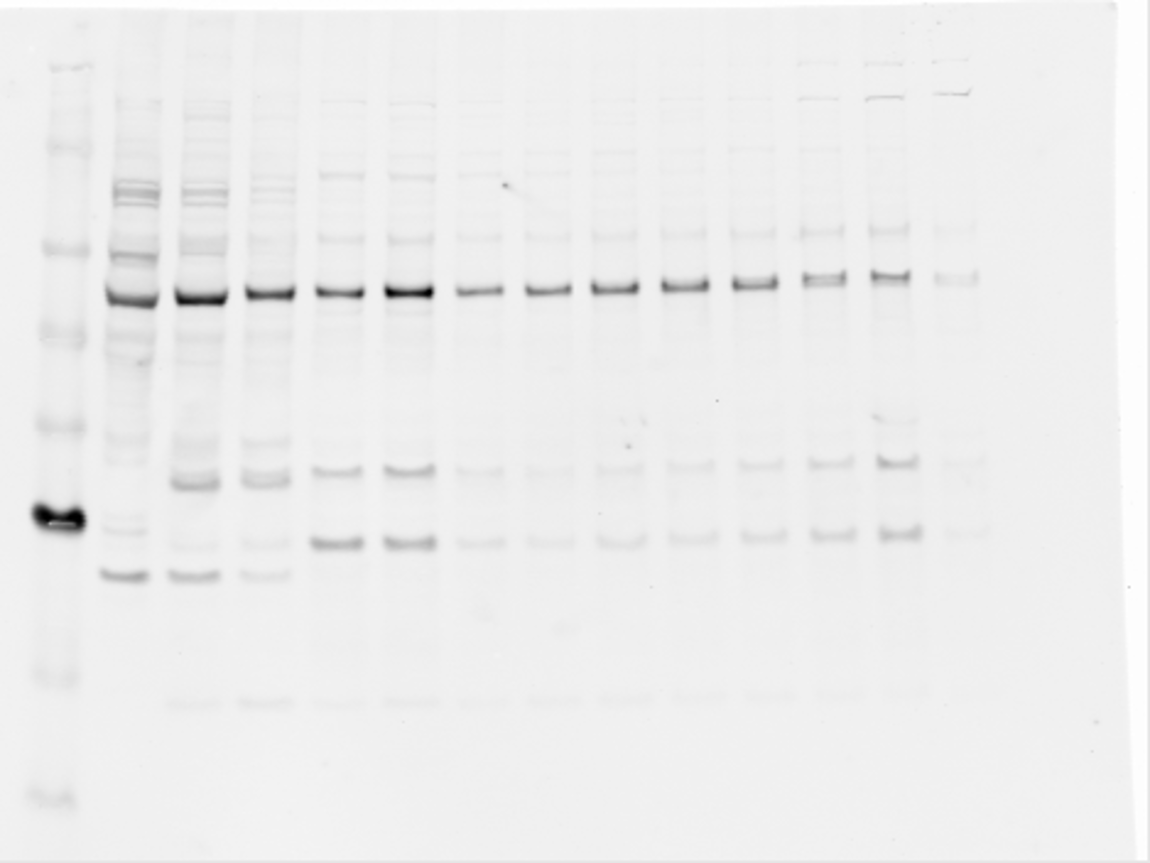

Supplement: Supplementary file 3 — Source data Fig. 1 [file 44318_2025_548_MOESM3_ESM.zip › EMBO-J-2025-120636_SourceDataFigure1/Panel B/polysome-NMT2-blot.tif]

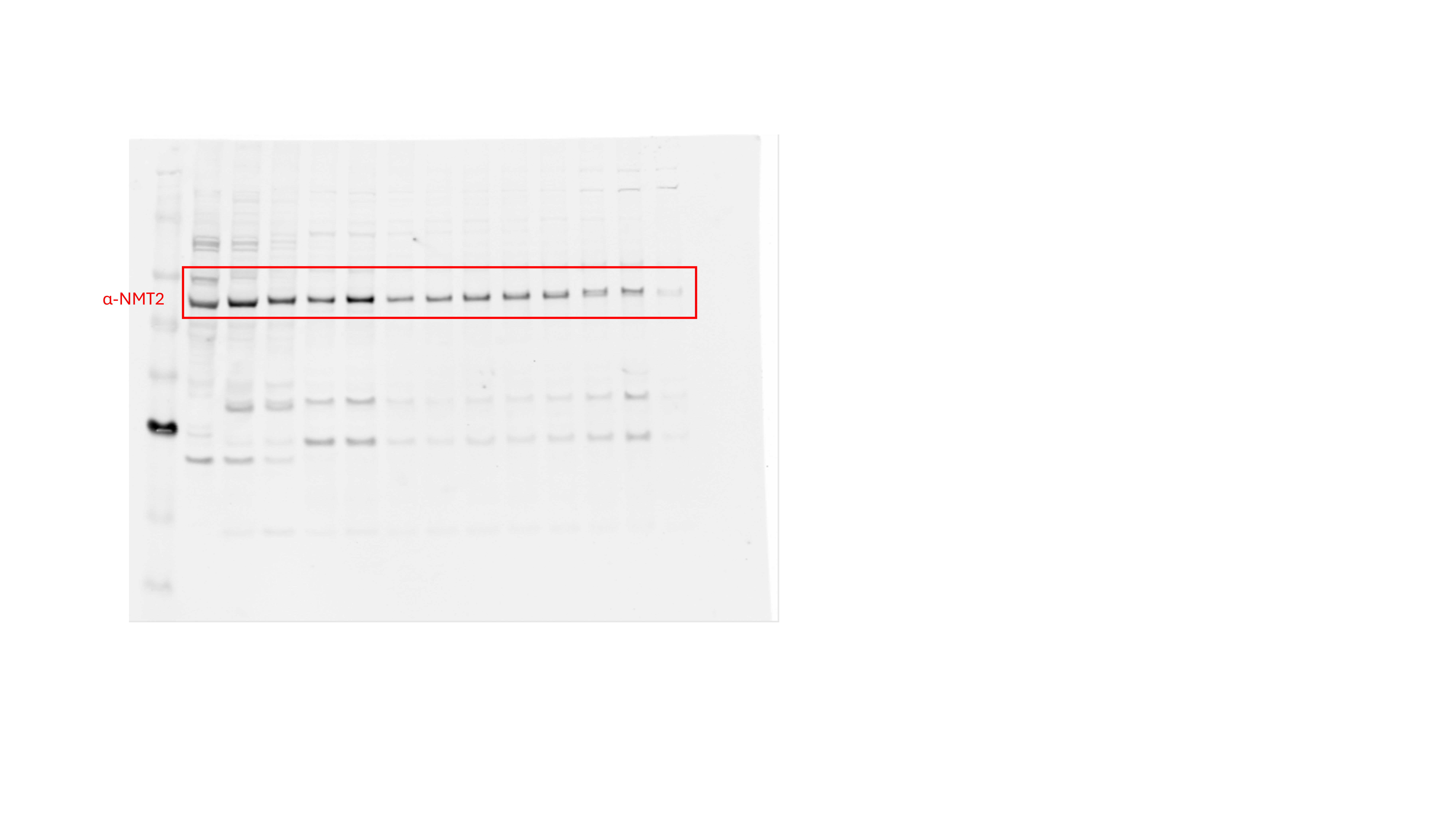

Supplement: Supplementary file 3 — Source data Fig. 1 [file 44318_2025_548_MOESM3_ESM.zip › EMBO-J-2025-120636_SourceDataFigure1/Panel B/Panel B NMT2 blot annotated.png]

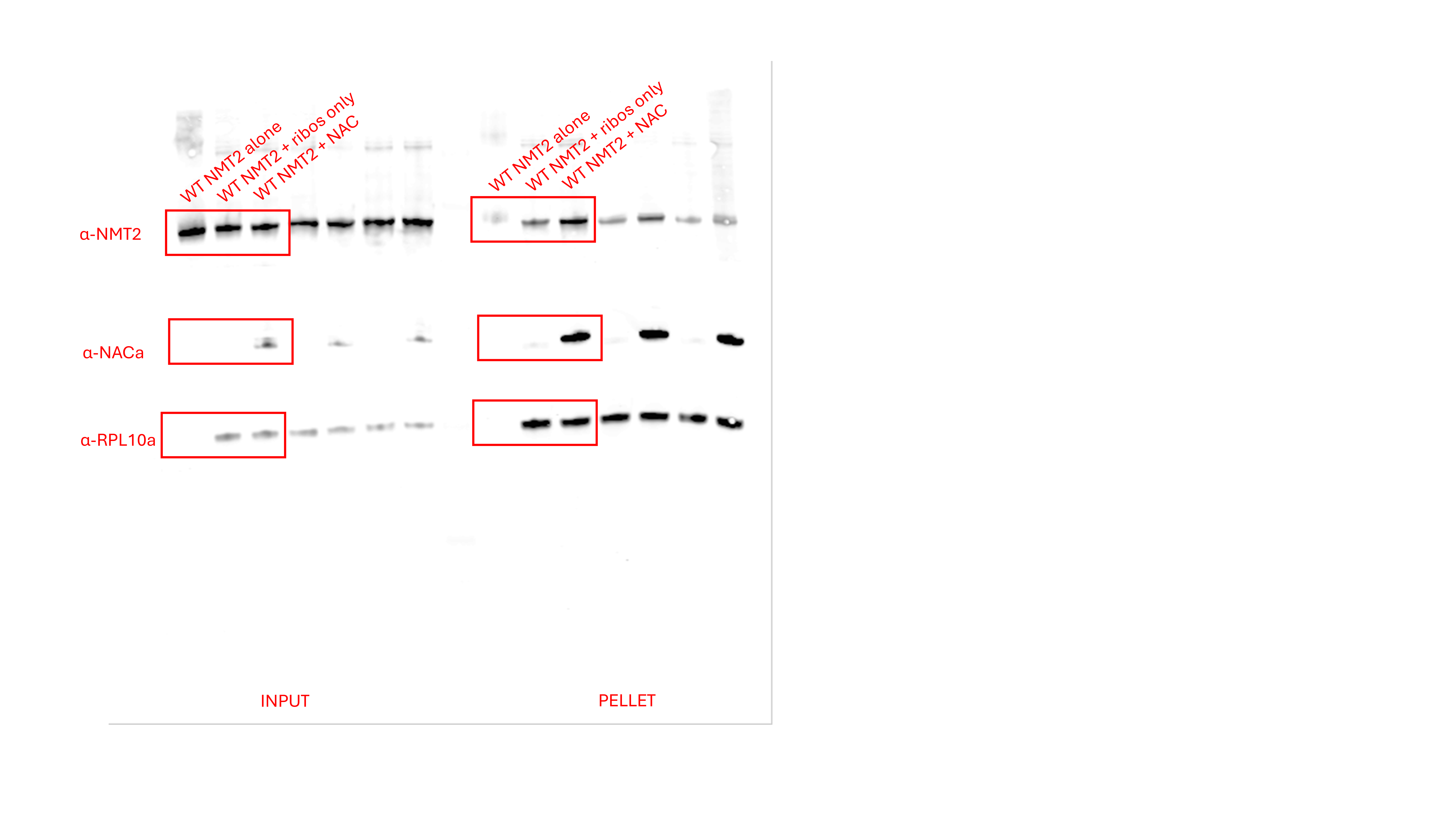

Supplement: Supplementary file 3 — Source data Fig. 1 [file 44318_2025_548_MOESM3_ESM.zip › EMBO-J-2025-120636_SourceDataFigure1/Panel C/Panel C input + pellet WB annotated.png]

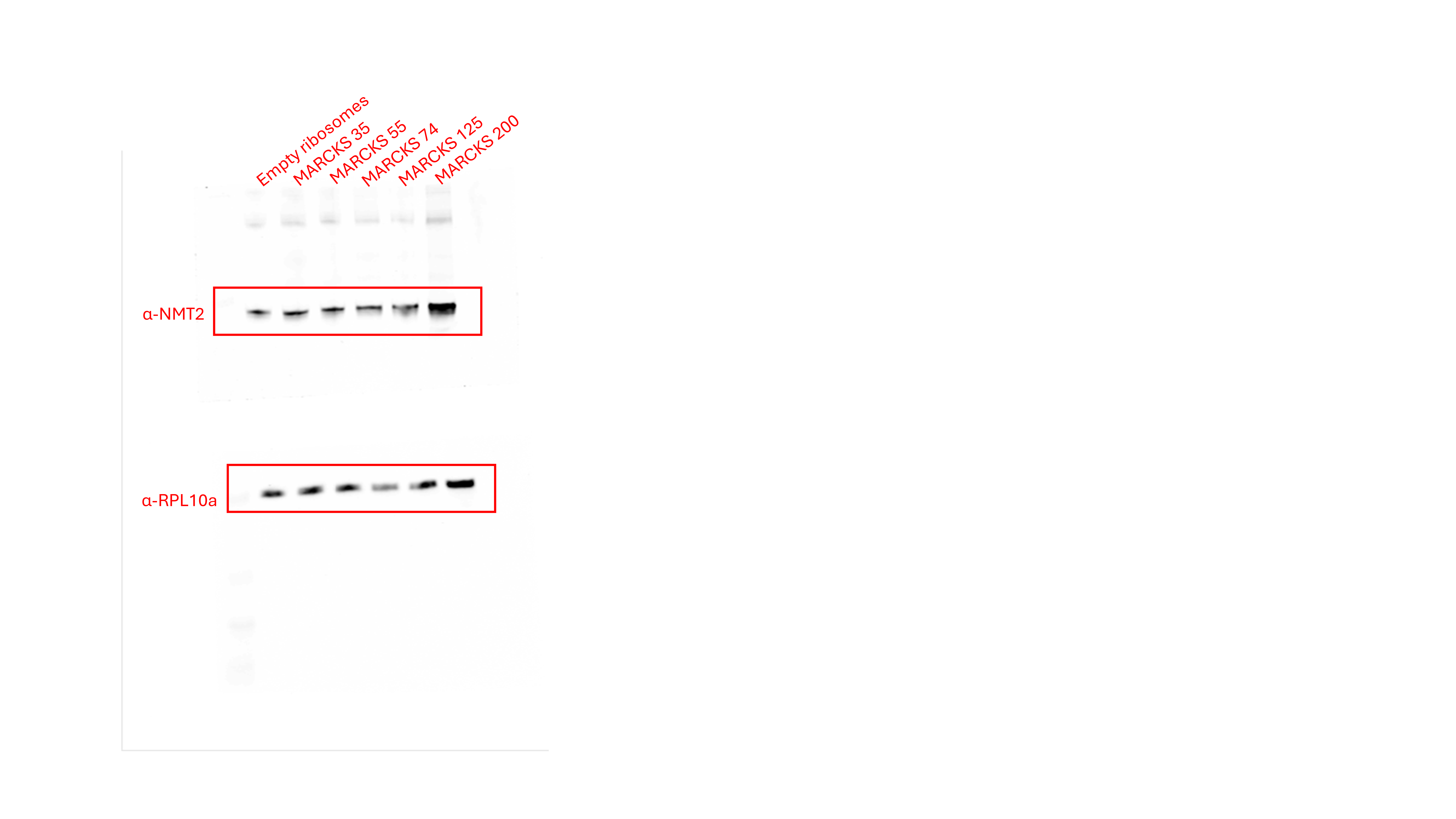

Supplement: Supplementary file 3 — Source data Fig. 1 [file 44318_2025_548_MOESM3_ESM.zip › EMBO-J-2025-120636_SourceDataFigure1/Panel F/Panel F WB annotated.png]

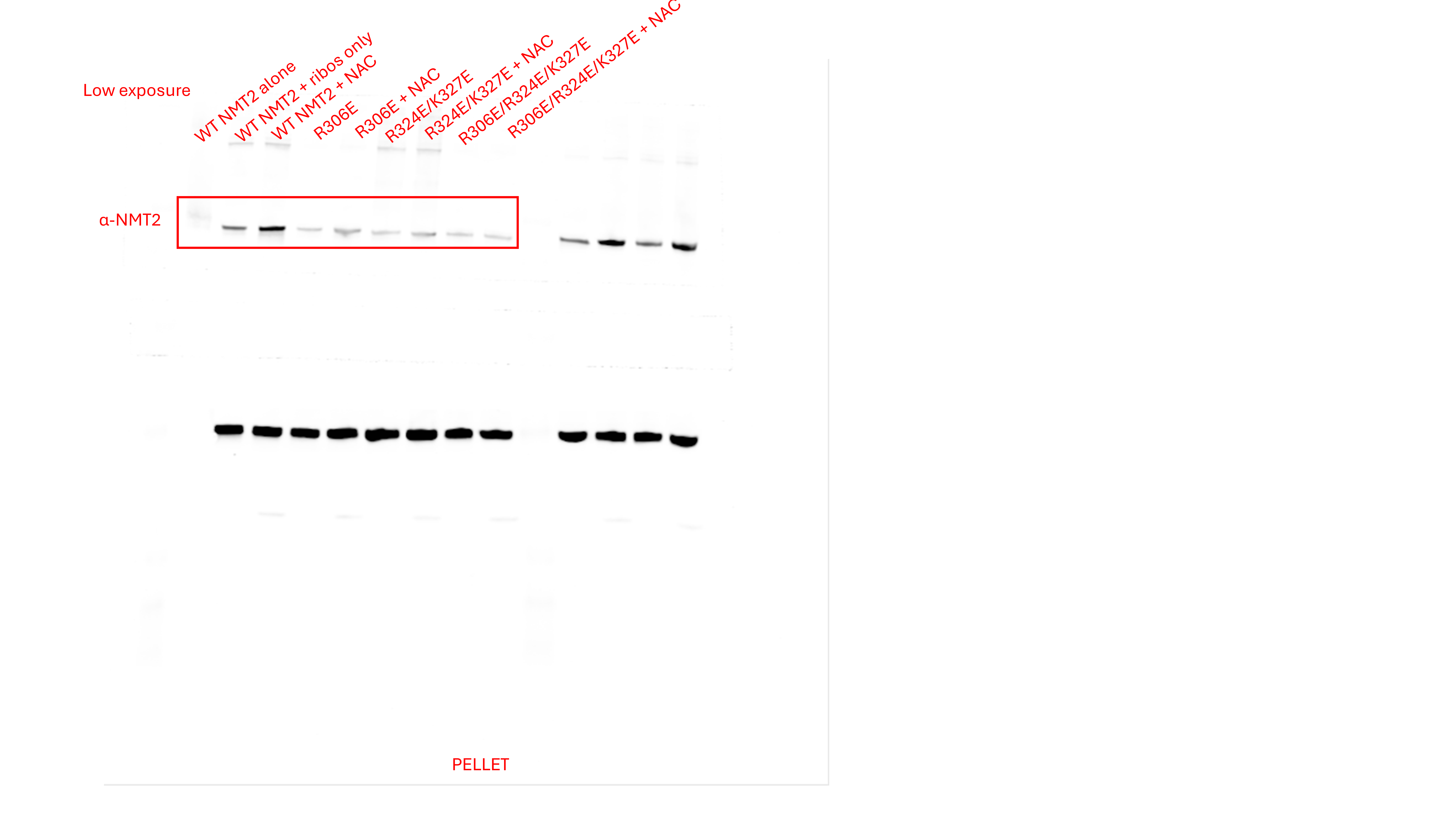

Supplement: Supplementary file 4 — Source data Fig. 4 [file 44318_2025_548_MOESM4_ESM.zip › EMBO-J-20205-120636_SourceDataFigure4/Panel G/Panel G pellet WB low exposure annotated.png]

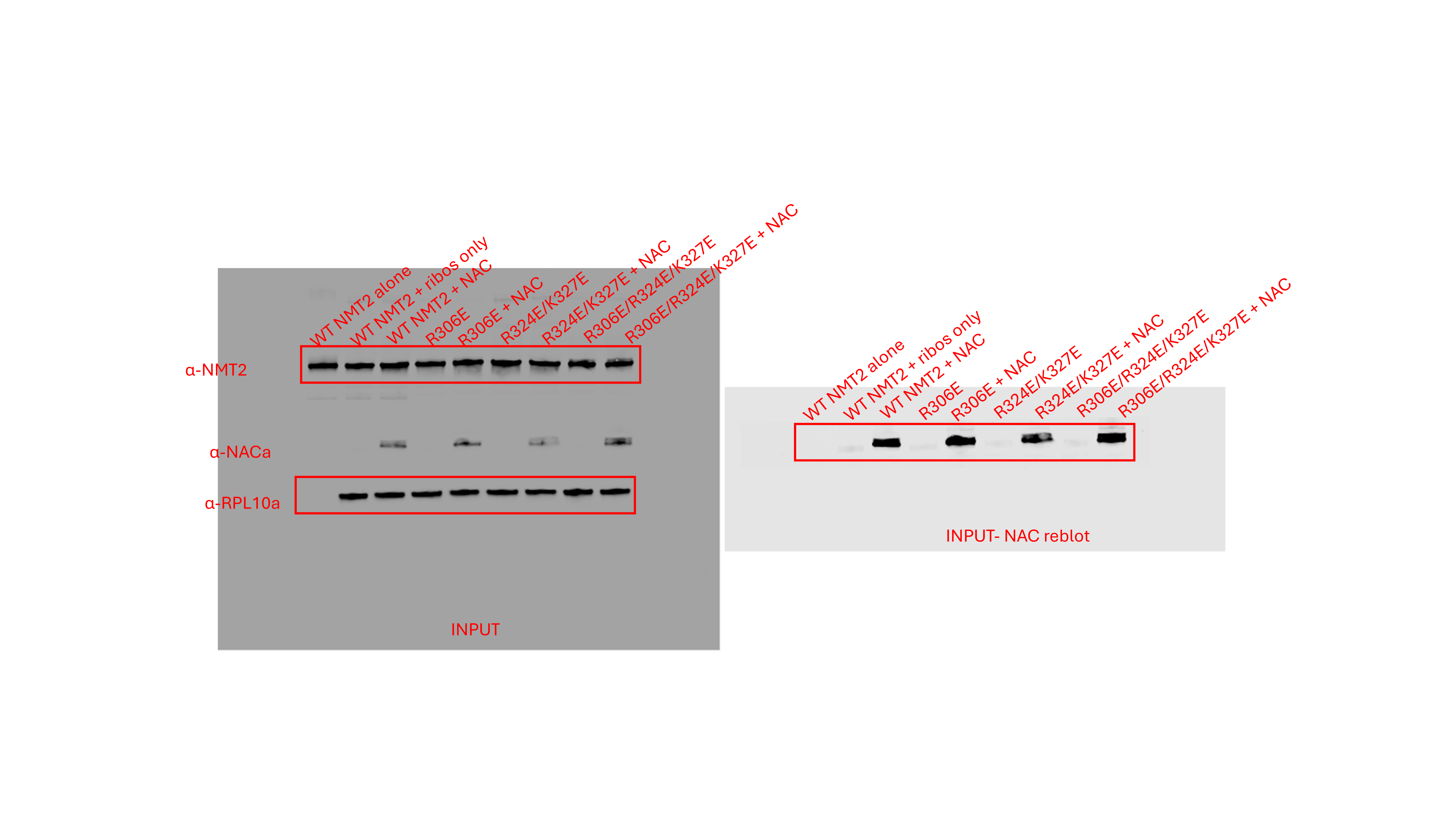

Supplement: Supplementary file 4 — Source data Fig. 4 [file 44318_2025_548_MOESM4_ESM.zip › EMBO-J-20205-120636_SourceDataFigure4/Panel G/Panel G input WB annotated.png]

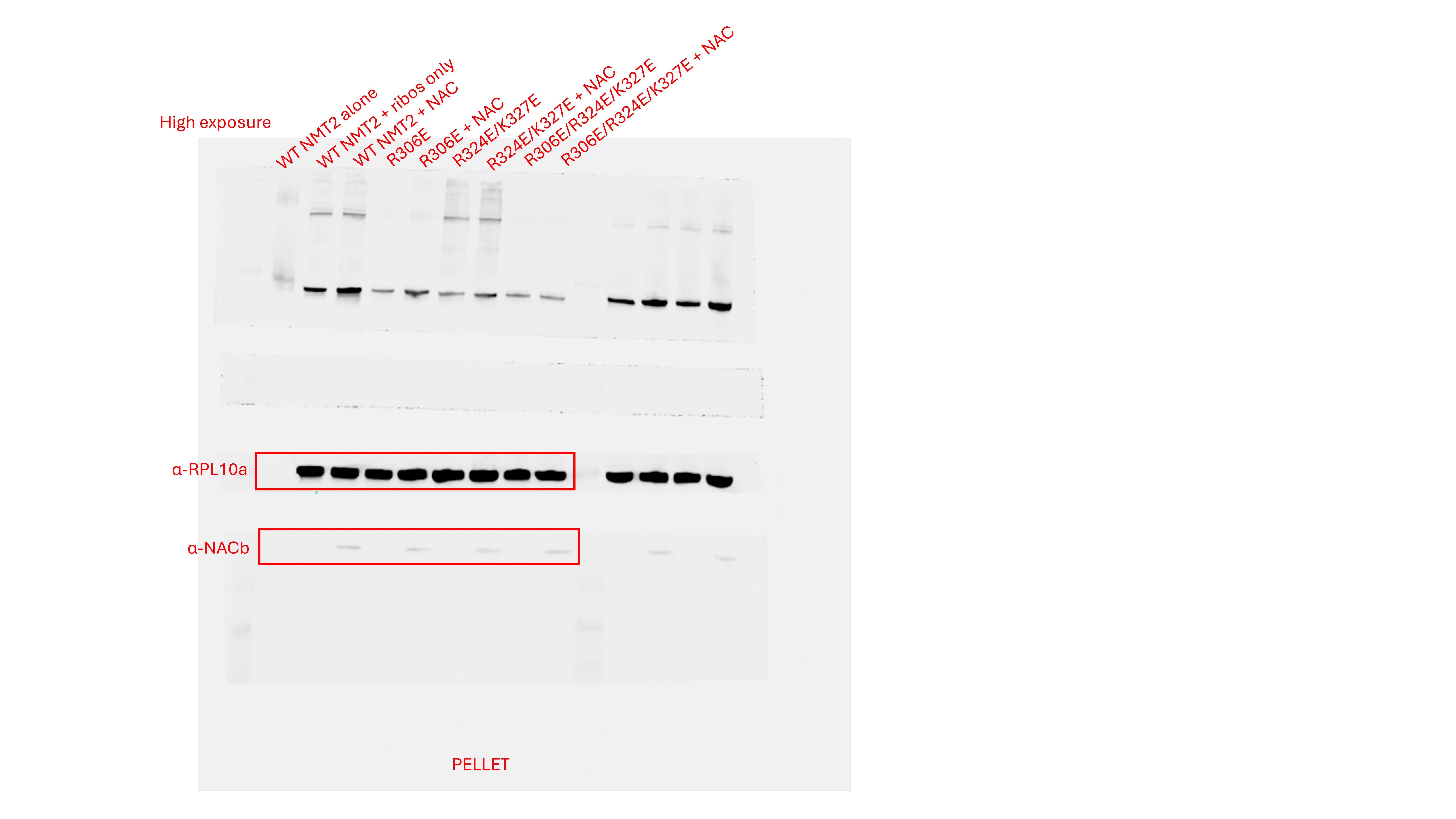

Supplement: Supplementary file 4 — Source data Fig. 4 [file 44318_2025_548_MOESM4_ESM.zip › EMBO-J-20205-120636_SourceDataFigure4/Panel G/Panel G pellet WB high exposure annotated.png]

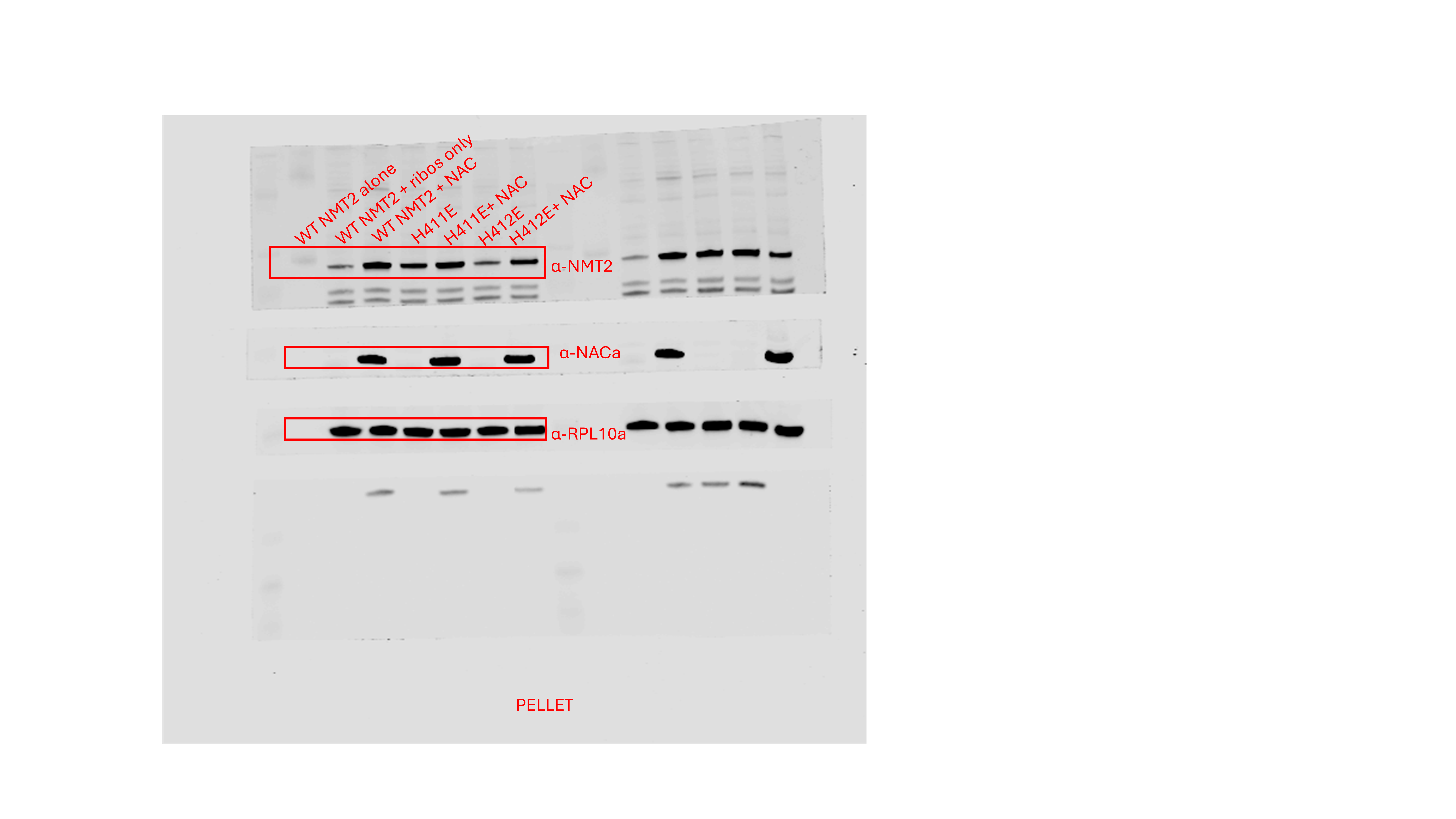

Supplement: Supplementary file 5 — Source data Fig. 5 [file 44318_2025_548_MOESM5_ESM.zip › EMBO-J-20205-120636_SourceDataFigure5/Panel C/Panel C pellet WB annotated.png]

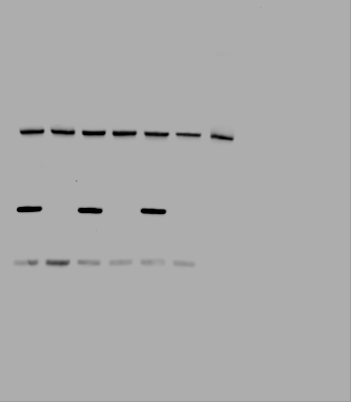

Supplement: Supplementary file 5 — Source data Fig. 5 [file 44318_2025_548_MOESM5_ESM.zip › EMBO-J-20205-120636_SourceDataFigure5/Panel C/input WB raw.tif]

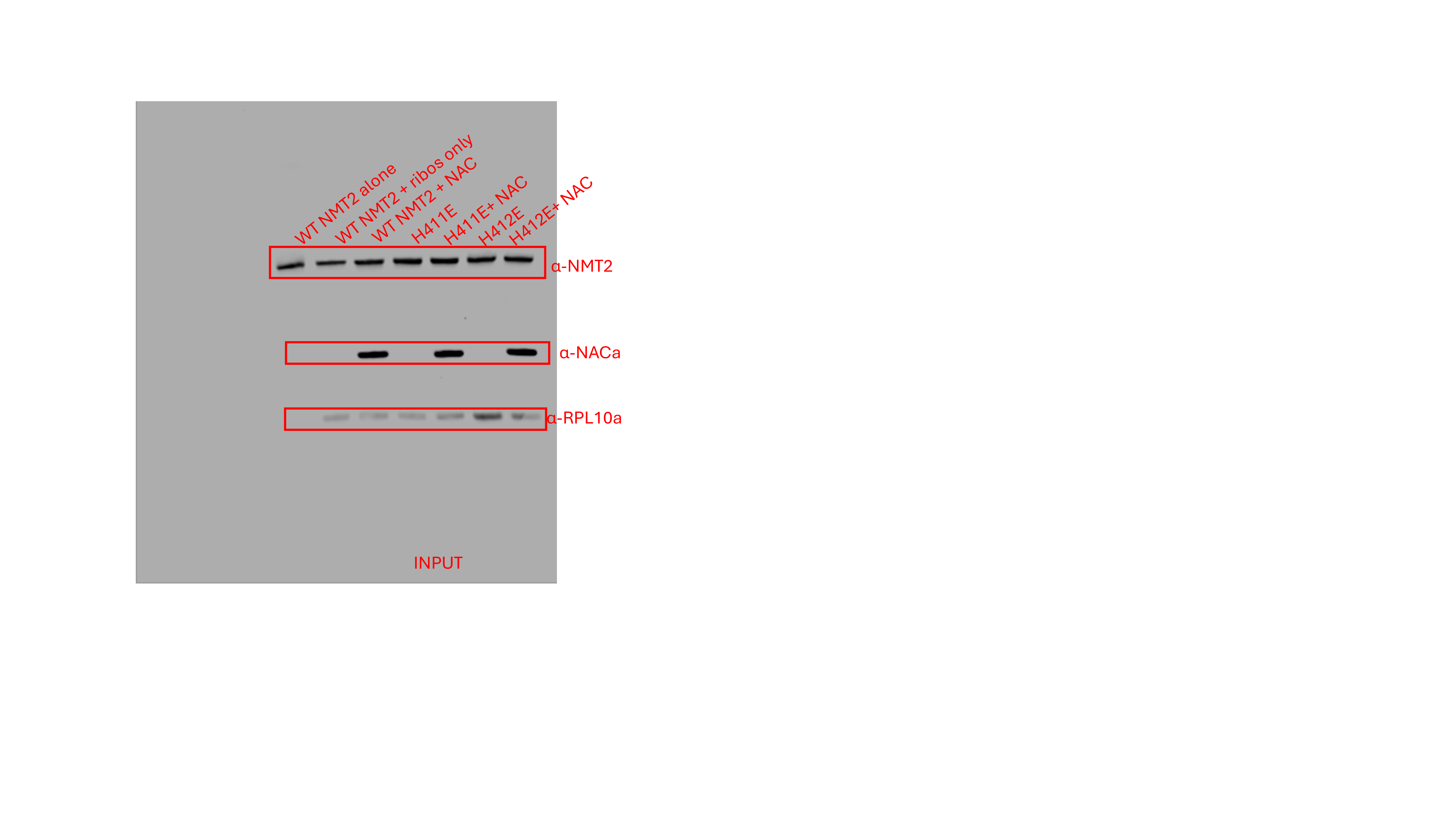

Supplement: Supplementary file 5 — Source data Fig. 5 [file 44318_2025_548_MOESM5_ESM.zip › EMBO-J-20205-120636_SourceDataFigure5/Panel C/Panel C input WB annotated.png]

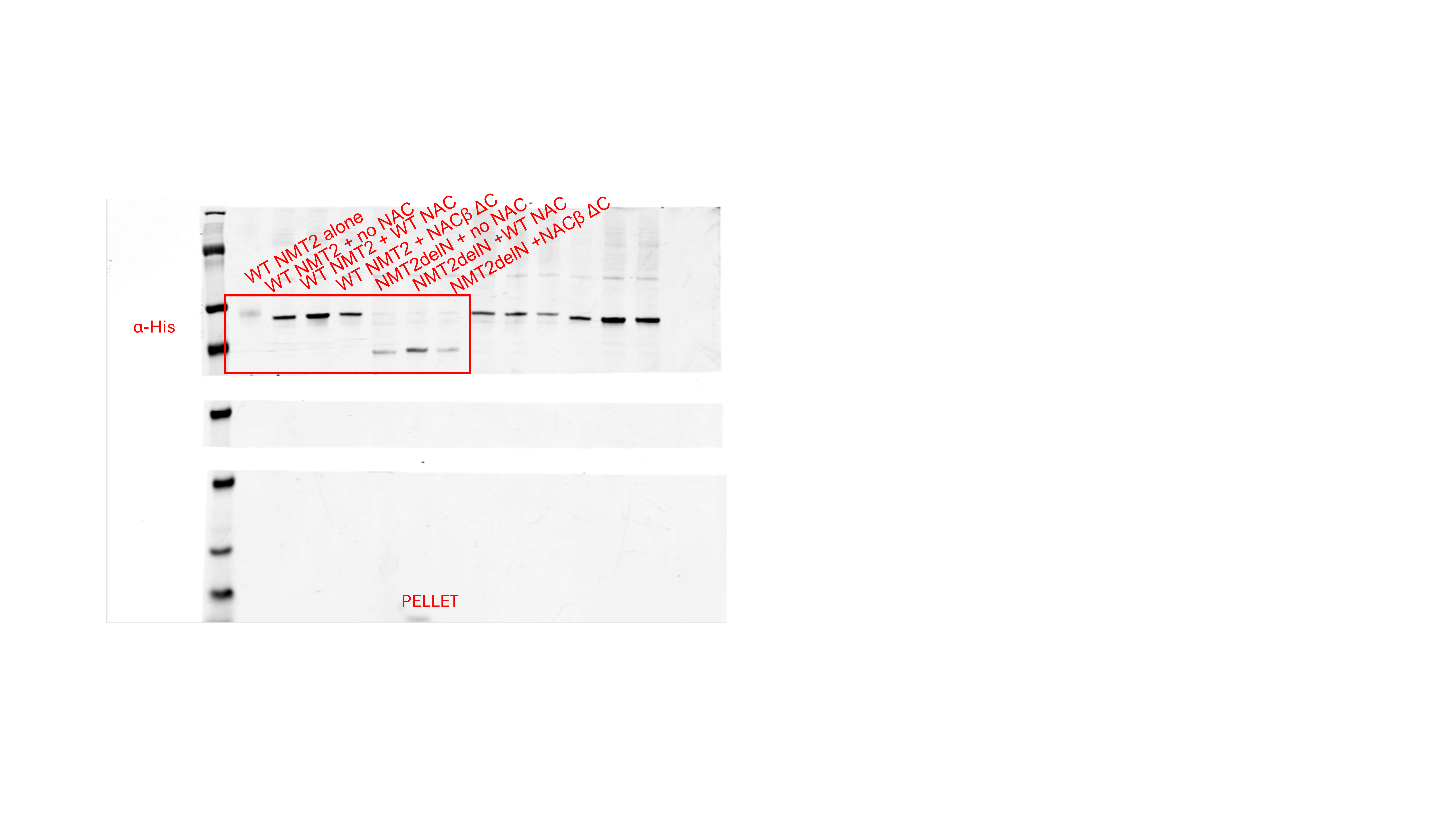

Supplement: Supplementary file 6 — Source data Fig. 6 [file 44318_2025_548_MOESM6_ESM.zip › EMBO-J-20205-120636_SourceDataFigure6/Panel E/Panel E pellet WB 700 annotated.png]

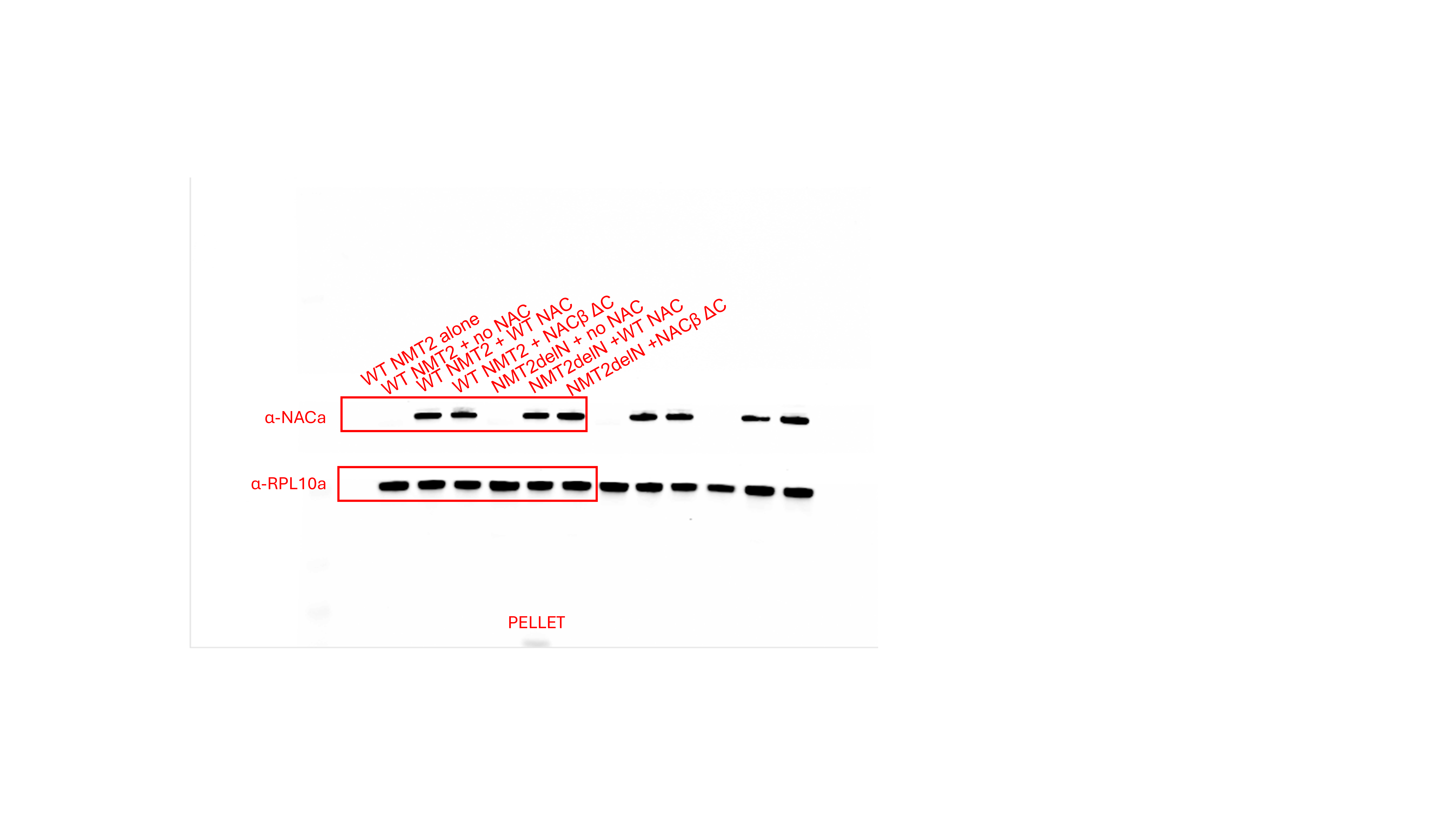

Supplement: Supplementary file 6 — Source data Fig. 6 [file 44318_2025_548_MOESM6_ESM.zip › EMBO-J-20205-120636_SourceDataFigure6/Panel E/Panel E pellet WB 800 annotated.png]

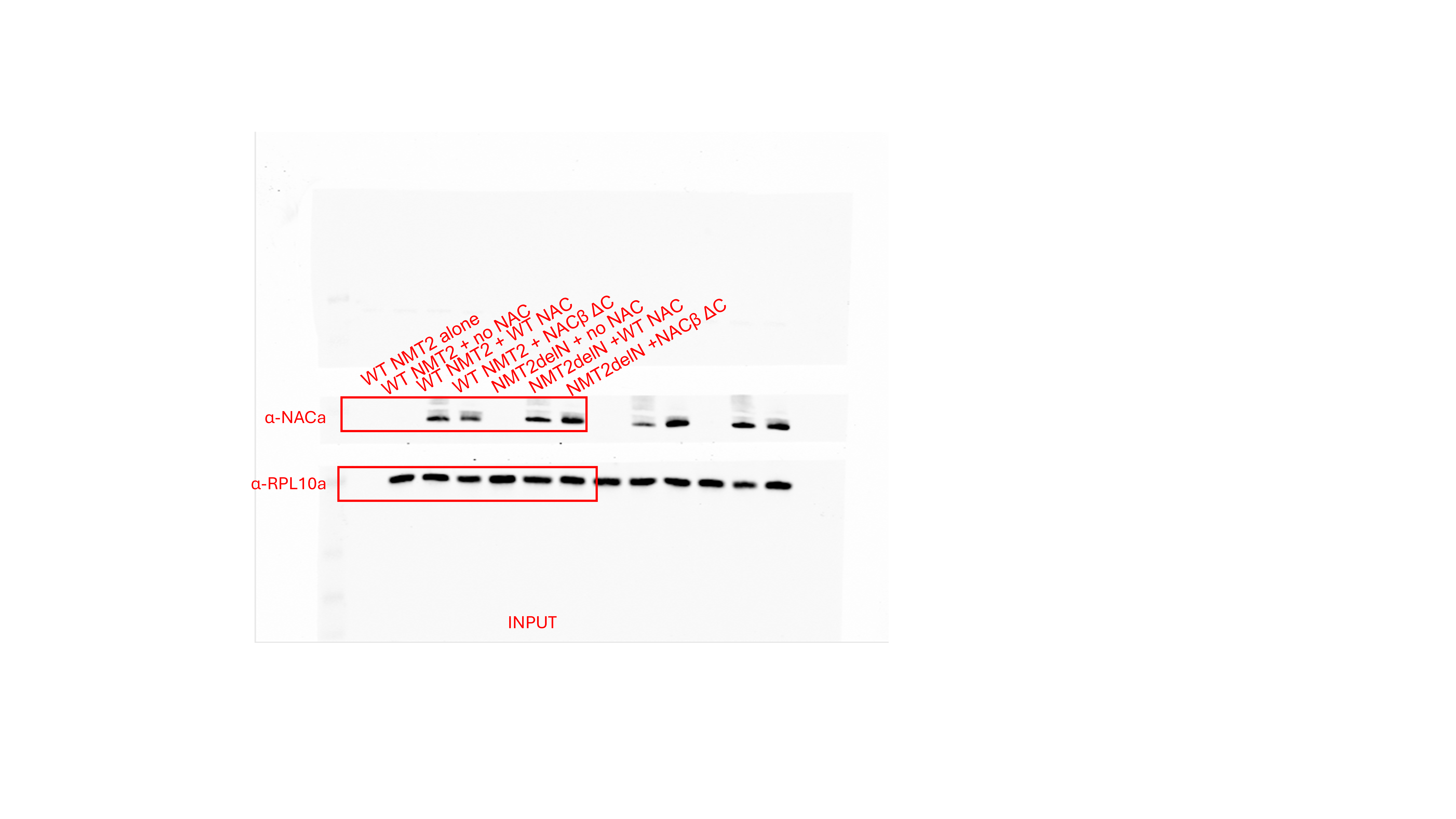

Supplement: Supplementary file 6 — Source data Fig. 6 [file 44318_2025_548_MOESM6_ESM.zip › EMBO-J-20205-120636_SourceDataFigure6/Panel E/Panel E input WB 800 annotated.png]

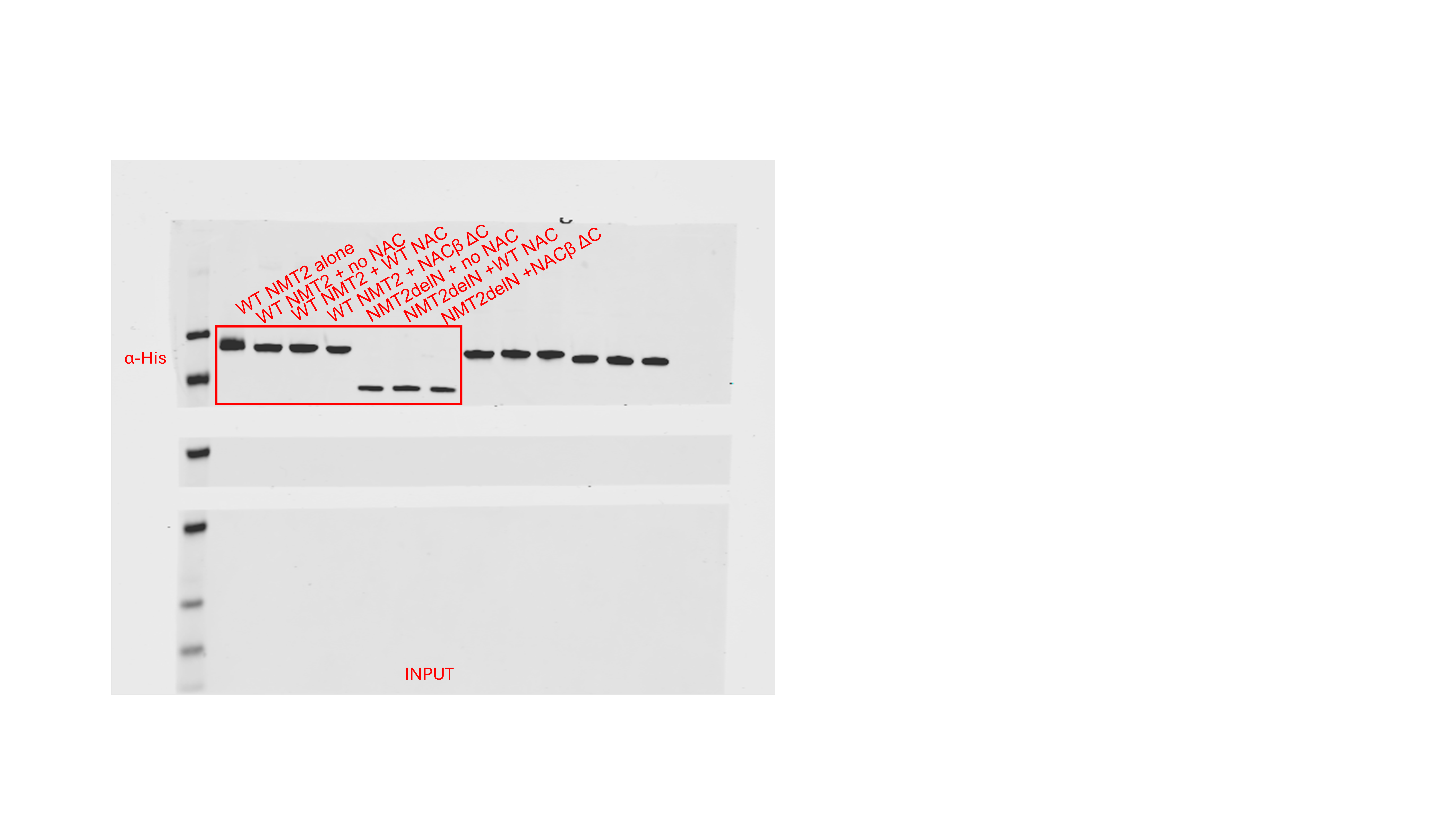

Supplement: Supplementary file 6 — Source data Fig. 6 [file 44318_2025_548_MOESM6_ESM.zip › EMBO-J-20205-120636_SourceDataFigure6/Panel E/Panel E input WB 700 annotated.png]

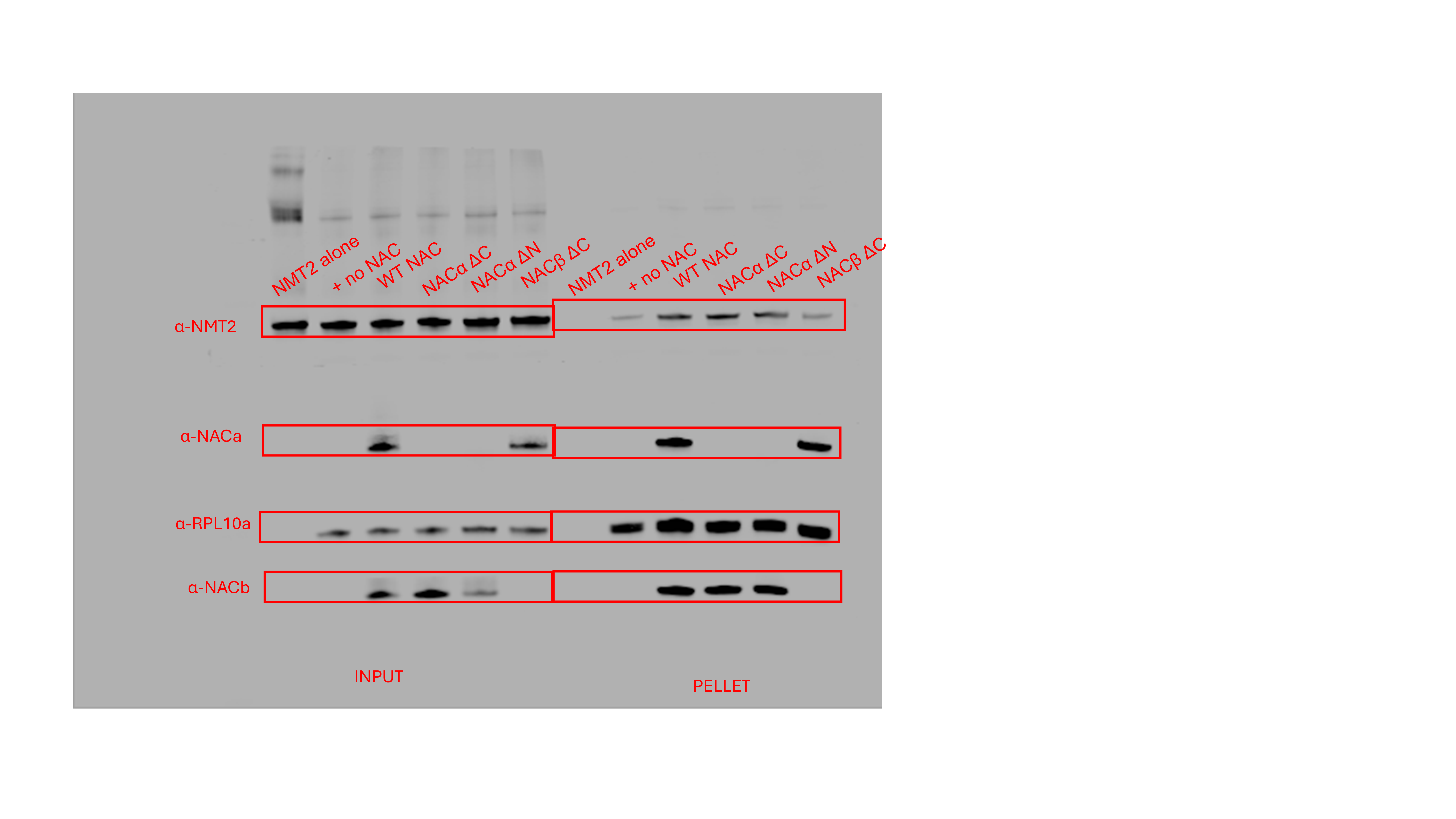

Supplement: Supplementary file 6 — Source data Fig. 6 [file 44318_2025_548_MOESM6_ESM.zip › EMBO-J-20205-120636_SourceDataFigure6/Panel C/Panel C input + pellet WB annotated.png]

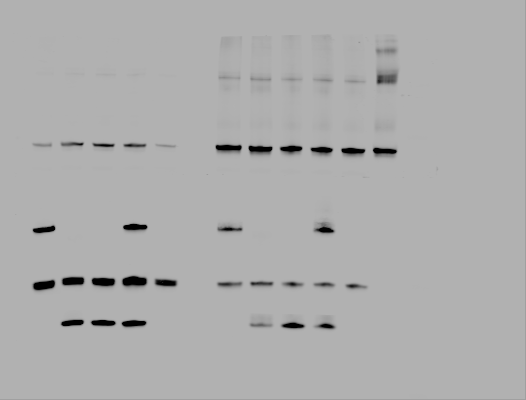

Supplement: Supplementary file 6 — Source data Fig. 6 [file 44318_2025_548_MOESM6_ESM.zip › EMBO-J-20205-120636_SourceDataFigure6/Panel C/42225-tailcosed-input and pellet.tif]

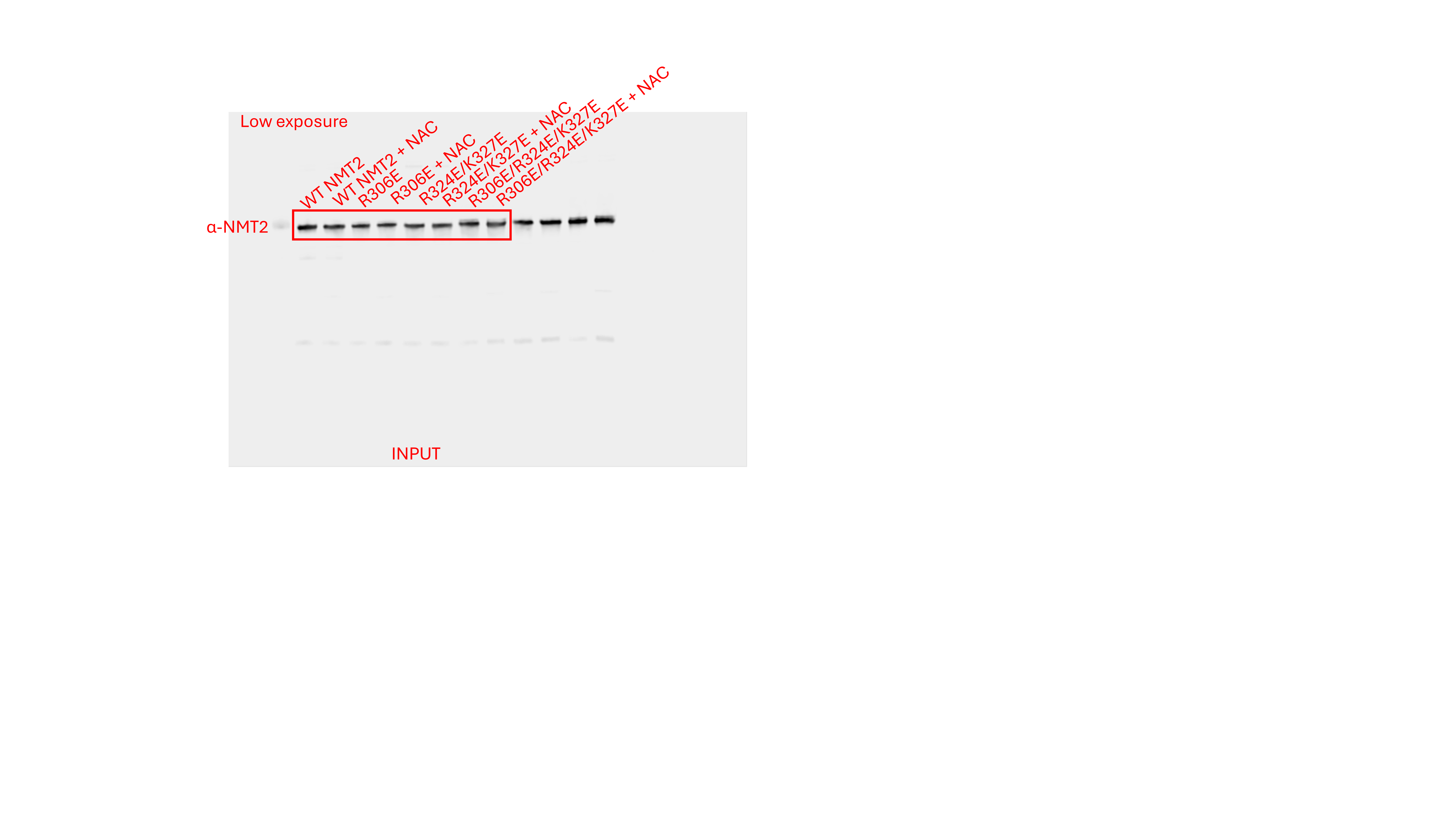

Supplement: Supplementary file 7 — Appendix Source Data [file 44318_2025_548_MOESM7_ESM.zip › EMBO-J-20205-120636_SourceDataForAppendix/Appendix S2/Panel B/Panel B input WB low exposure annotated.png]

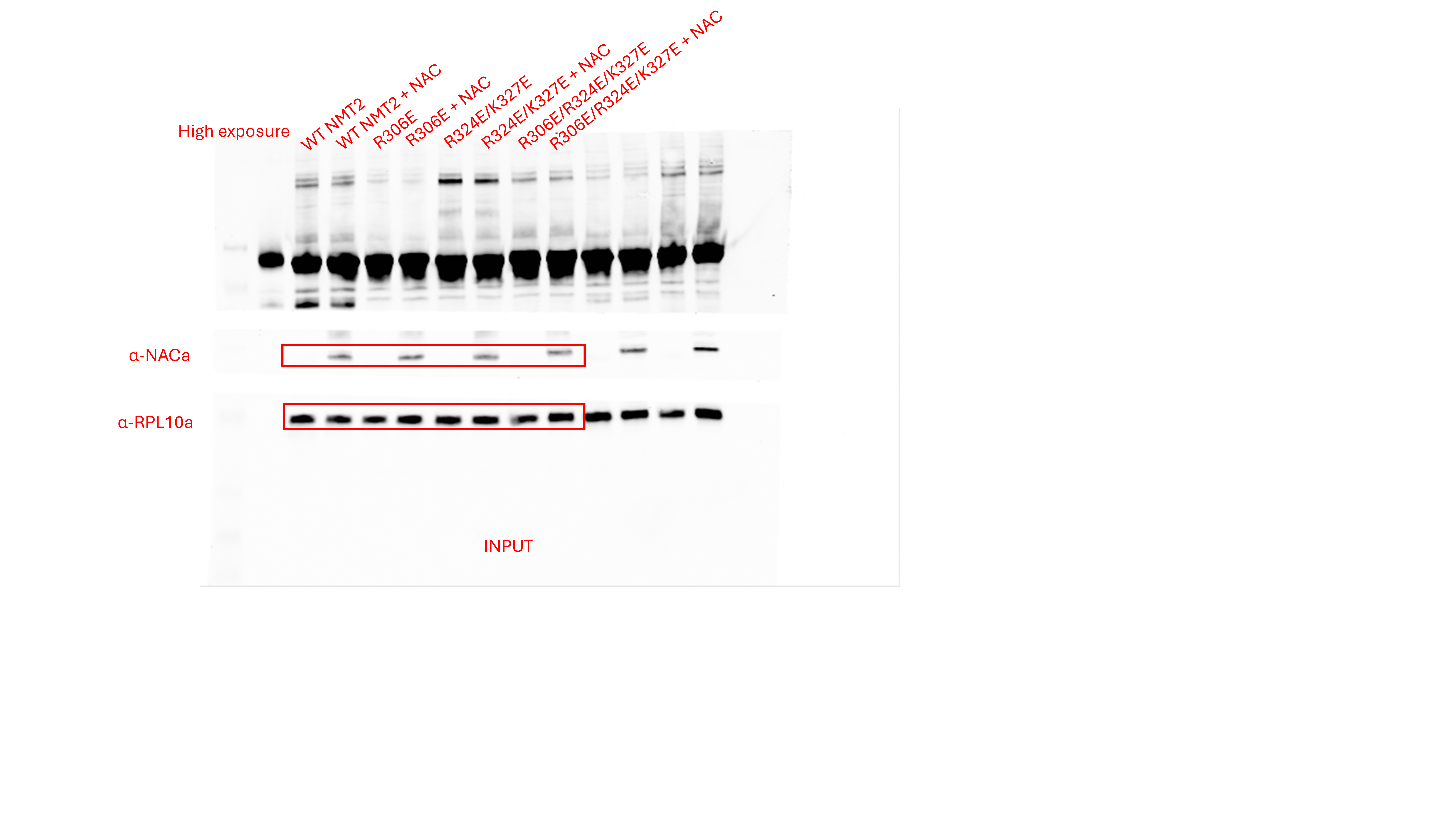

Supplement: Supplementary file 7 — Appendix Source Data [file 44318_2025_548_MOESM7_ESM.zip › EMBO-J-20205-120636_SourceDataForAppendix/Appendix S2/Panel B/Panel B inpute WB high exposure annotated.png]

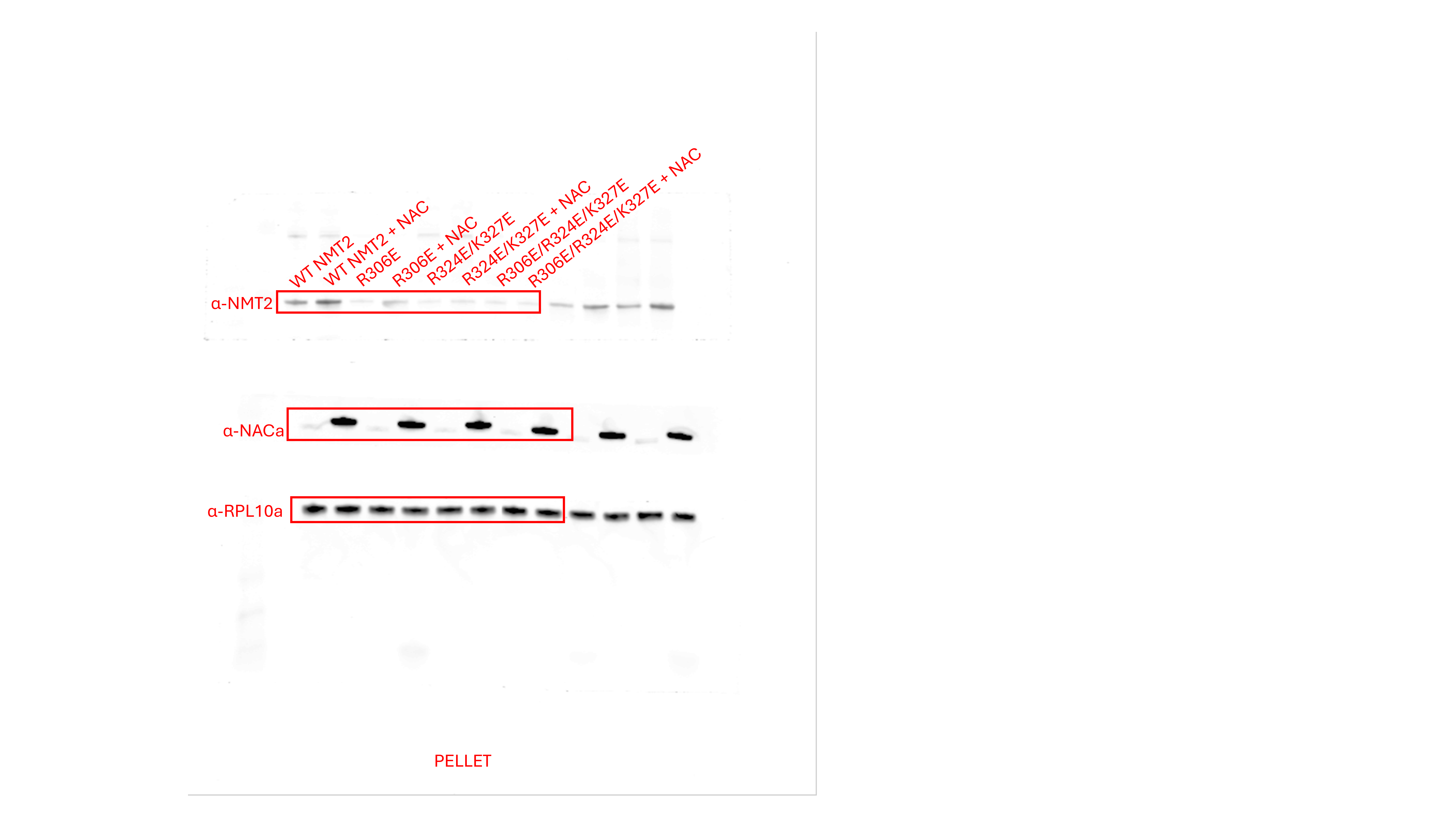

Supplement: Supplementary file 7 — Appendix Source Data [file 44318_2025_548_MOESM7_ESM.zip › EMBO-J-20205-120636_SourceDataForAppendix/Appendix S2/Panel B/Panel B pellet WB annotated.png]

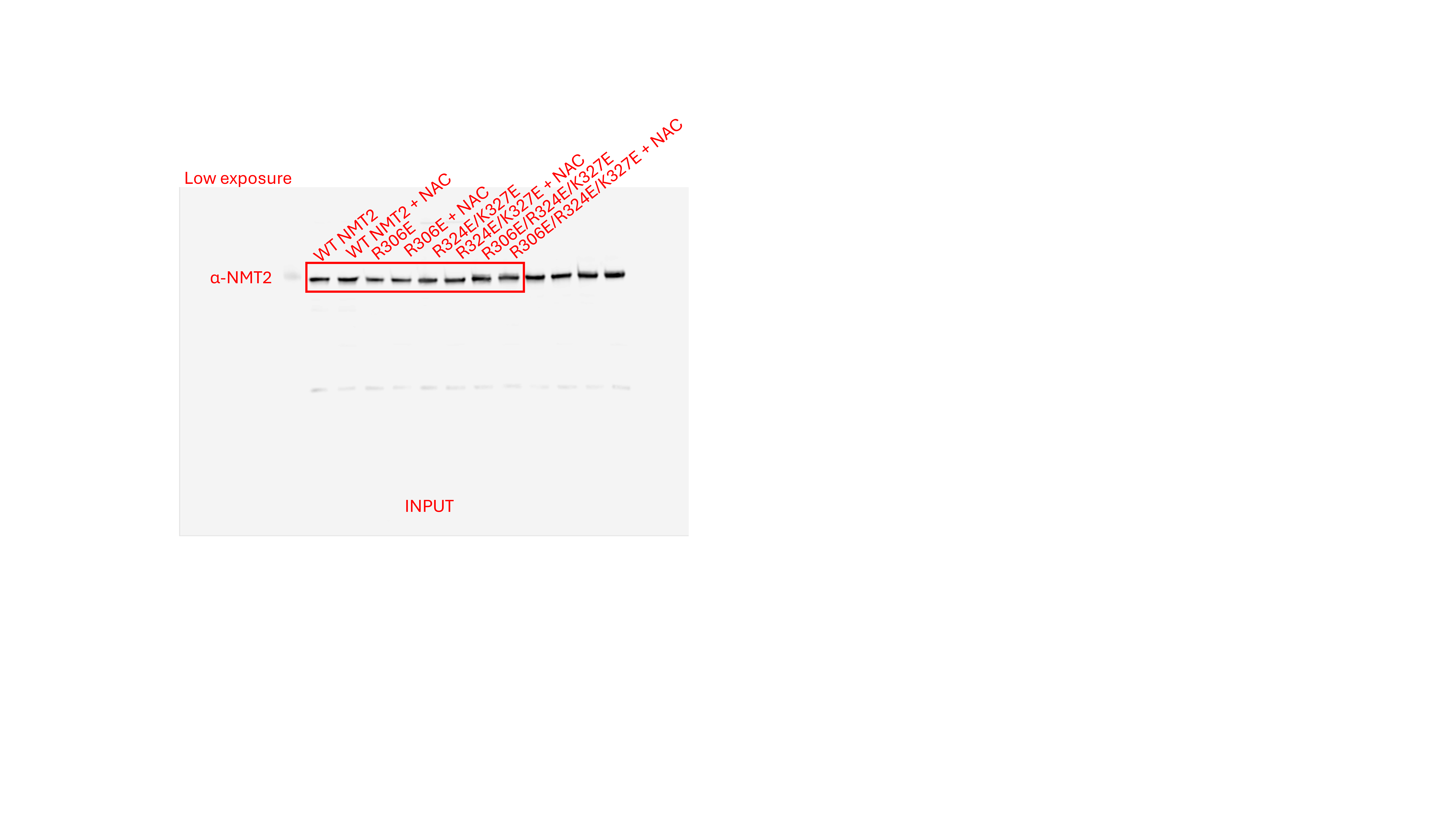

Supplement: Supplementary file 7 — Appendix Source Data [file 44318_2025_548_MOESM7_ESM.zip › EMBO-J-20205-120636_SourceDataForAppendix/Appendix S2/Panel A/Panel A input WB low exposure annotated.png]

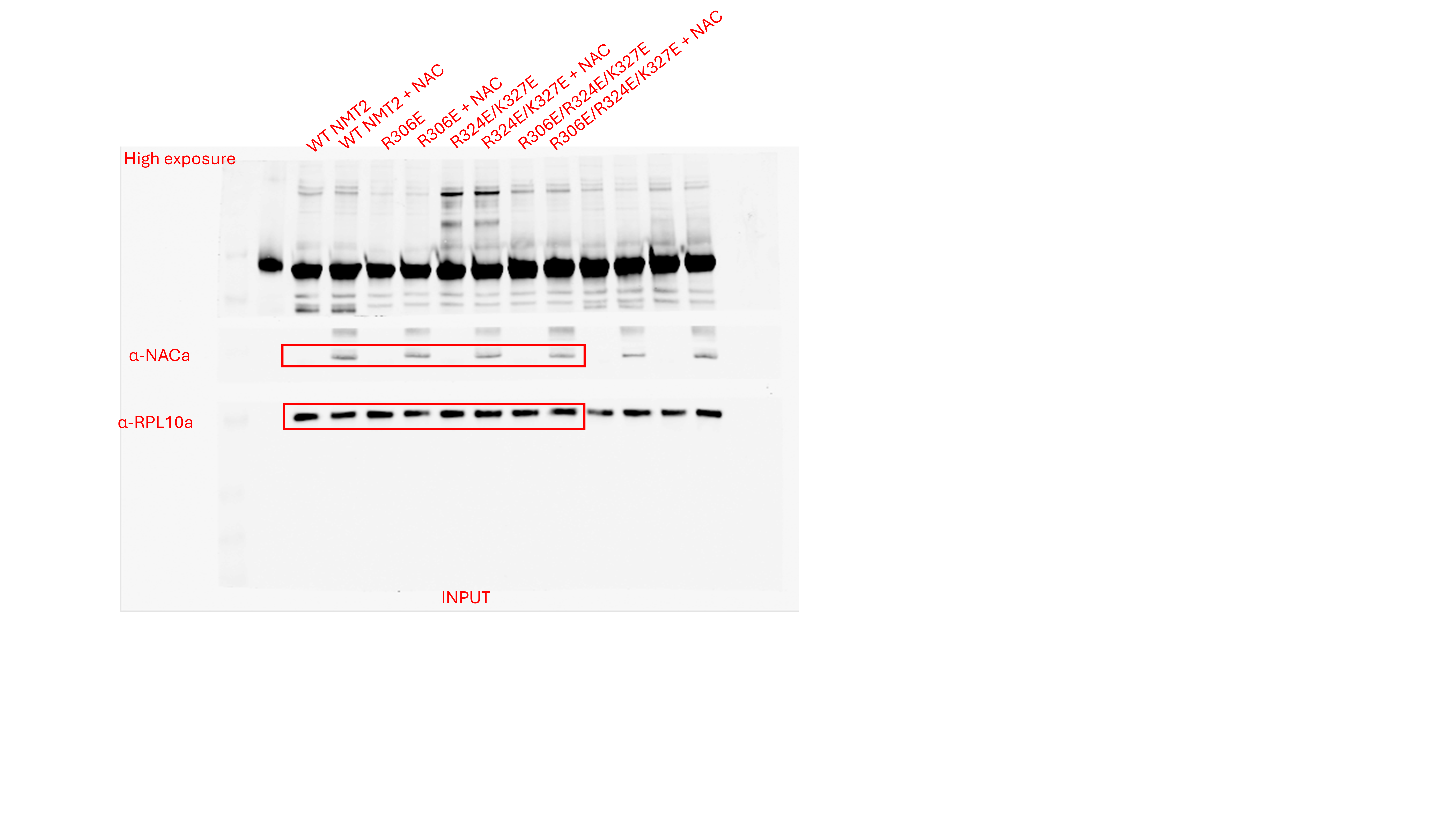

Supplement: Supplementary file 7 — Appendix Source Data [file 44318_2025_548_MOESM7_ESM.zip › EMBO-J-20205-120636_SourceDataForAppendix/Appendix S2/Panel A/Panel A input WB high exposure annotated.png]

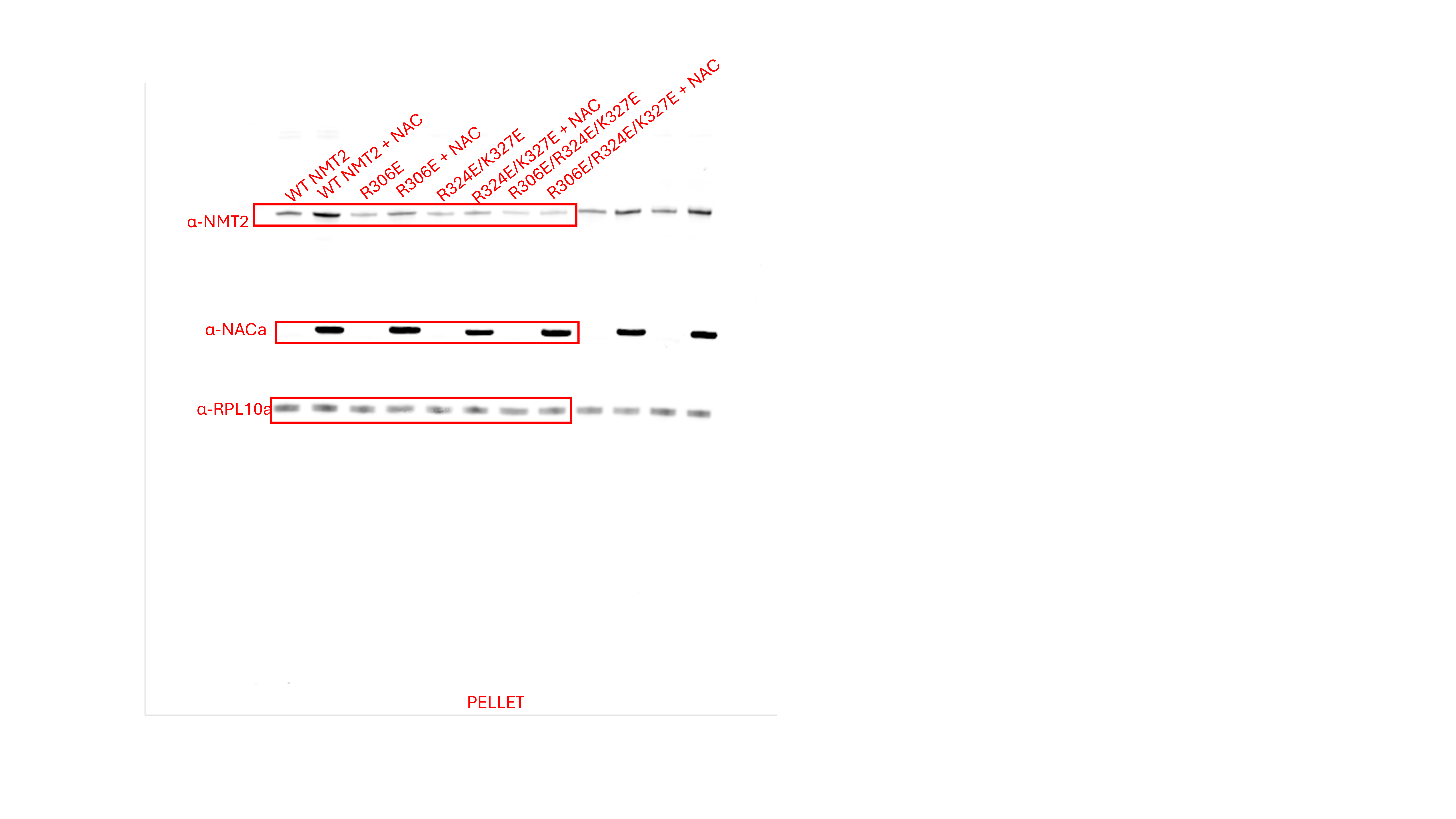

Supplement: Supplementary file 7 — Appendix Source Data [file 44318_2025_548_MOESM7_ESM.zip › EMBO-J-20205-120636_SourceDataForAppendix/Appendix S2/Panel A/Panel A pellet WB annotated.png]

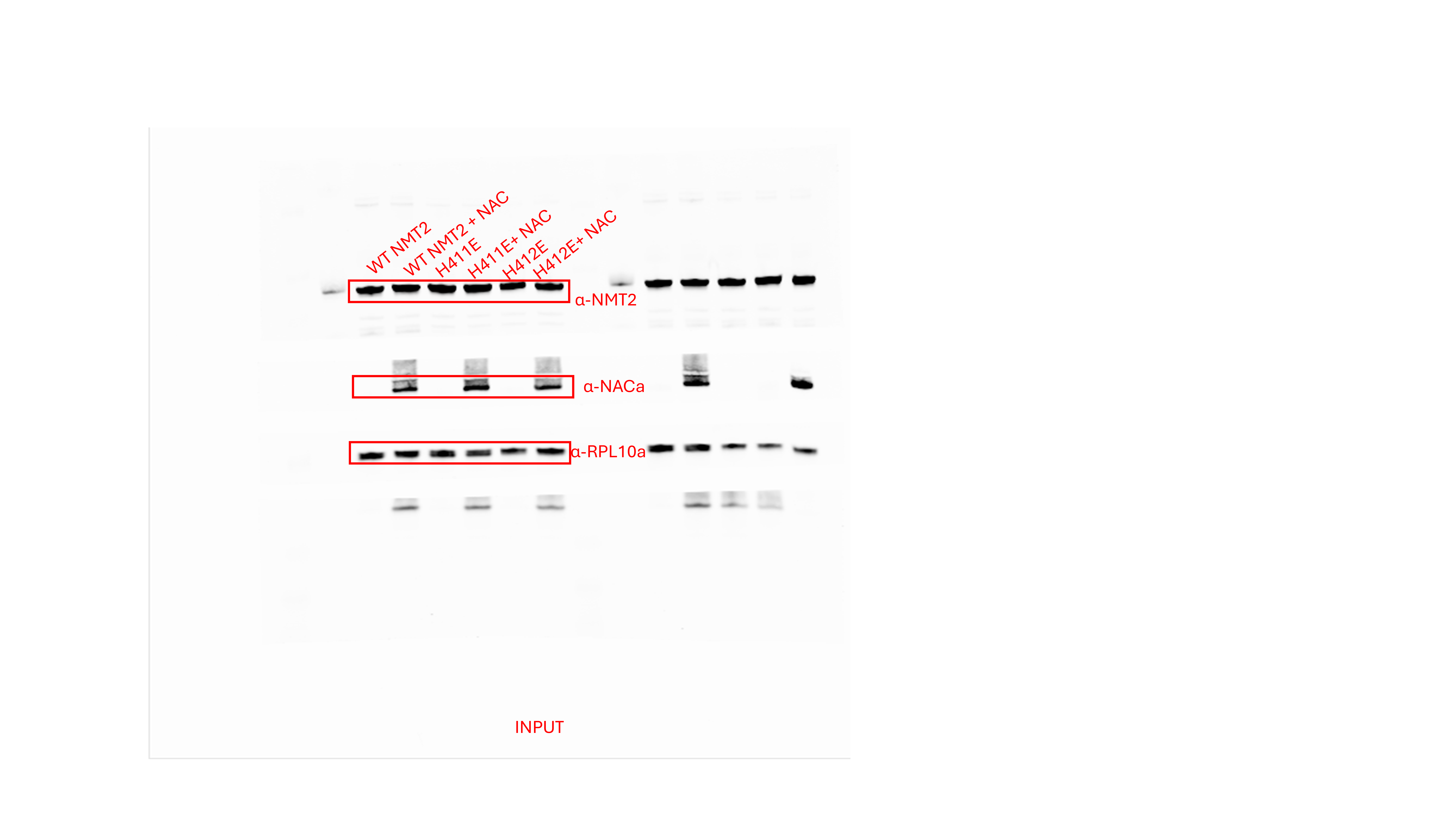

Supplement: Supplementary file 7 — Appendix Source Data [file 44318_2025_548_MOESM7_ESM.zip › EMBO-J-20205-120636_SourceDataForAppendix/Appendix S3/Panel B/Panel B input WB annotated.png]

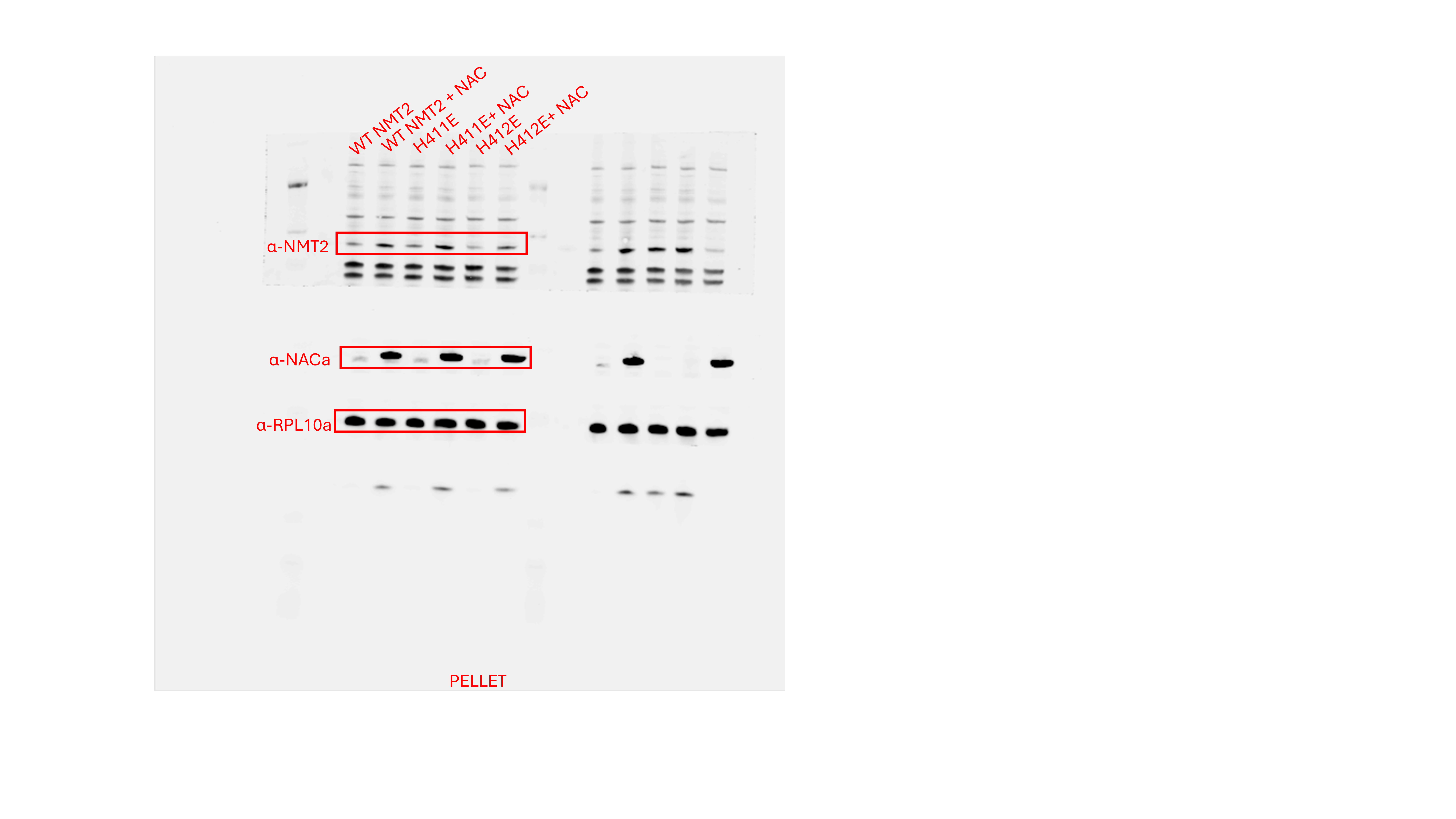

Supplement: Supplementary file 7 — Appendix Source Data [file 44318_2025_548_MOESM7_ESM.zip › EMBO-J-20205-120636_SourceDataForAppendix/Appendix S3/Panel B/Panel B pellet WB annotated.png]

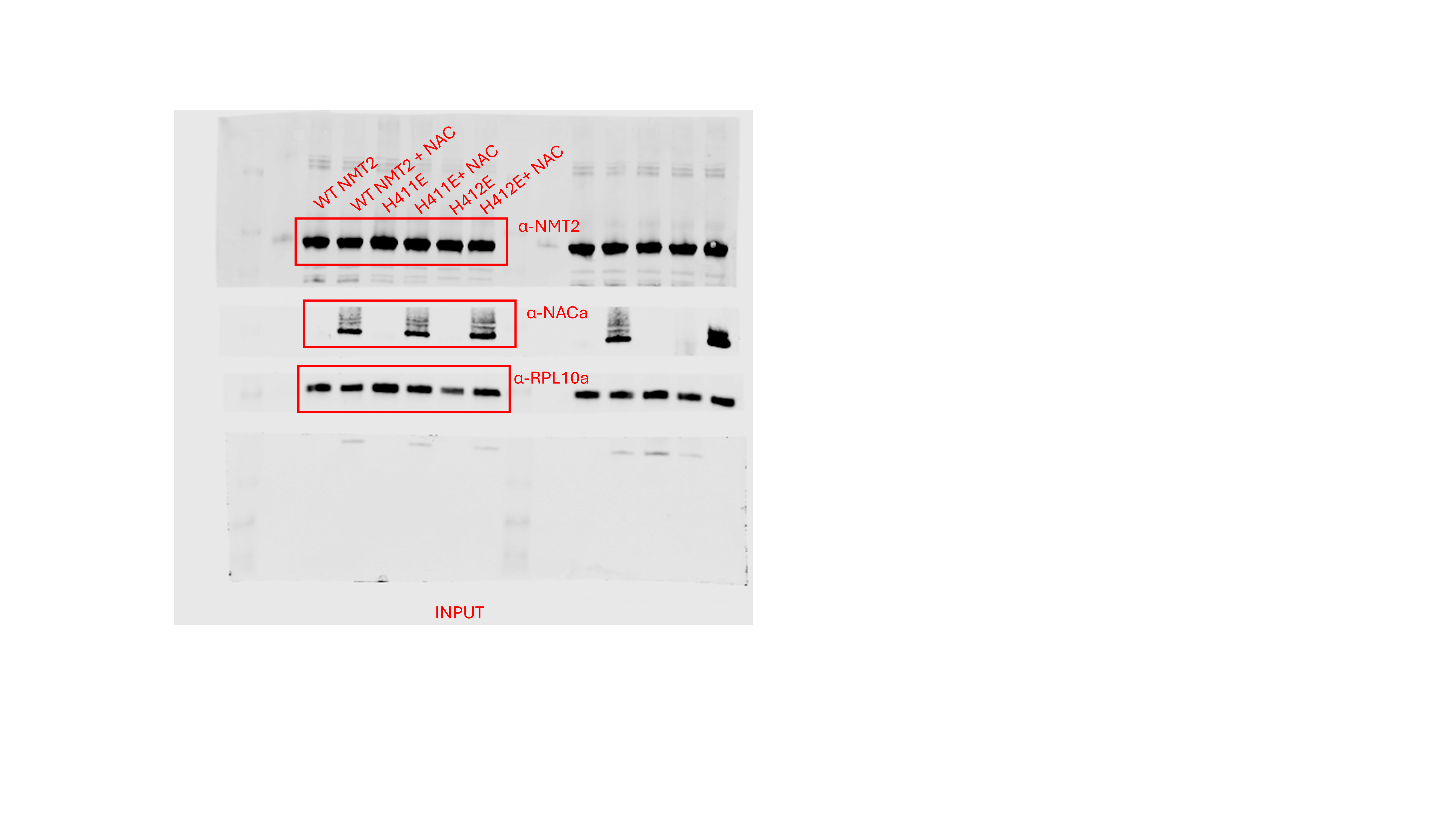

Supplement: Supplementary file 7 — Appendix Source Data [file 44318_2025_548_MOESM7_ESM.zip › EMBO-J-20205-120636_SourceDataForAppendix/Appendix S3/Panel A/Panel A input WB annotated.png]

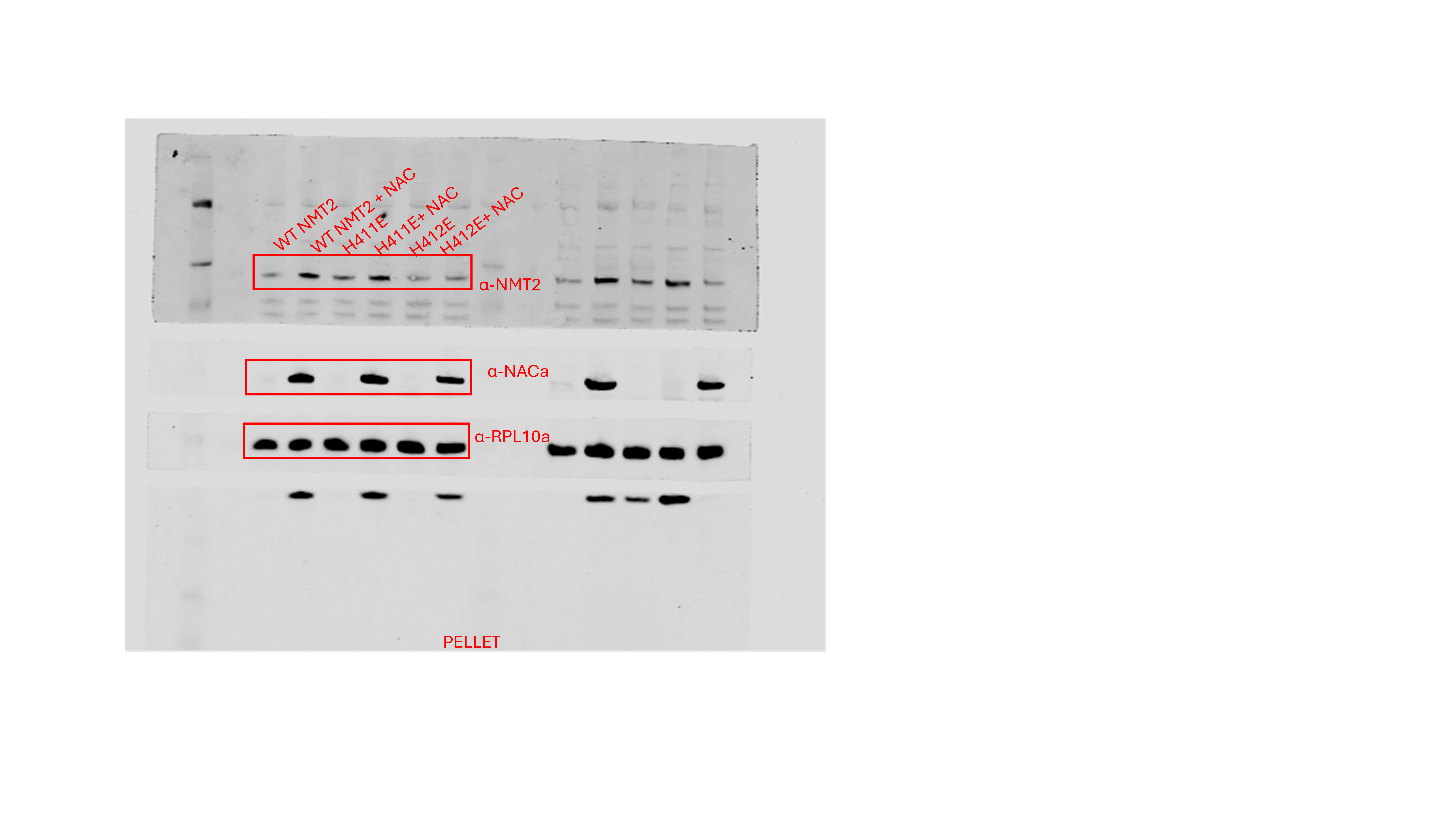

Supplement: Supplementary file 7 — Appendix Source Data [file 44318_2025_548_MOESM7_ESM.zip › EMBO-J-20205-120636_SourceDataForAppendix/Appendix S3/Panel A/Panel A pellet WB annotated.png]

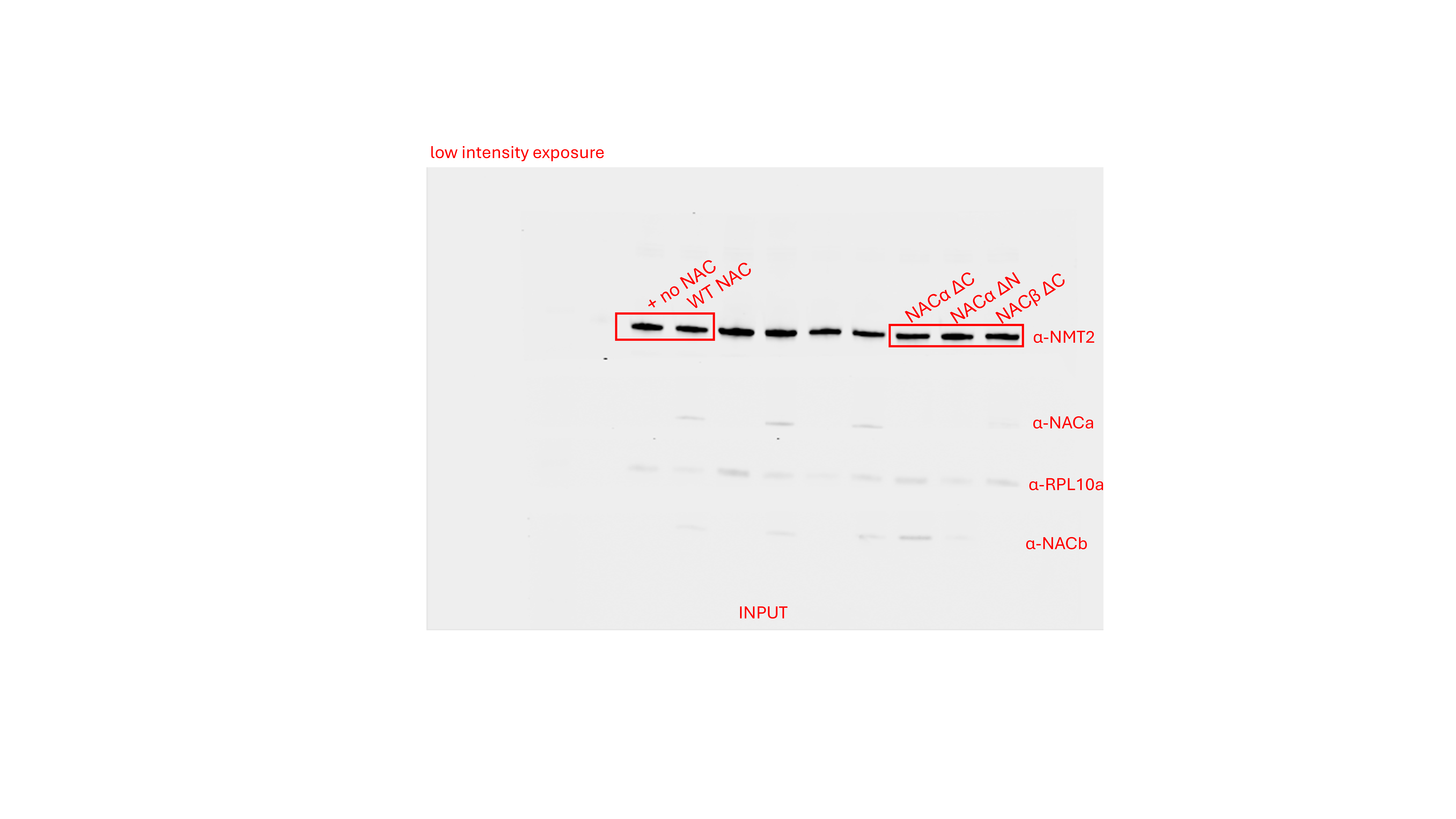

Supplement: Supplementary file 7 — Appendix Source Data [file 44318_2025_548_MOESM7_ESM.zip › EMBO-J-20205-120636_SourceDataForAppendix/Appendix S4/Panel B/Panel B input WB-annotated low exposure.png]

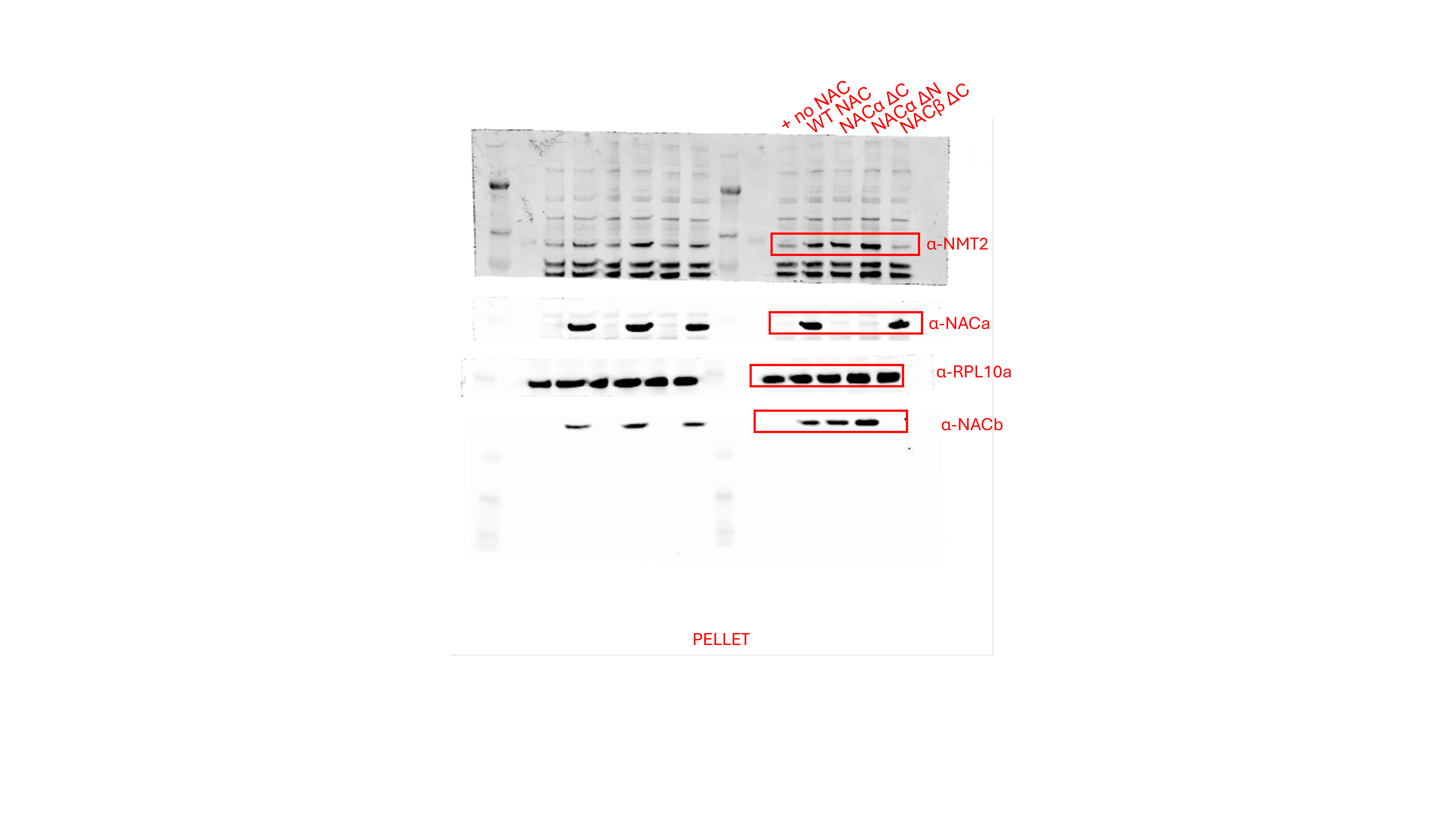

Supplement: Supplementary file 7 — Appendix Source Data [file 44318_2025_548_MOESM7_ESM.zip › EMBO-J-20205-120636_SourceDataForAppendix/Appendix S4/Panel B/Panel B pellet WB-annotated.png]

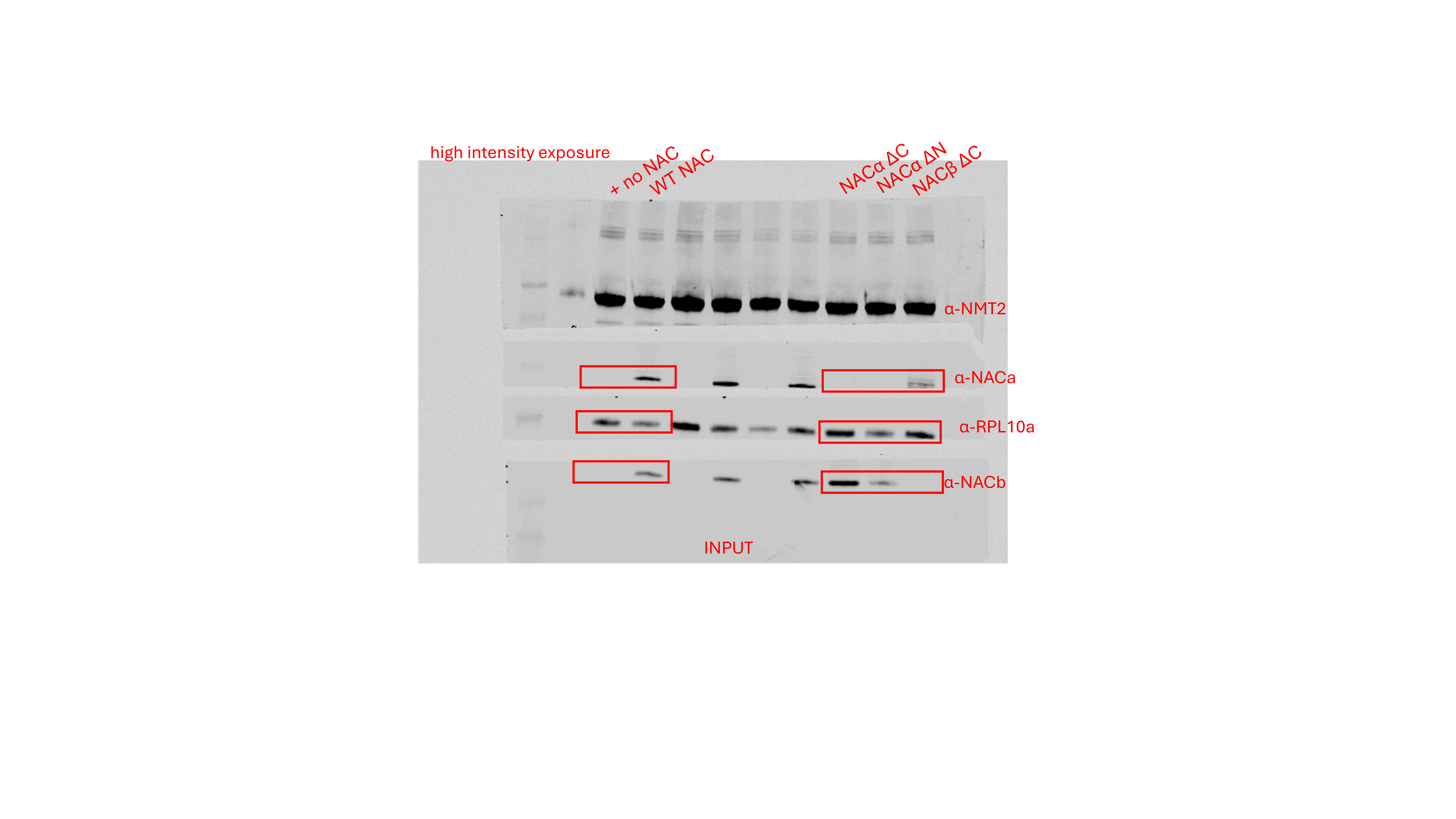

Supplement: Supplementary file 7 — Appendix Source Data [file 44318_2025_548_MOESM7_ESM.zip › EMBO-J-20205-120636_SourceDataForAppendix/Appendix S4/Panel B/Panel B- input WB annotated- high exposure.png]

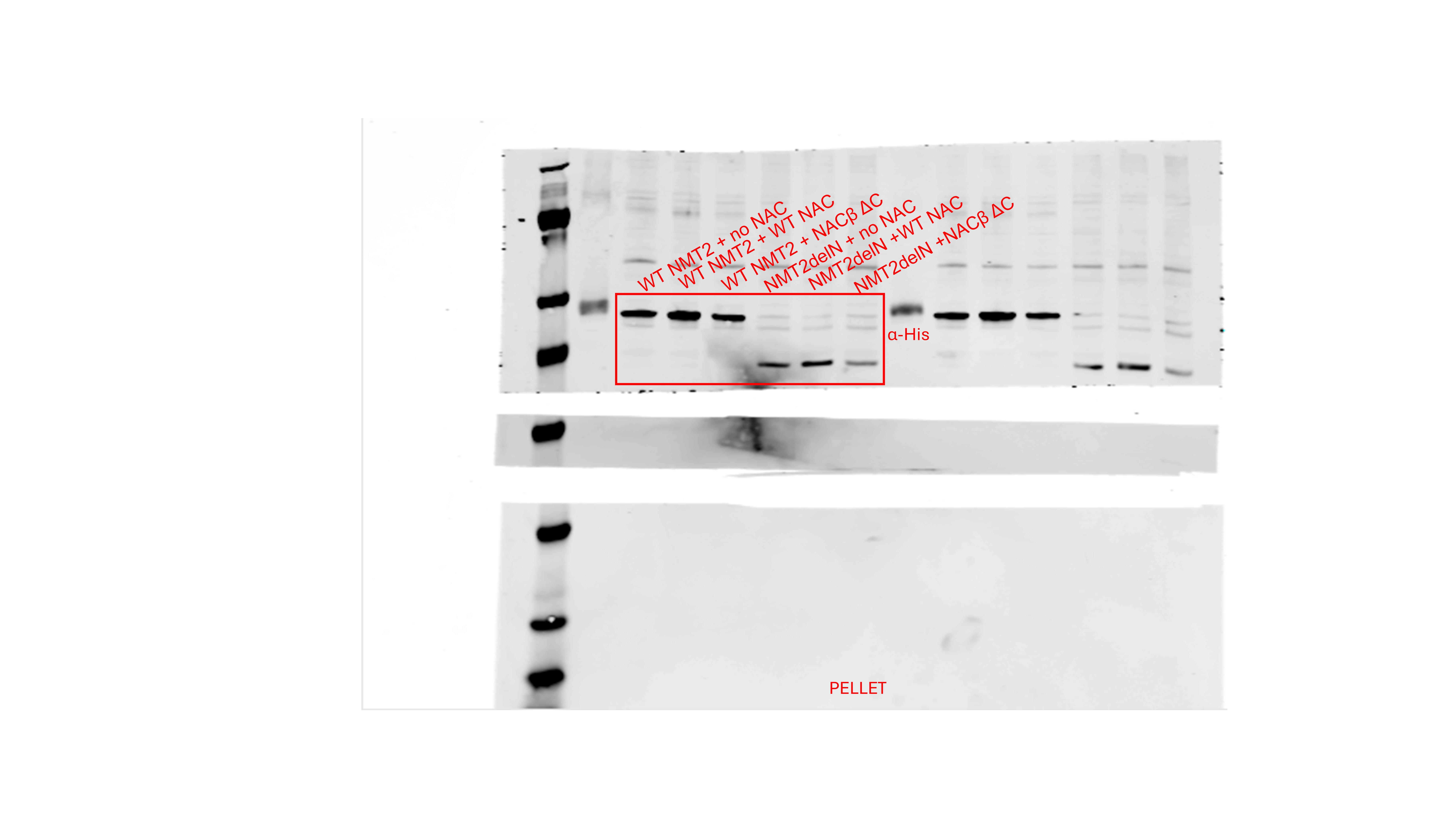

Supplement: Supplementary file 7 — Appendix Source Data [file 44318_2025_548_MOESM7_ESM.zip › EMBO-J-20205-120636_SourceDataForAppendix/Appendix S4/Panel E/Panel E pellet WB 700 annotated.png]

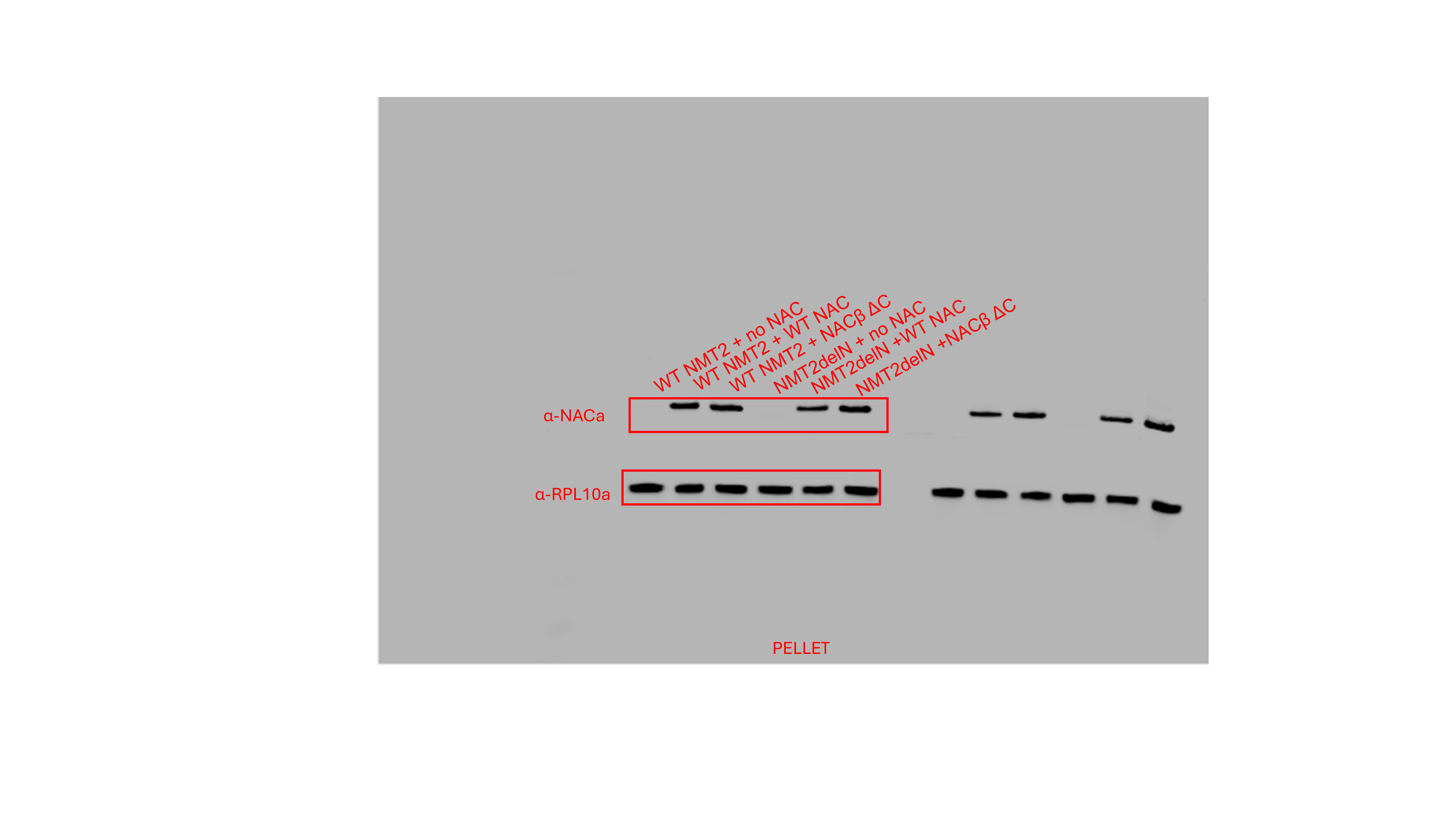

Supplement: Supplementary file 7 — Appendix Source Data [file 44318_2025_548_MOESM7_ESM.zip › EMBO-J-20205-120636_SourceDataForAppendix/Appendix S4/Panel E/Panel E pellet WB 800 annotated.png]

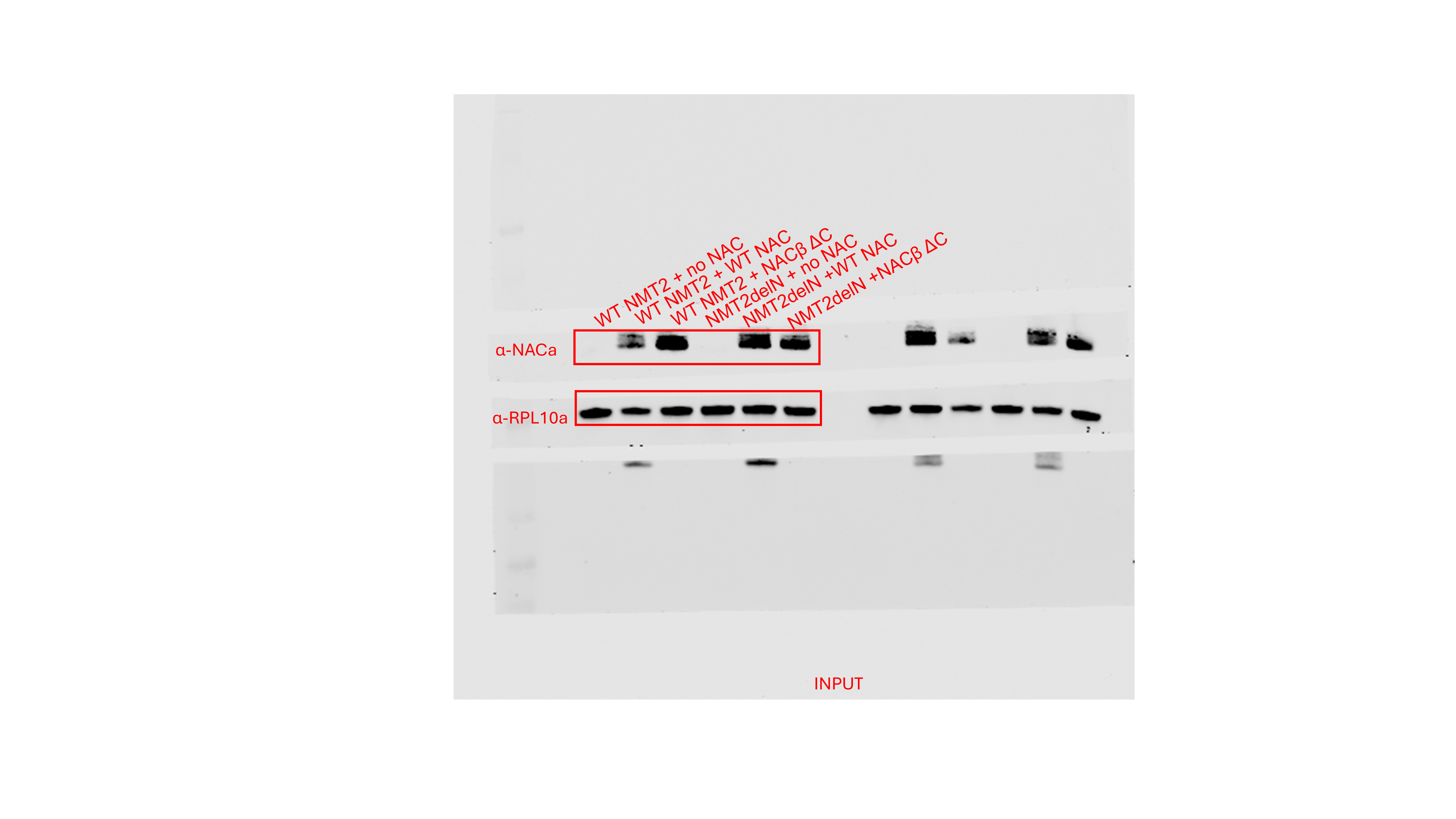

Supplement: Supplementary file 7 — Appendix Source Data [file 44318_2025_548_MOESM7_ESM.zip › EMBO-J-20205-120636_SourceDataForAppendix/Appendix S4/Panel E/Panel E input WB 800 annotated.png]

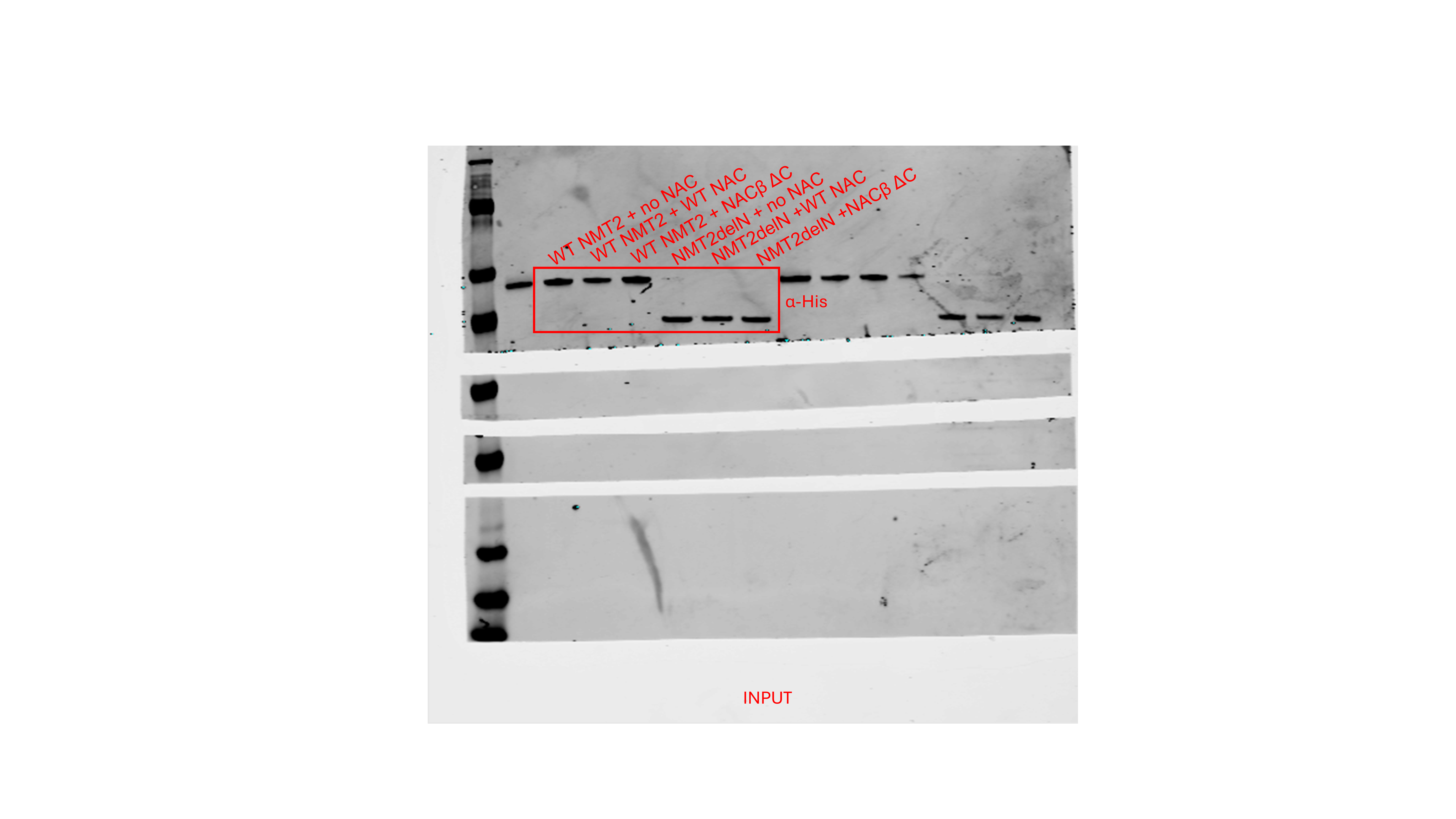

Supplement: Supplementary file 7 — Appendix Source Data [file 44318_2025_548_MOESM7_ESM.zip › EMBO-J-20205-120636_SourceDataForAppendix/Appendix S4/Panel E/Panel E input WB 700 annotated.png]

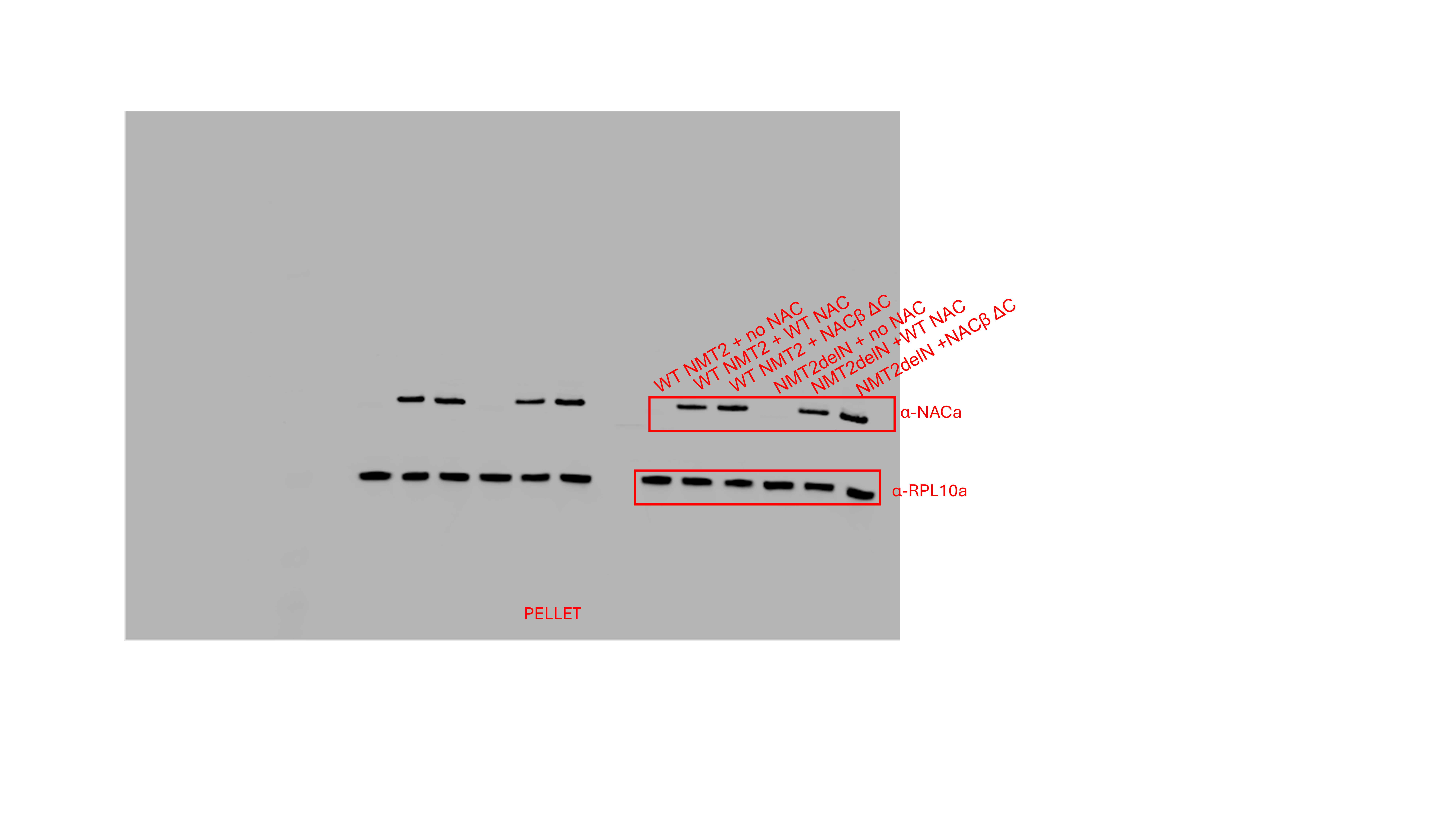

Supplement: Supplementary file 7 — Appendix Source Data [file 44318_2025_548_MOESM7_ESM.zip › EMBO-J-20205-120636_SourceDataForAppendix/Appendix S4/Panel D/Panel D pellet WB 800 annotated.png]

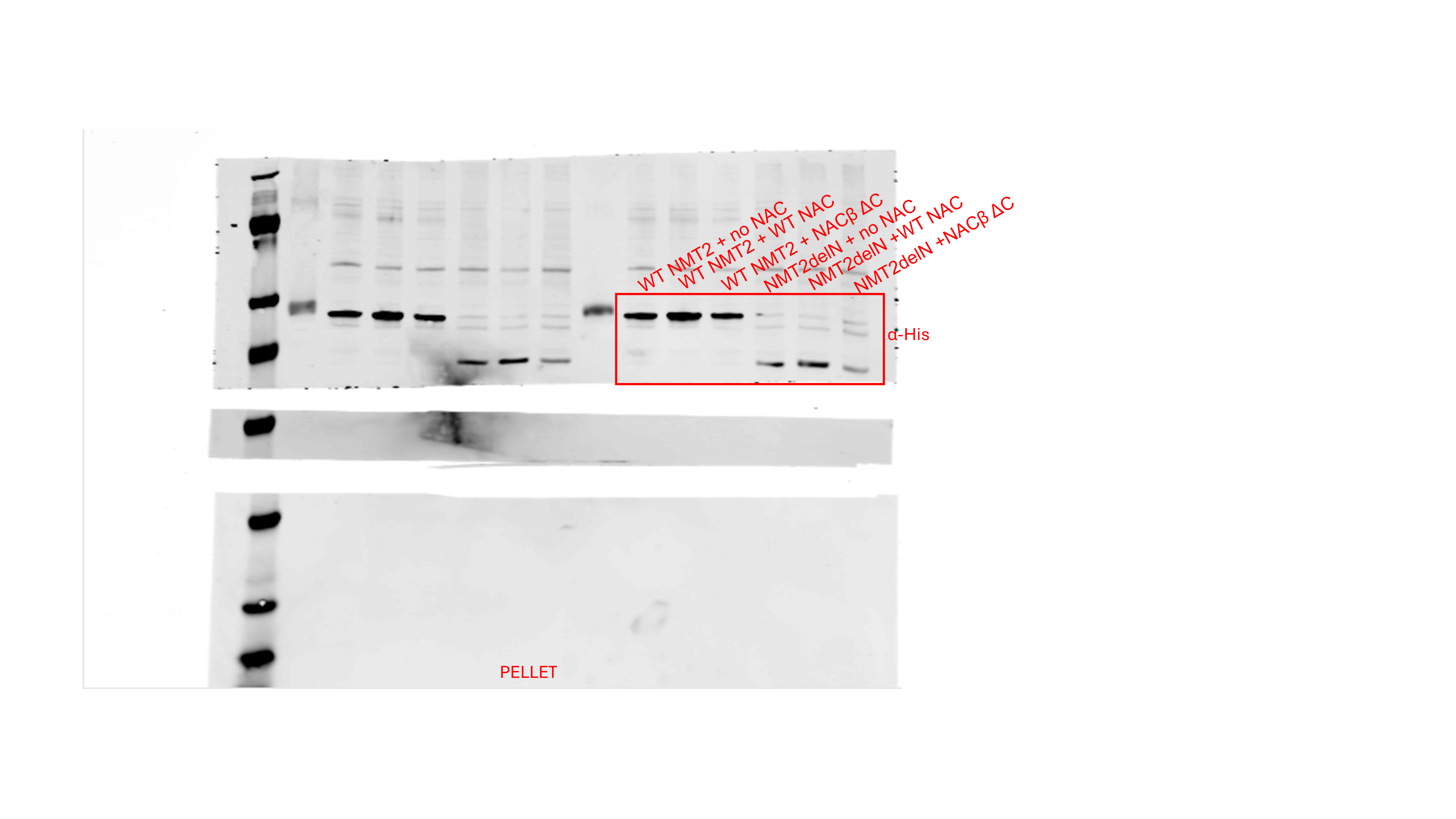

Supplement: Supplementary file 7 — Appendix Source Data [file 44318_2025_548_MOESM7_ESM.zip › EMBO-J-20205-120636_SourceDataForAppendix/Appendix S4/Panel D/Panel D pellet WB 700 annotated.png]

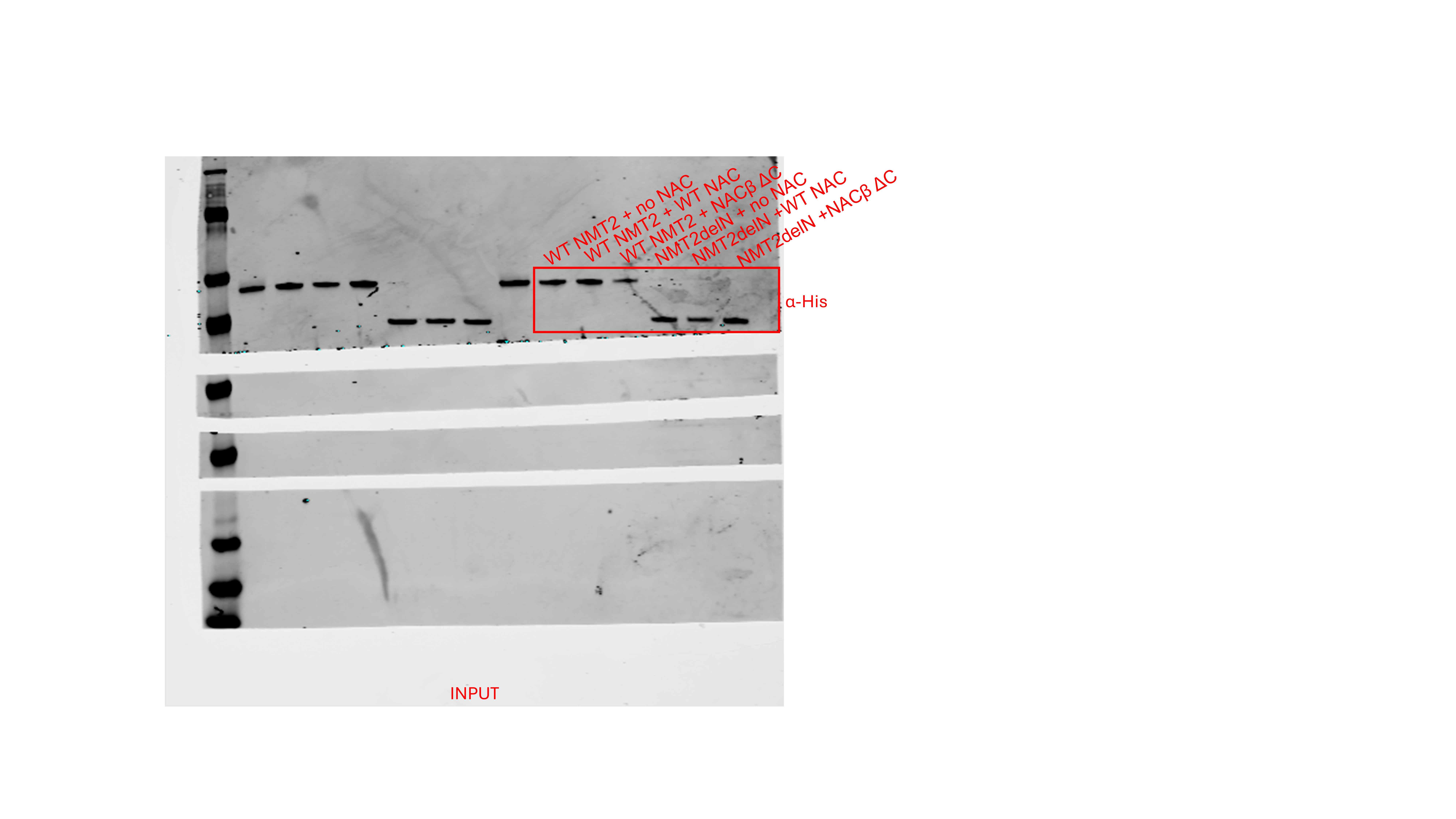

Supplement: Supplementary file 7 — Appendix Source Data [file 44318_2025_548_MOESM7_ESM.zip › EMBO-J-20205-120636_SourceDataForAppendix/Appendix S4/Panel D/Panel D input WB-700 annotated.png]

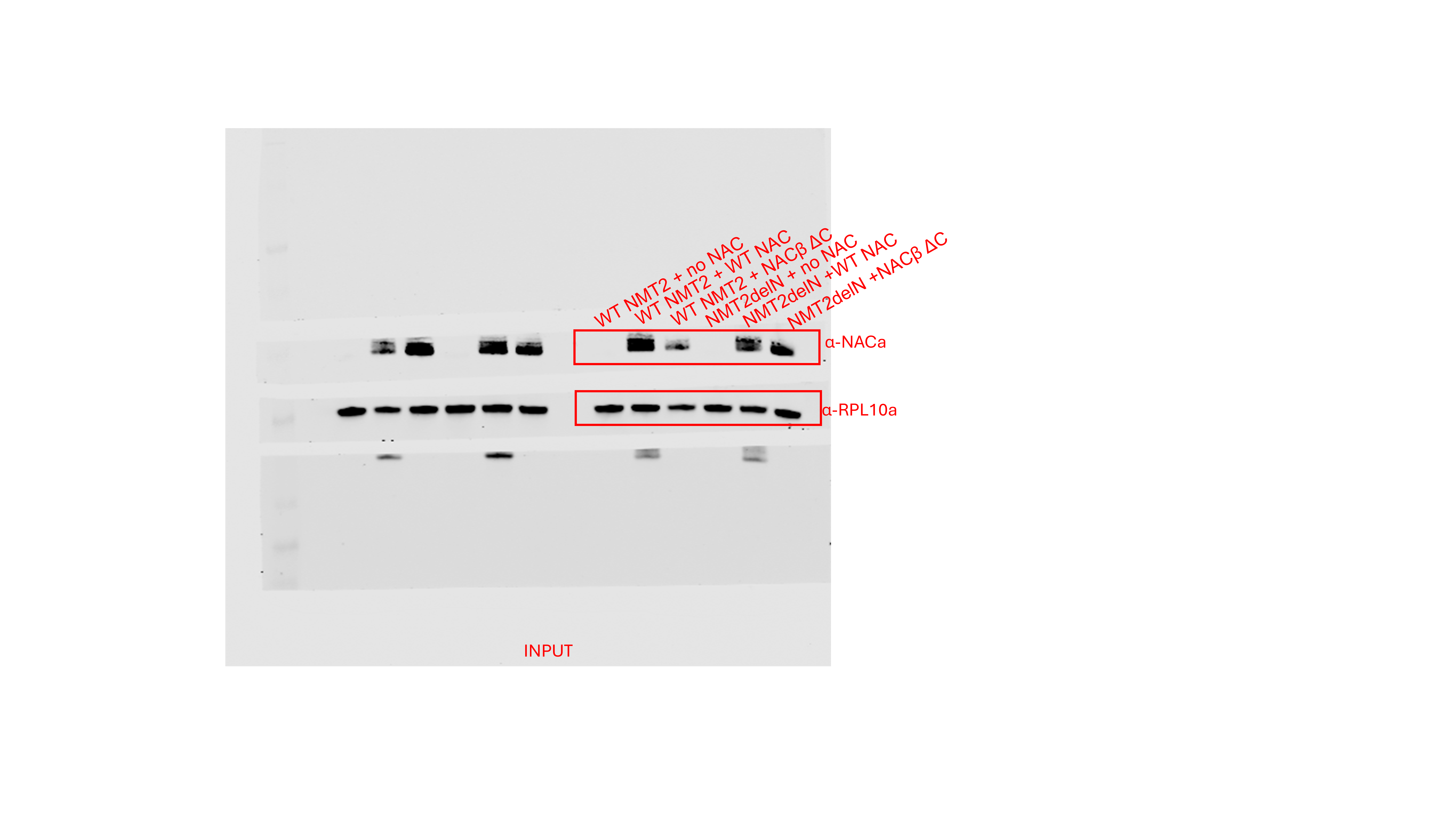

Supplement: Supplementary file 7 — Appendix Source Data [file 44318_2025_548_MOESM7_ESM.zip › EMBO-J-20205-120636_SourceDataForAppendix/Appendix S4/Panel D/Panel D input WB-800-annotated.png]

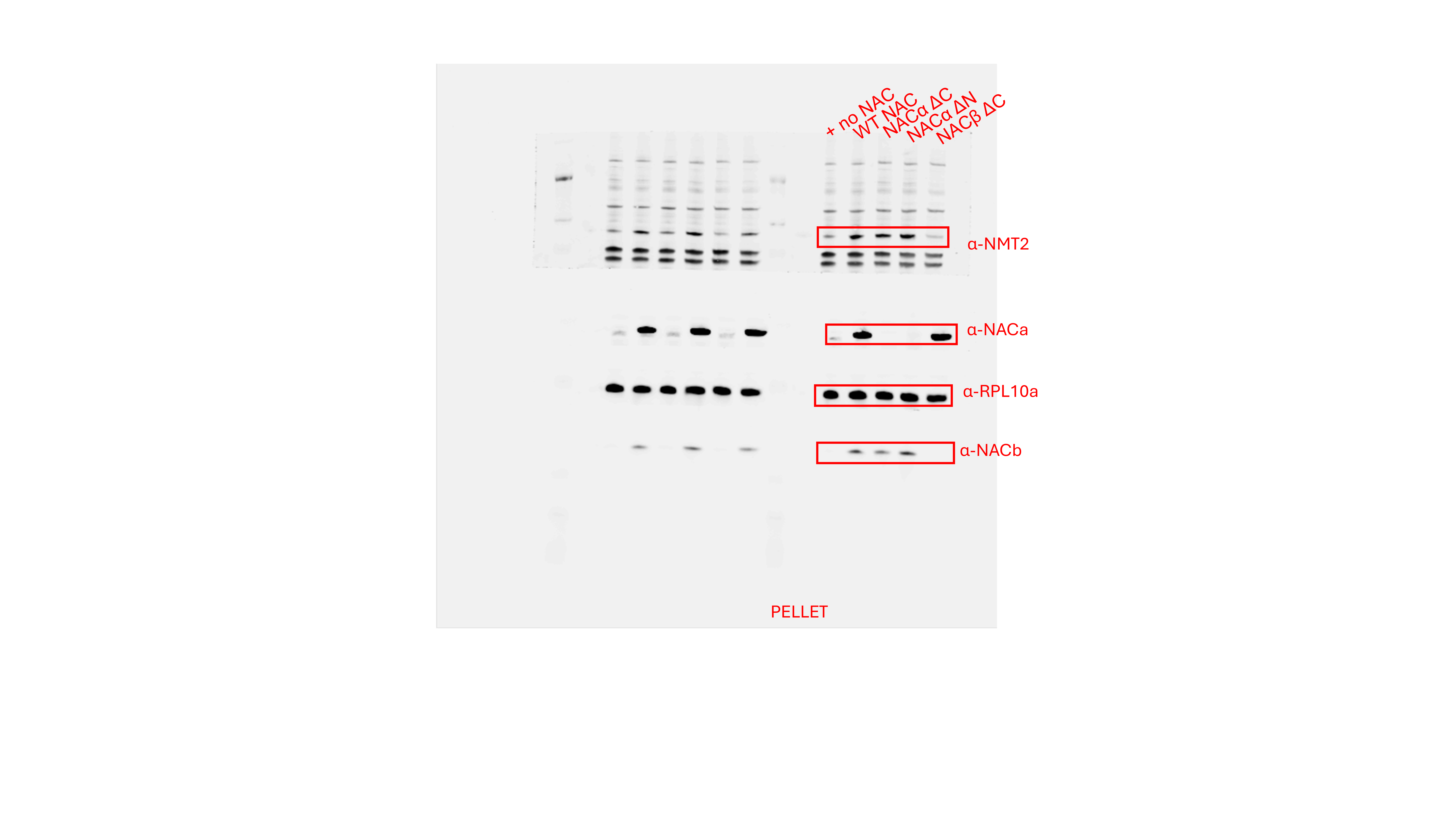

Supplement: Supplementary file 7 — Appendix Source Data [file 44318_2025_548_MOESM7_ESM.zip › EMBO-J-20205-120636_SourceDataForAppendix/Appendix S4/Panel C/Panel C pellet WB annotated.png]

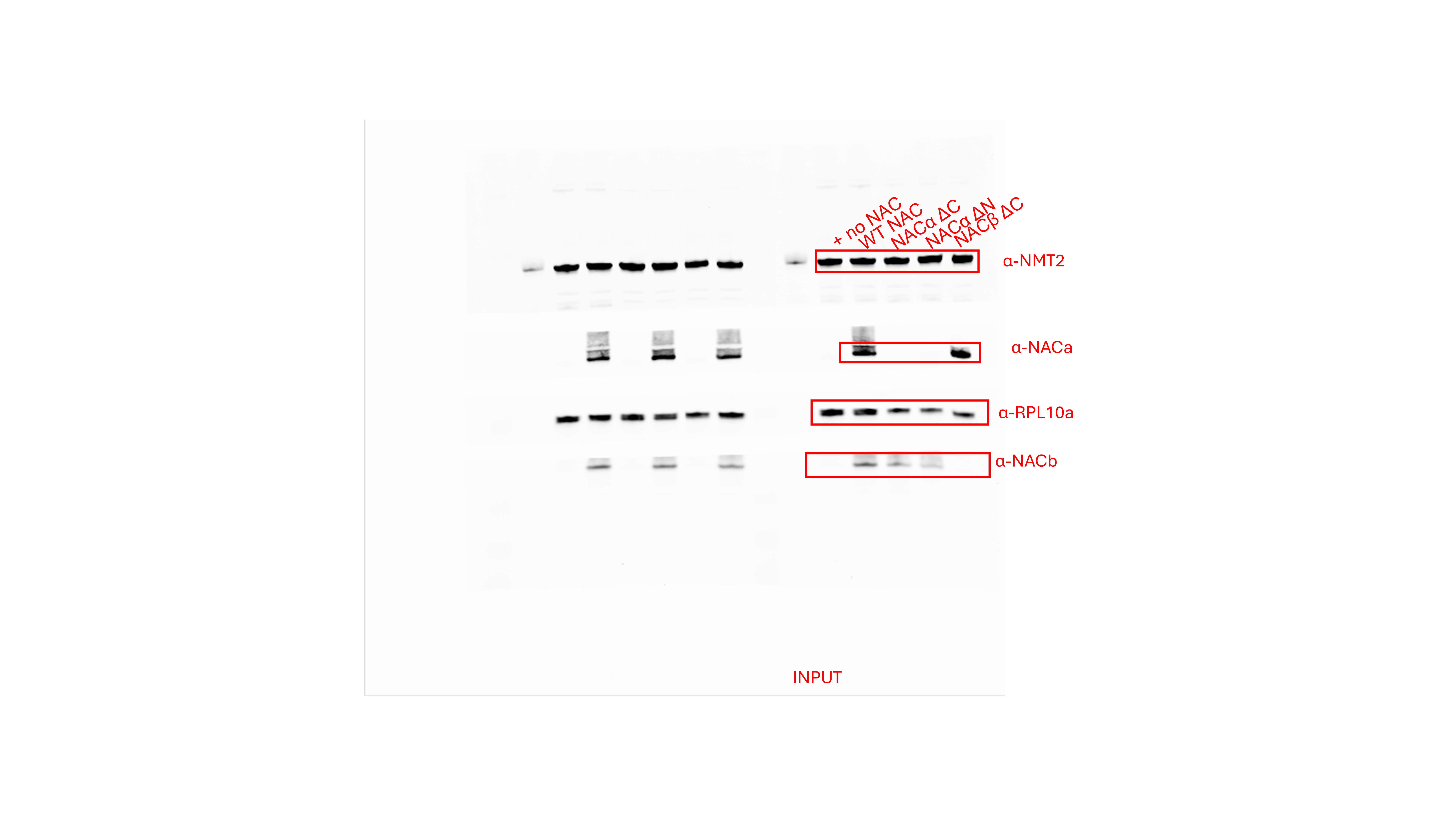

Supplement: Supplementary file 7 — Appendix Source Data [file 44318_2025_548_MOESM7_ESM.zip › EMBO-J-20205-120636_SourceDataForAppendix/Appendix S4/Panel C/Panel C input WB annotated.png]

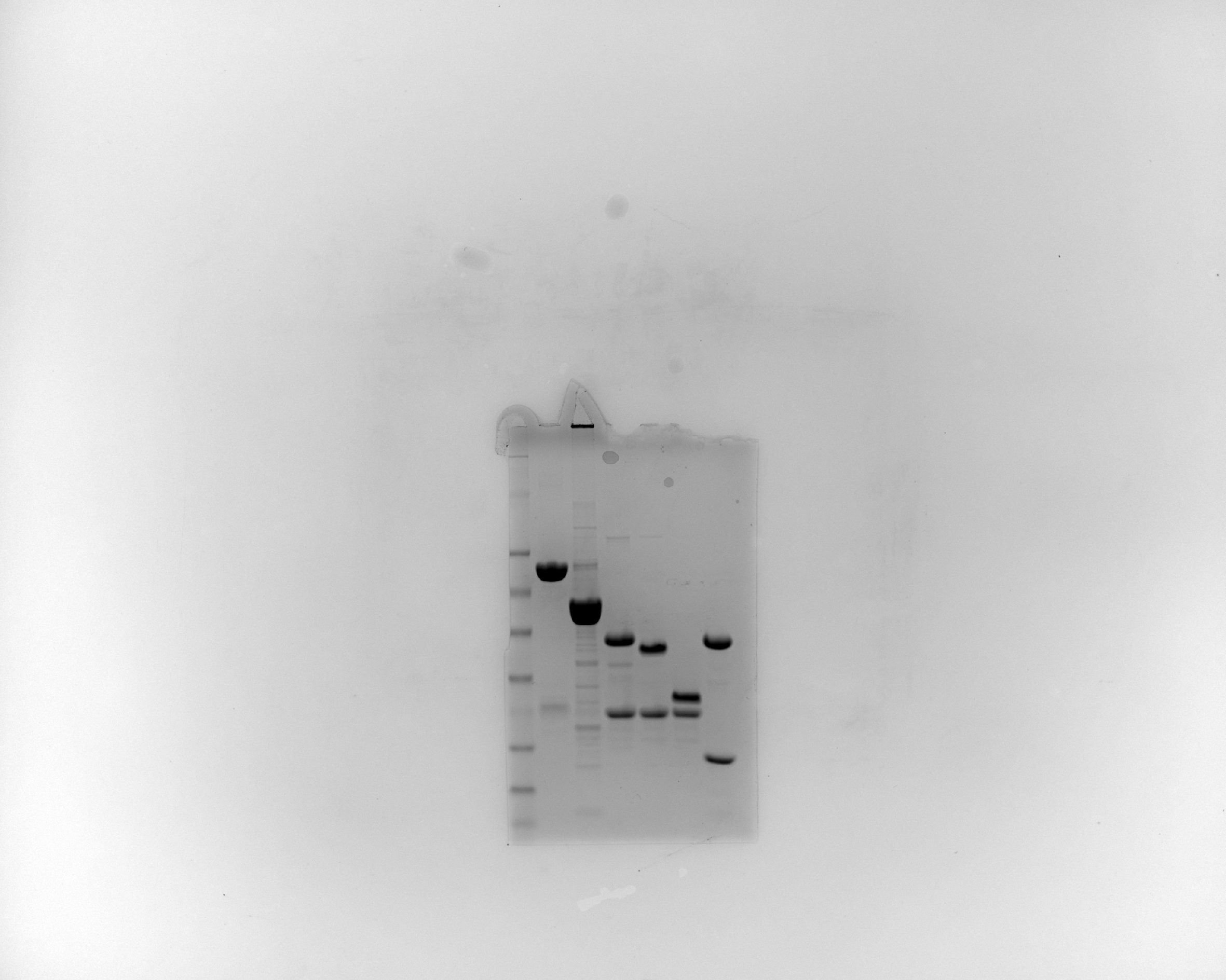

Supplement: Supplementary file 7 — Appendix Source Data [file 44318_2025_548_MOESM7_ESM.zip › EMBO-J-20205-120636_SourceDataForAppendix/Appendix S4/Panel A/panel A coomassie raw.tif]

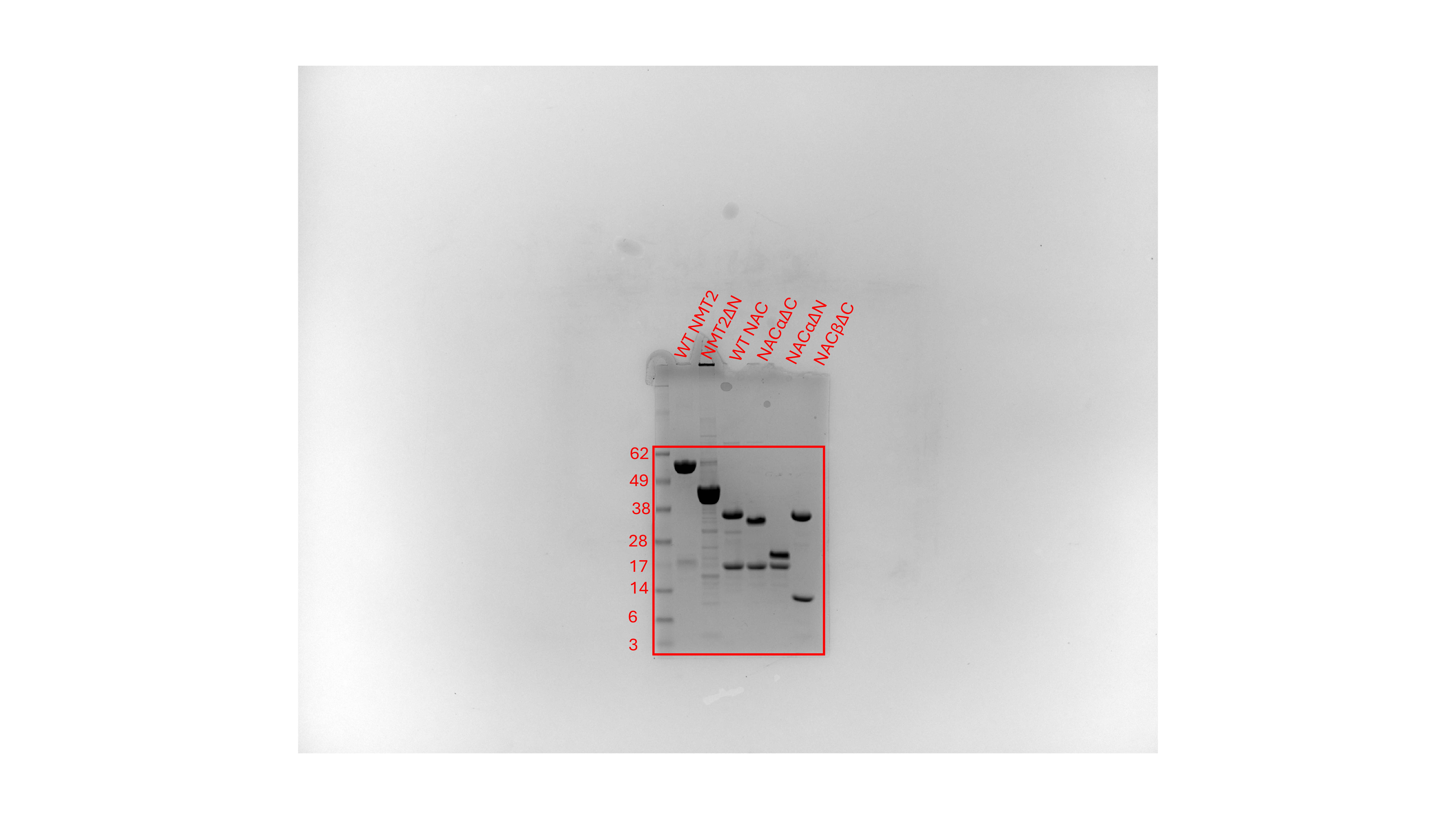

Supplement: Supplementary file 7 — Appendix Source Data [file 44318_2025_548_MOESM7_ESM.zip › EMBO-J-20205-120636_SourceDataForAppendix/Appendix S4/Panel A/Panel A- annotated.png]

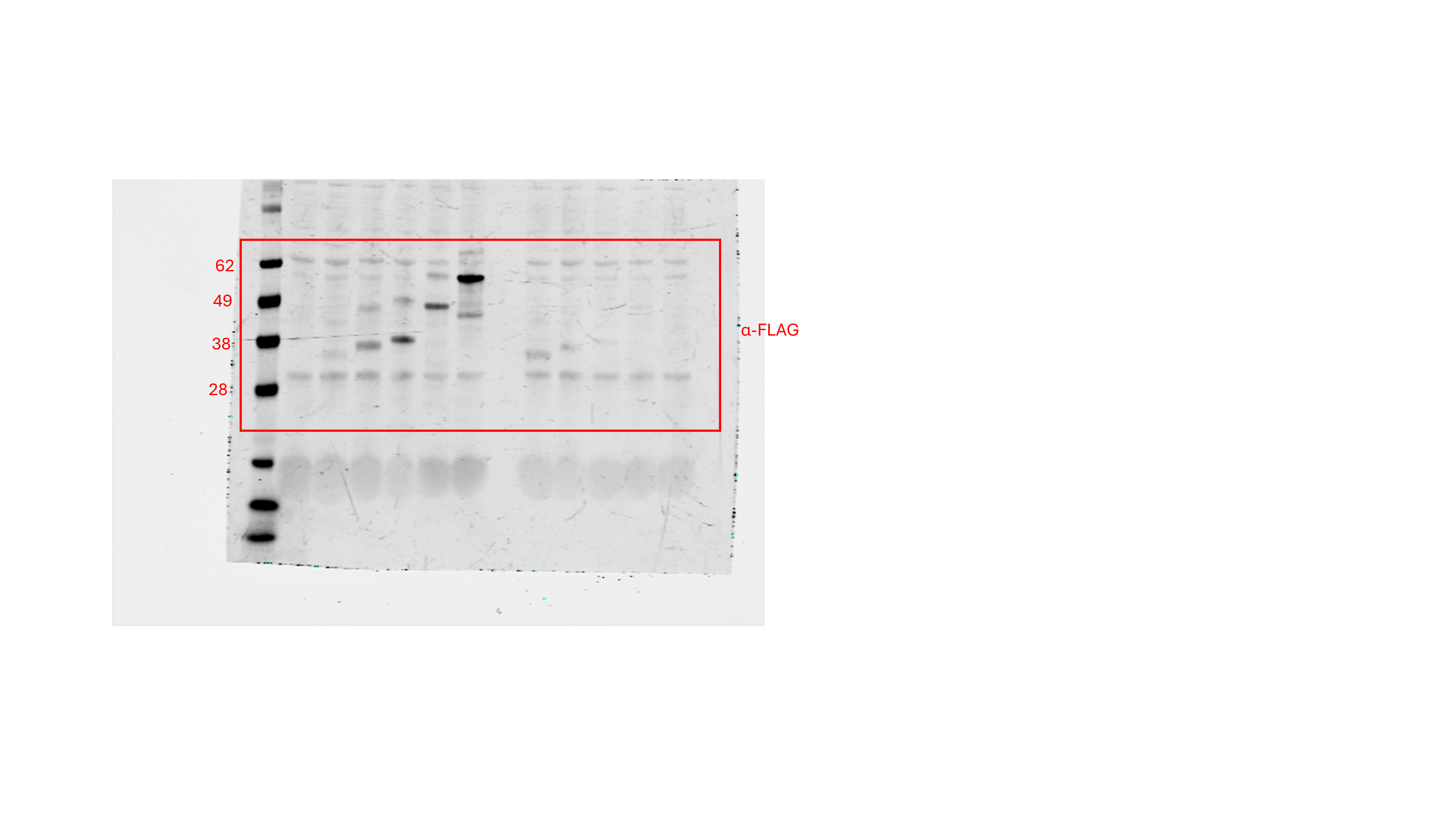

Supplement: Supplementary file 7 — Appendix Source Data [file 44318_2025_548_MOESM7_ESM.zip › EMBO-J-20205-120636_SourceDataForAppendix/Appendix S1/Panel B/WB annotated.png]

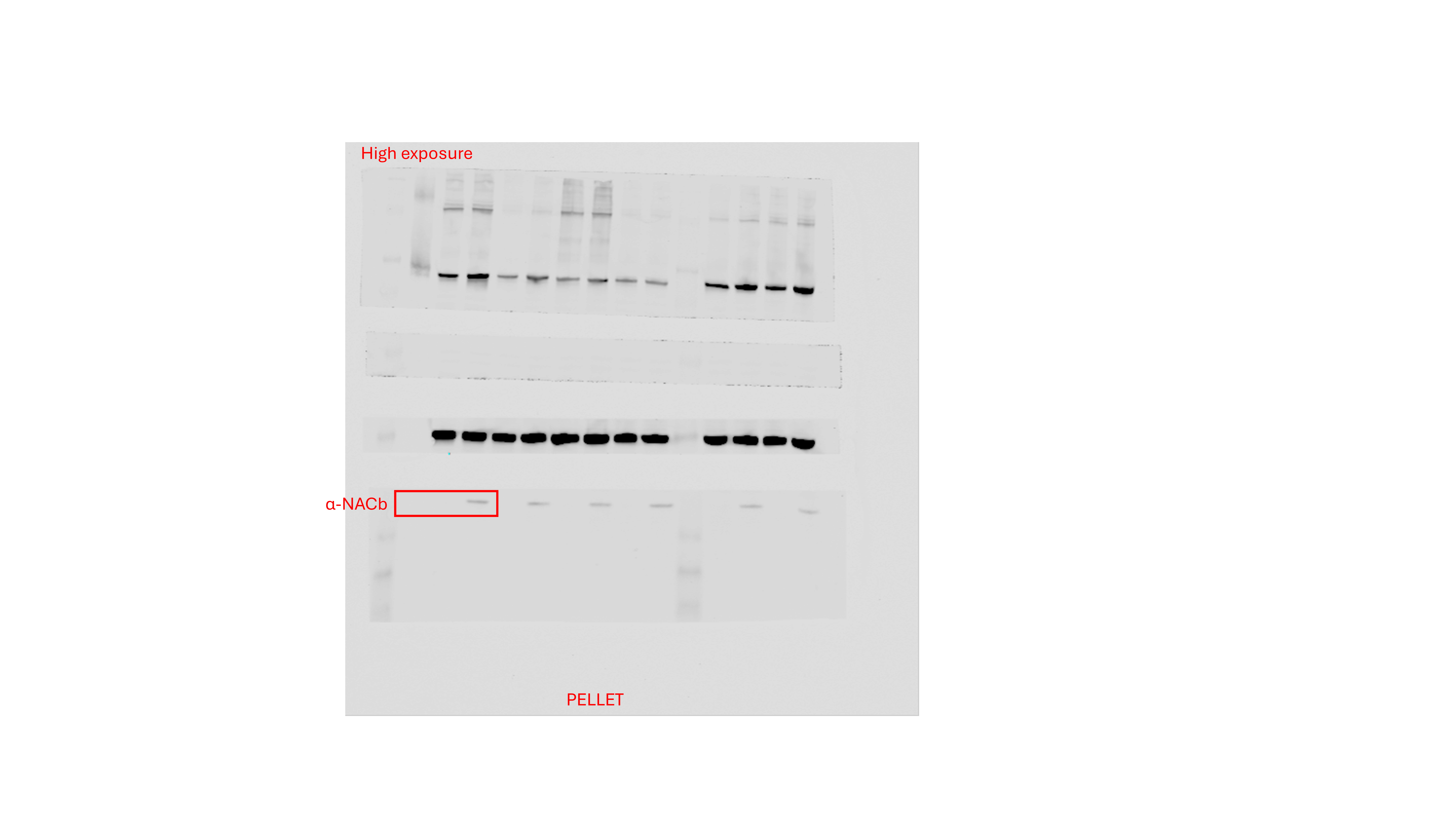

Supplement: Supplementary file 7 — Appendix Source Data [file 44318_2025_548_MOESM7_ESM.zip › EMBO-J-20205-120636_SourceDataForAppendix/Appendix S1/Panel A/right side pellet WB high exposure annotated.png]

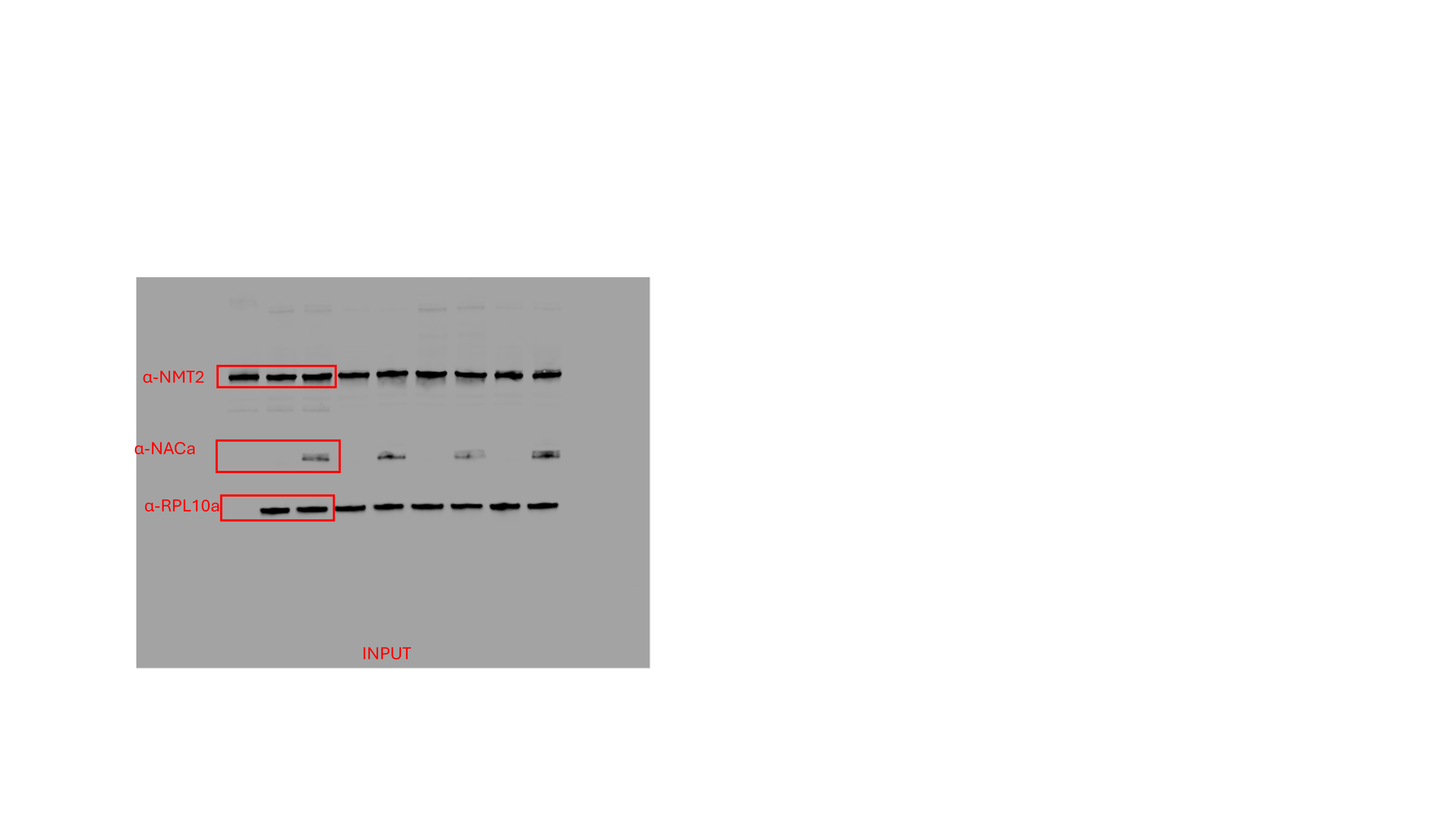

Supplement: Supplementary file 7 — Appendix Source Data [file 44318_2025_548_MOESM7_ESM.zip › EMBO-J-20205-120636_SourceDataForAppendix/Appendix S1/Panel A/right side input WB annotated.png]

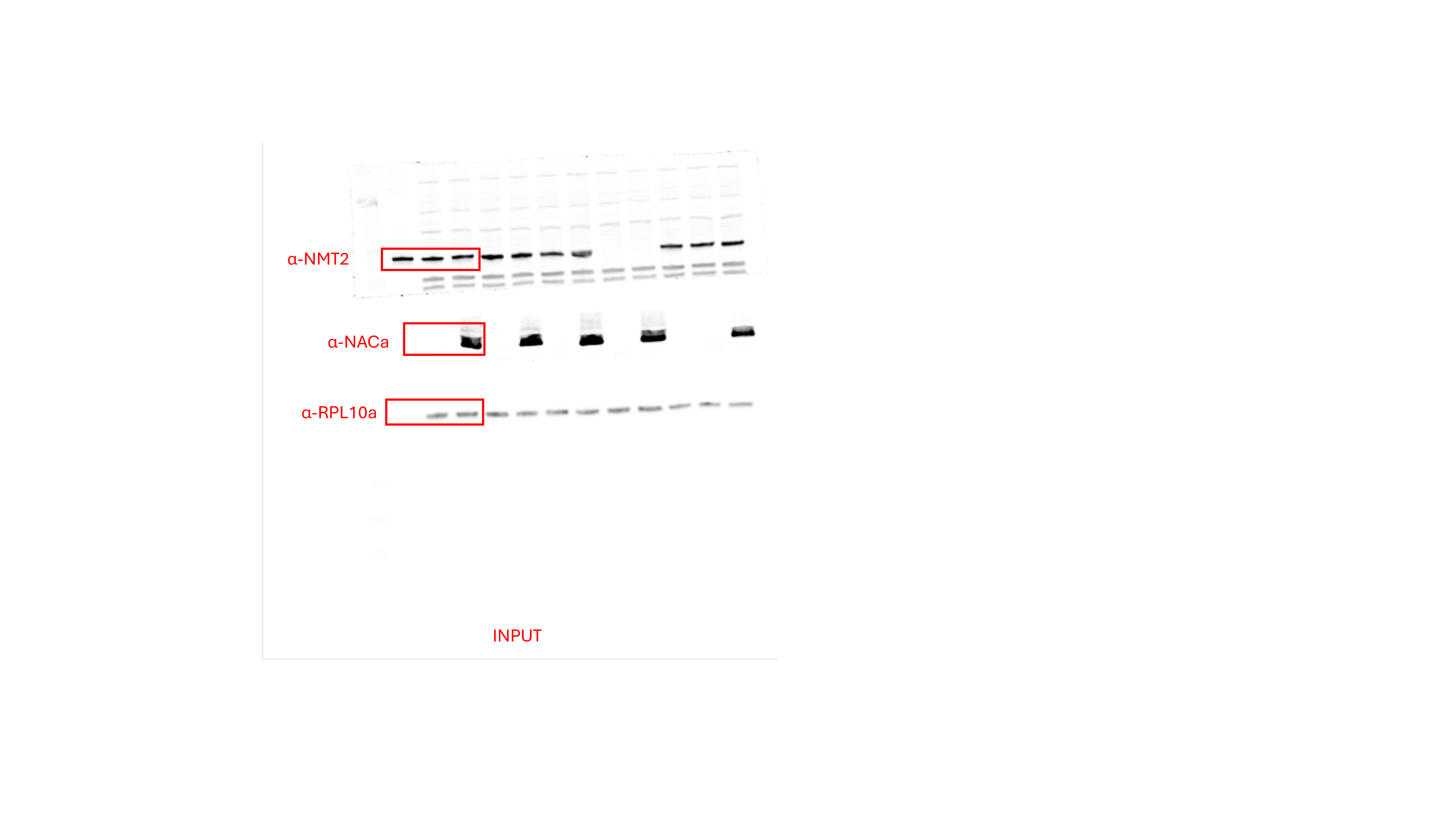

Supplement: Supplementary file 7 — Appendix Source Data [file 44318_2025_548_MOESM7_ESM.zip › EMBO-J-20205-120636_SourceDataForAppendix/Appendix S1/Panel A/left side input WB annotated.png]

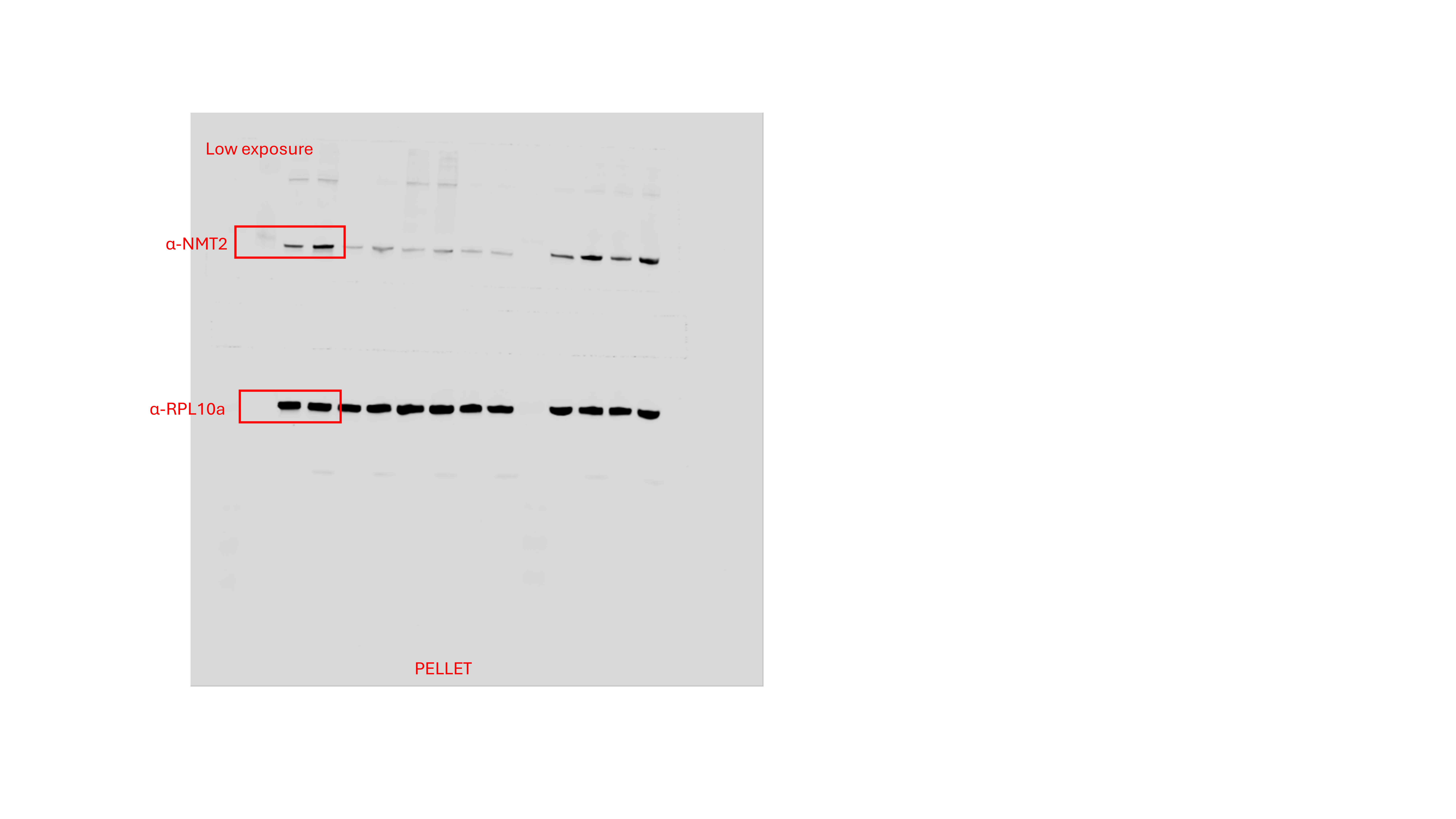

Supplement: Supplementary file 7 — Appendix Source Data [file 44318_2025_548_MOESM7_ESM.zip › EMBO-J-20205-120636_SourceDataForAppendix/Appendix S1/Panel A/right side pellet WB low exposure annotated.png]

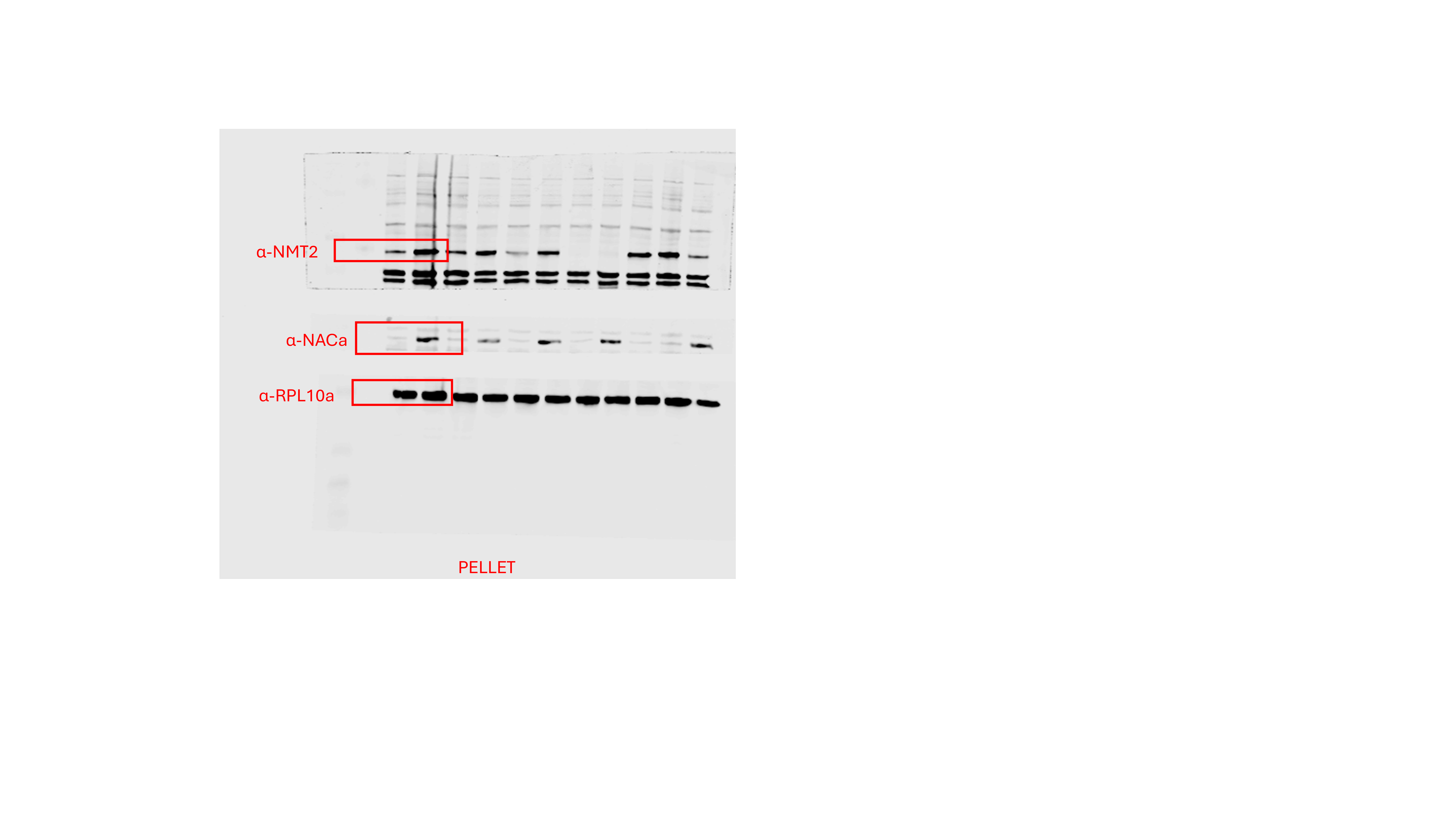

Supplement: Supplementary file 7 — Appendix Source Data [file 44318_2025_548_MOESM7_ESM.zip › EMBO-J-20205-120636_SourceDataForAppendix/Appendix S1/Panel A/left side pellet WB annotated.png]
