# Supplementary material for: NAC couples protein synthesis with nascent polypeptide myristoylation on the ribosome
Source: EMBO J. 2025 Aug 26;44(22):6320–42. doi: 10.1038/s44318-025-00548-4 (PMC12623983; doi:10.1038/s44318-025-00548-4)
Supplement: Supplementary file 8 — Expanded View Figures [file 44318_2025_548_MOESM8_ESM.pdf]

Expanded View Figures

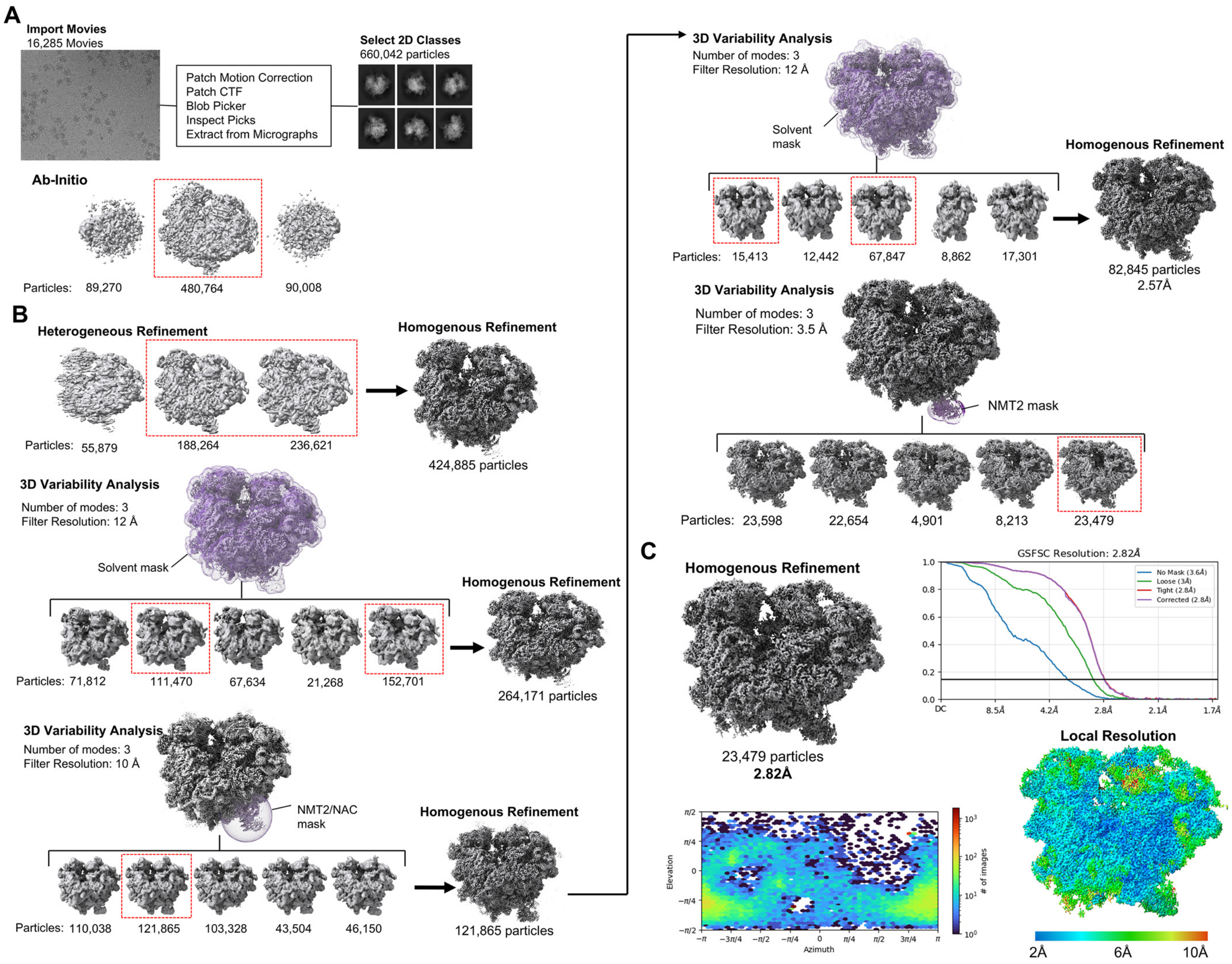

Figure EV1. Cryo-EM processing scheme of RNC_{MARCKS}:NMT2:NAC ternary complex.

(**A**) Movies initially collected were subjected to motion and CTF correction, and particles were identified for 2D classification using blob picker. 2D classes were selected and ab initio was used to separate out full ribosomal particles. (**B**) After initial heterogenous refinement to filter out bad classes, the 3D volumes underwent 4 subsequent rounds of 3D variability analysis using a solvent mask and NMT2/NAC mask sequentially. (**C**) The final volume was constructed from 23,479 particles with an overall resolution of 2.82 Å as determined by gold standard Fourier shell correlation of independent half maps with a cutoff of 0.143. Angular distribution plot and local resolution were determined in cryoSPARC (v4.6.0).

 © The Author(s)

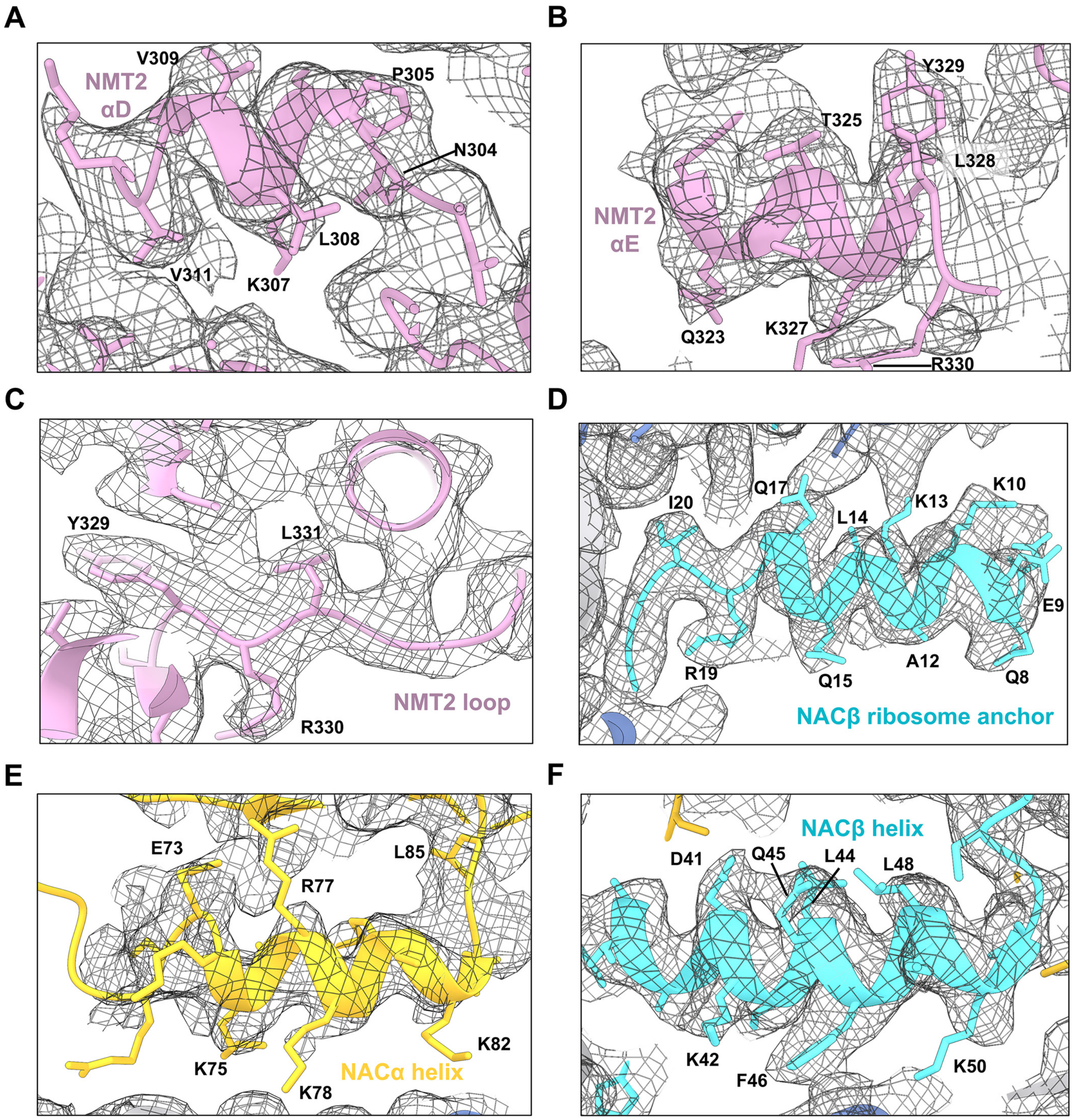

Figure EV2. Structure of NMT2 and NAC within the ternary complex.

(**A**) Cryo-EM map depicts fit of NMT2 αD into density. (**B**) NMT2 helix αE fit into cryo-EM density. (**C**) Cryo-EM map shows fit of the loop region connected to the anchor helix αE of NMT2 into density. (**D**) A close-up of the ribosome anchor of NACβ. (**E**) Close-up view of NACα helix within its ordered dimerization domain. (**F**) Helix within NACβ dimerization domain shown in a close-up view. All cryo-EM densities are shown as mesh and filtered to 3.5 Å.

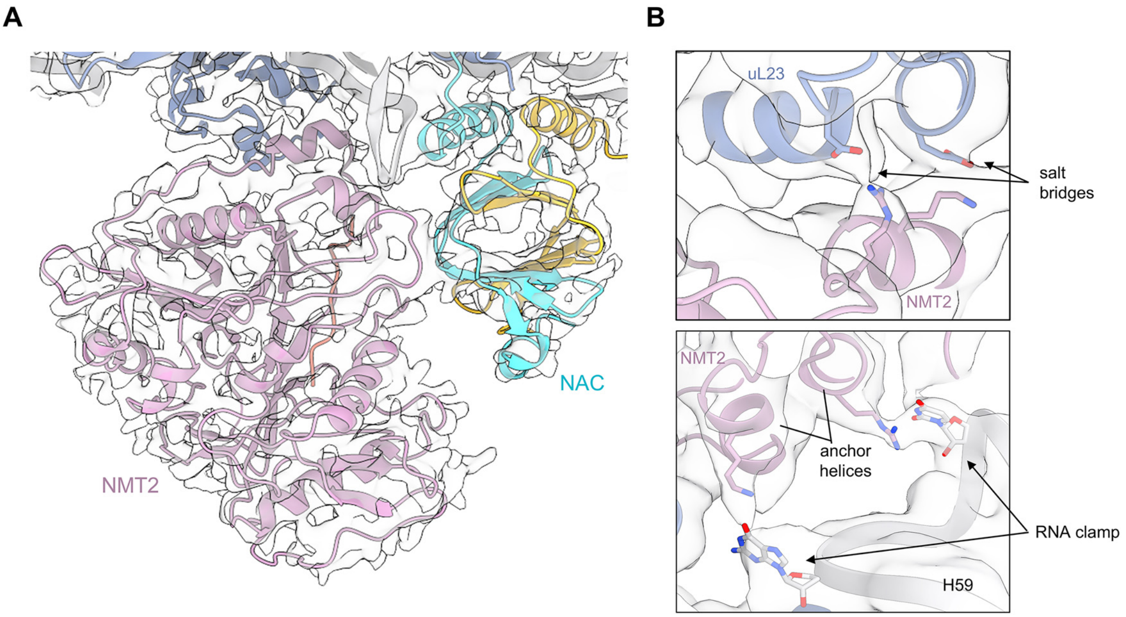

Figure EV3. Fit of NMT2 and NAC models into the cryo-EM density map highlighting the indicated contacts.

(**A**) Overall fit of NAC (cyan, gold) and NMT2 (pink) into cryo-EM density map shown as a transparent surface and filtered to 3.5 Å. (**B**) Close-up views of H59 clamp contacts and uL23 electrostatic contacts show density for both flipped-out bases as well as for interactions with NMT2.

 © The Author(s)

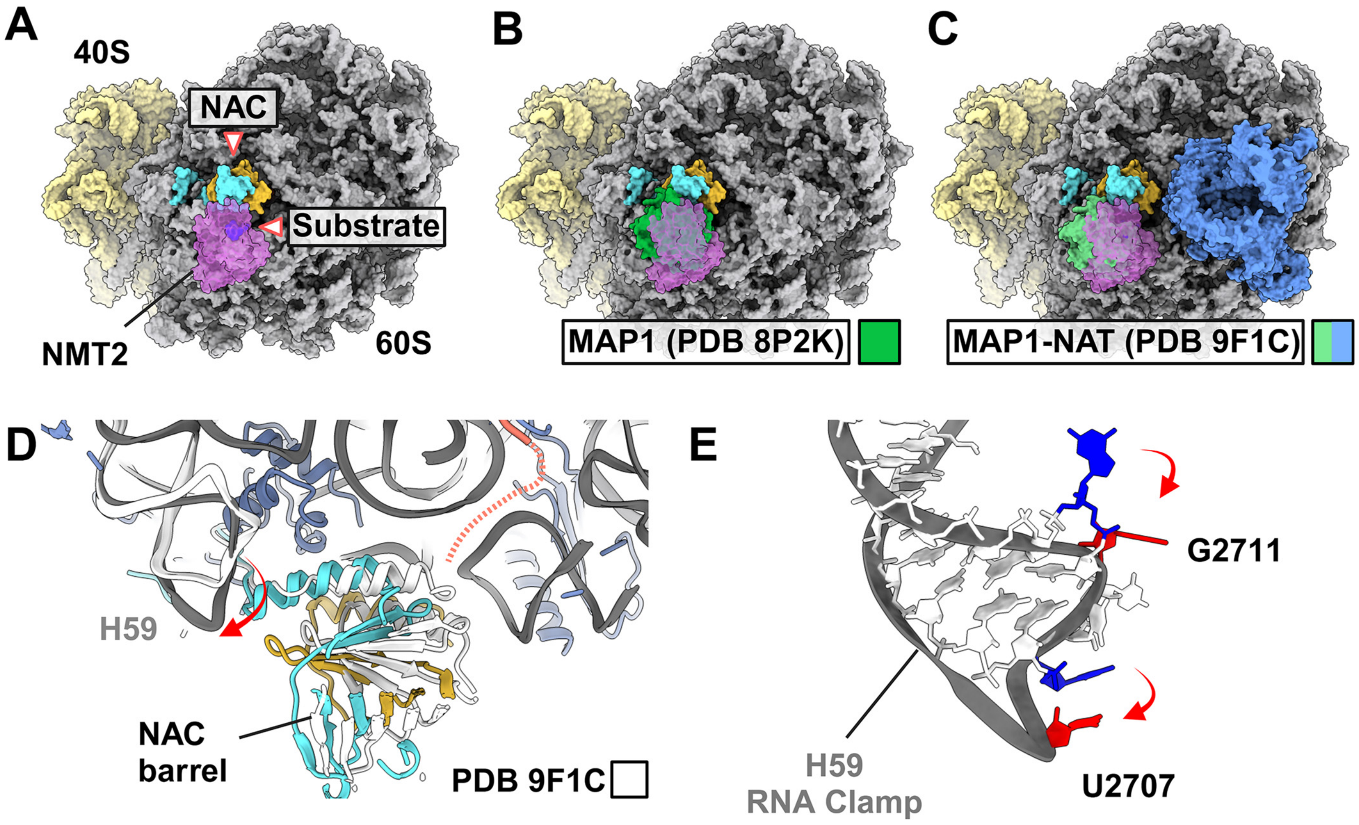

Figure EV4. MAP1 binding overlaps with NMT2 docking and substrate engagement on ribosomes.

(**A**) Surface model of the current NAC-NMT2 structure with substrate bound in the active site (blue). (**B**) Model of MAP1 docked to the current structure showing overlap of MAP1 and NMT2 binding sites. (**C**) Model of MAP1-NAT docked to the current structure depicting differing binding locations for NAT compared to MAP1 and NMT2. (**D**) Conformational change of NAC and H59 (red arrow) between the current structure and the NAC-MAP1 (PDB 8P2K) and the NAC-MAP1-NAT structure (PDB 9F1C). (**E**) Rearrangement of bases of the RNA clamp compared to the NAC-MAP1-NAT structure (PDB 9F1C).

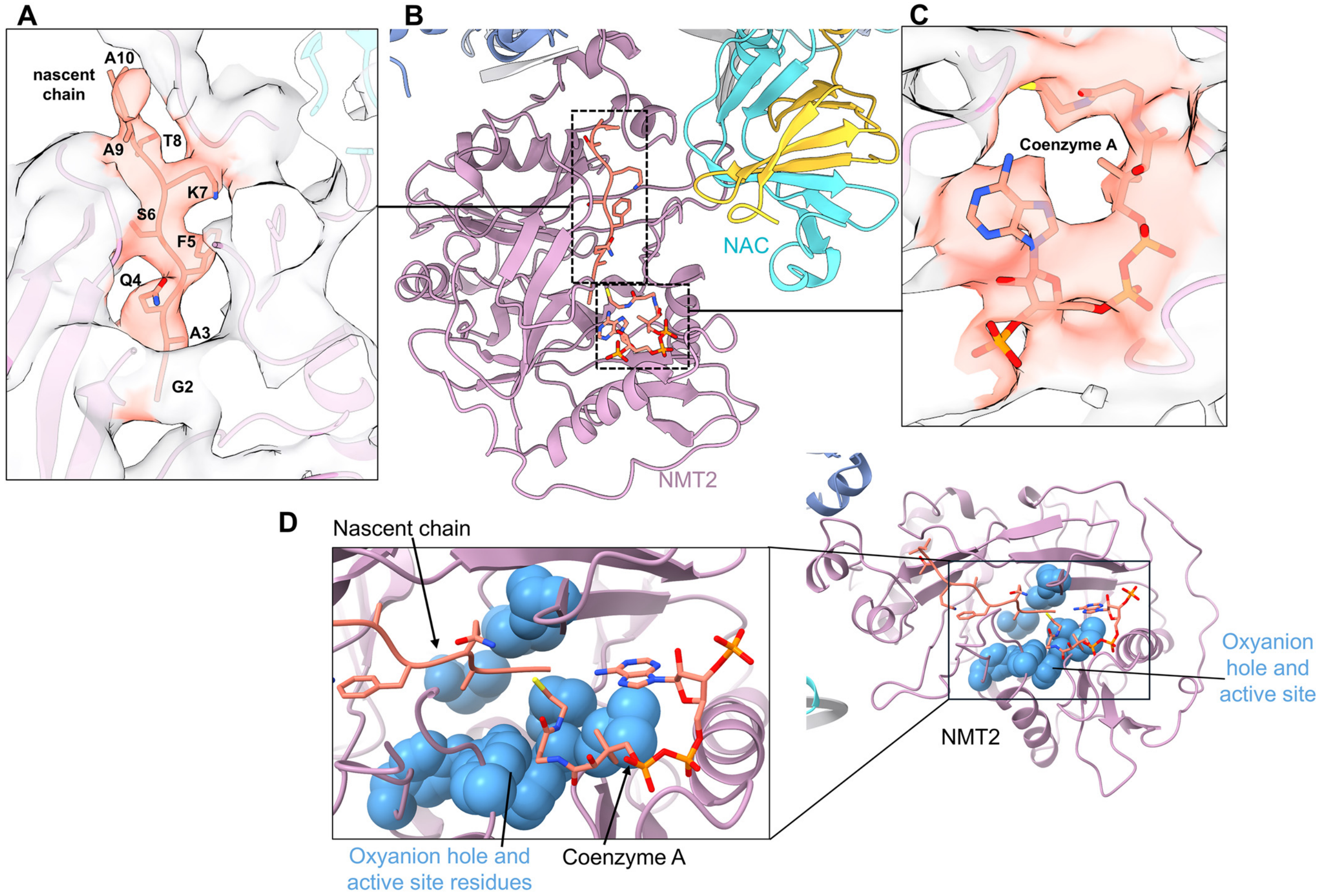

Figure EV5. The nascent chain and coenzyme A are present in the RNC_{MARCKS}:NMT2:NAC ternary complex density.

(**A**) N-terminus of MARCKS is present in cryo-EM density (coral). (**B**) Crystal structure of NMT2 (PDB ID 6PAU) shown docked onto the ribosome containing the substrate nascent chain and coenzyme A. (**C**) Density for coenzyme A present in cryo-EM density map (coral). (**D**) NMT2 showing the oxyanion hole and active site of the enzyme (blue) and where the nascent chain and coenzyme A are positioned during myristoylation. All cryo-EM density has been shown as a transparent surface and filtered to 3.5 Å.

 © The Author(s)

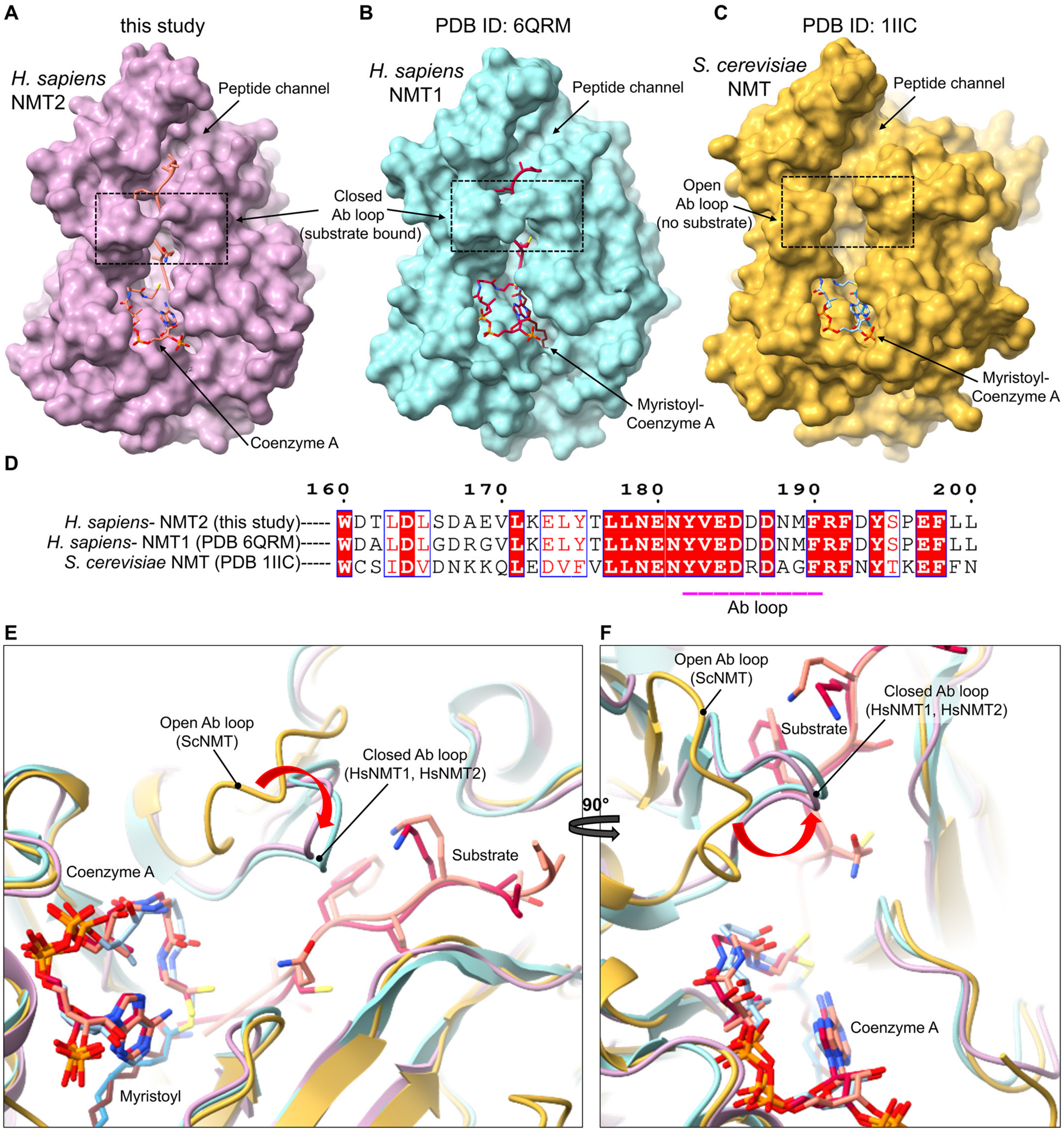

Figure EV6. Comparison of Ab loop conformation of NMT2 with peptide bound NMT1 and apo *S. cerevisiae* NMT.

(A) Surface view of human NMT2 showing a closed Ab loop conformation over a substrate polypeptide (coral). Coenzyme A and the opening to the peptide channel where the substrate would enter from the ribosome polypeptide exit tunnel are also labeled. **(B)** Surface view of human NMT1 showing a closed Ab loop conformation over a substrate polypeptide (magenta). Myristoyl-coenzyme A and the opening to the peptide channel where the substrate would enter from the ribosome polypeptide exit tunnel are also labeled. **(C)** Surface view of *S. cerevisiae* NMT showing an open Ab loop conformation over an empty substrate channel. Myristoyl-coenzyme A (light blue) and the opening to the peptide channel are also labeled. **(D)** Sequence alignment of HsNMT2, HsNMT1, and ScNMT indicating the residues making up the Ab loop (magenta line). **(E)** Close-up view of the conformational change between open and closed Ab loops when a polypeptide substrate is present. NMTs are shown as cartoons (pink, blue, yellow) and overlaid. Substrate, myristoyl-coenzyme A, and coenzyme A are shown as sticks. **(F)** A second view of the open and closed Ab loop conformations in the various NMTs.

© The Author(s)
